# A distal vinyl shift (DVS) through quadruple domino reaction: synthesis of *N*-vinyl benzoheterocyclic scaffolds†

**DOI:** 10.1039/c8ra01478g

**Published:** 2018-03-28

**Authors:** Manickam Bakthadoss, Mohammad Mushaf

**Affiliations:** Department of Chemistry, Pondicherry University Pondicherry 605014 India bhakthadoss@yahoo.com

## Abstract

A conceptually novel Distal Vinyl Shift (DVS) through quadruple domino reaction involving imine formation, oxazole/thiazole/oxazine formation, aza-Michael addition and selective retro oxa/aza-Michael addition leading towards *N*-vinyl benzoxazoles/benzothiazoles/*N*-vinyl 1,3-benzoxazines has been developed for the first time. This reaction is highly stereoselective and was carried out efficiently without using any catalyst as well as column chromatography purification with wide substrate scope in very good to excellent yields.

## Abstract 2

An unprecedented Distal Vinyl Shift (DVS) through quadruple domino reaction has been disclosed *via* the synthesis of *N*-vinyl benzoheterocyclic scaffolds.

## Graphical abstract

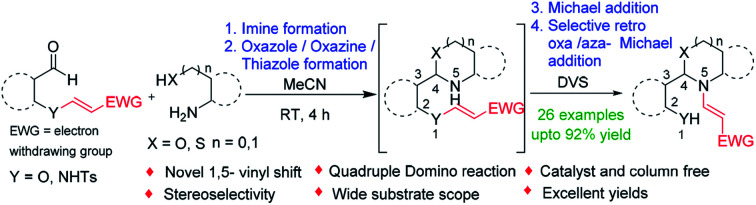

## Introduction

**Fig. 1:**
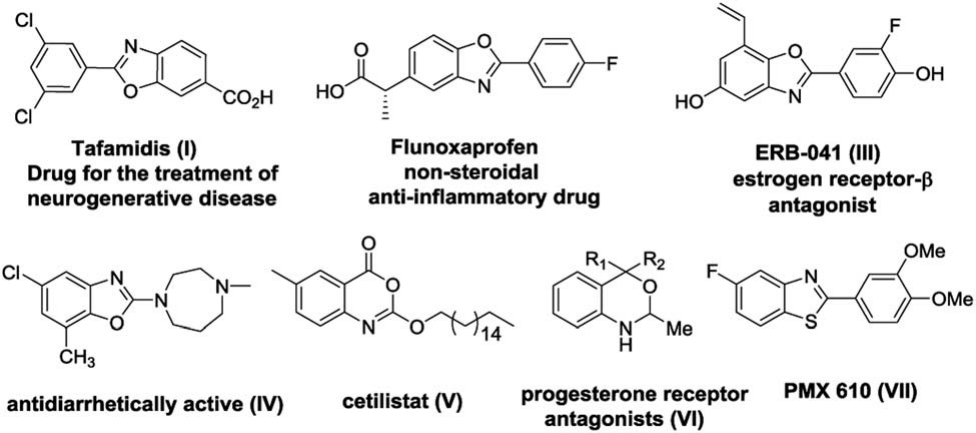
Drug molecules containing benzoxazole, benzothiazole and 1,3-benzoxazine skeleton.

Heterocyclic compounds bearing nitrogen and oxygen atoms are extremely abundant in nature and display a wide array of bioactivities which make them highly useful in the field of pharmaceuticals and agrochemicals owing to a large number of applications. Therefore, synthesis of highly functionalized nitrogen and oxygen containing heterocyclic^1^ compounds possessing interesting medicinal applications is a challenging area in organic synthesis. The synthesis of a complex heterocyclic scaffold utilizes many steps which can be drastically reduced when reactions are carried out in a domino fashion. Domino reactions involve formation of multiple carbon–carbon bonds in a single operation thereby giving access to complex molecules from simple substrates in a straightforward manner.^2^ These reactions are economically as well as environmentally favourable since they do not involve the preparation of intermediates which are considered as energy consuming and waste generating steps in organic transformations. Quadruple domino reactions have attracted considerably less attention when compared to double and triple domino reactions and very few reports are available in the literature.^3^ This manuscript describes the distal vinyl shift^4^ (1,5-vinyl shift) *via* quadruple domino reaction involving imine formation, oxazole/thiazole/oxazine formation, aza-Michael addition and selective retro oxa/aza-Michael addition resulting in the formation of privileged scaffolds such as *N*-vinyl benzoxazoles, benzothiazoles and *N*-vinyl 1,3-benzoxazines. Among various heterocyclic compounds, benzoxazoles, benzothiazoles and benzoxazines structures are present in various natural products as well as in commercially available drugs and are important precursors for the synthesis of more complex organic frameworks.^5^ Benzoxazole and benzothiazole moieties are important components of many biologically active molecules and have many applications in medicinal chemistry. For example benzoxazoles are found to act as melatonin receptor agonists,^6^ amyloidogenesis inhibitors,^7^ rho kinase inhibitors,^8^ anticancer agents,^9^ antimicrobial,^10^ antitumor,^11^ and antibacterial^12^*etc.* Additionally, benzoxazoles have also been utilized in agricultural applications as herbicides,^13^ dye lasers,^14^ in polymer synthesis,^15^ and as fluorescent probes for anion and metal cation sensors.^13^ Benzothiazoles also exist in a wide range of active molecules displaying broad biological activities and are found in functional materials.^16^ Similarly, benzoxazine derivatives are prominent in the domain of synthetic and medicinal chemistry due to their presence in various alkaloids and pharmaceutical agents. Benzoxazines have been used as potent progesterone-receptor agonists, DNA binding antitumor agents,^17^ human leukocyte elastase (HLE) inhibitors,^18^ serine protease inhibitors, as well as fungicidal, anti-inflammatory, and anticonvulsant drugs.^19^ Some of the representative examples of drug candidates containing benzoxazole, benzothiazole and benzoxazine units are shown in Fig. 1.

Due to the above mentioned interesting biological applications of benzoxazoles, benzothiazoles as well as 1,3-benzoxazines, the synthesis of these scaffolds is one of the thrust areas in the field of heterocyclic chemistry.

**Scheme 1:**
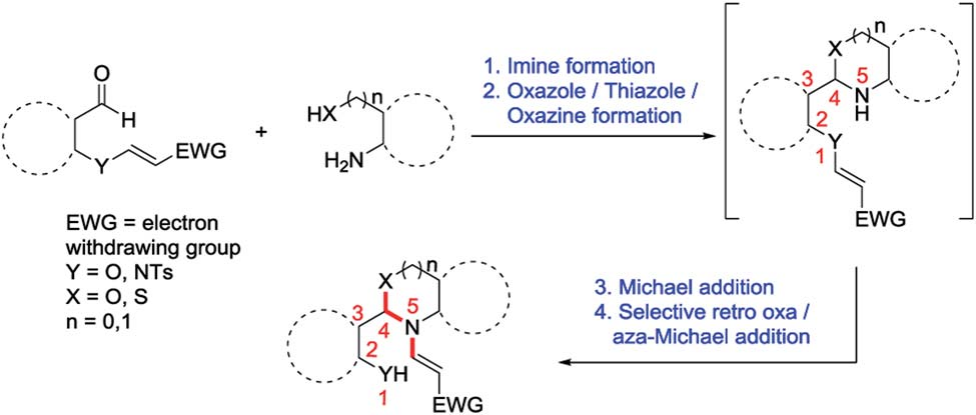
Strategy for the distal vinyl shift.

Moreover, migration of vinyl group from oxygen atom present in the first position to the nitrogen atom present in the fifth position is unprecedented and this kind of conceptually novel distal vinyl shift is new in the field of organic chemistry. Therefore incorporating this novel concept of a distal vinyl shift in the synthesis of above mentioned privileged scaffolds is very interesting and useful. We envisaged that we can incorporate this conceptually novel distal vinyl shift in the formation of privileged scaffolds such as *N*-vinyl benzoxazoles/benzothiazoles/*N*-vinyl benzoxazines *via* quadruple domino reaction involving imine formation, oxazole/thiazole/oxazine formation, aza-Michael addition and selective retro oxa/aza-Michael addition as shown in Scheme 1.

## Results and discussion

In continuation of our interest in the field of domino reactions,^20^ we herein report a conceptually novel 1,5-vinyl shift through a quadruple domino reaction using *O*-vinyl salicylaldehydes and 2-amino phenol/2-aminothiophenol/2-aminobenzyl alcohol in a stereoselective fashion for the first time. To execute our idea of demonstrating a novel 1,5-vinyl shift, we have taken *O*-vinyl salicylaldehyde 1a and 2-aminophenol 2a as model substrates to obtain the desired benzoxazole derivatives incorporating the 1,5-vinyl shift. To achieve our goal, we treated the methyl (*E*)-3-(2-formylphenoxy)acrylate 1a and 2-aminophenol 2a in dichloromethane without any catalyst at room temperature for 3 h successfully provided the desired benzoxazole derivative 3a in 30% yield. It is important to mention here that as per our hypothesis, during the process, oxygen attached vinyl group got shifted from oxygen atom to nitrogen atom intramolecularly in the compound which presumably proceeded *via* 1,5-vinyl shift (see the mechanism). During the course of the reaction, the stereochemistry of *O*-vinyl double bond is maintained in the *N*-vinyl product which clearly indicates that the reaction is stereoselective in nature. Furthermore, the product formation 3a clearly shows that the reaction proceeded *via* a novel quadruple domino reaction consisting of imine formation, cyclization, aza-Michael addition and selective retro oxa/aza-Michael addition reaction. Another important feature of this reaction is that the reaction is carried out at room temperature without using any catalyst. In order to improve the yield of this domino reaction, we have screened various solvents such as ethanol, methanol, THF, chloroform, toluene, dioxane, and acetonitrile. The best result was obtained when the reaction was carried out in acetonitrile solvent (80% yield) at room temperature. The product precipitated after the completion of the reaction which was filtered and washed with 5% EtOAc/hexane to obtain the pure product (3a). It is worth mentioning here that the desired product (3a) was obtained without workup and column chromatography purification in very good yield which holds significance from a green chemistry point of view. Interestingly, this new protocol generates one new chiral centre and creates three new bonds (two C–N and one C–O) with the migration of a vinyl group from oxygen atom present in the first position to the secondary nitrogen atom present in the fifth position *via* 1,5-vinyl shift (see Scheme 1).

In order to establish the generality of the present protocol with the optimized condition in hand, we have explored the scope of the substrates where we have treated a variety of substituted *O*-vinyl salicylaldehydes (1) with 2-aminophenol derivatives (2) using the standardized reaction condition and the results are summarized in Table 1.

**Table 1:** Substrate scope of the *N*-vinyl benzoxazole/benzothiazole derivatives^a^^,^^b^

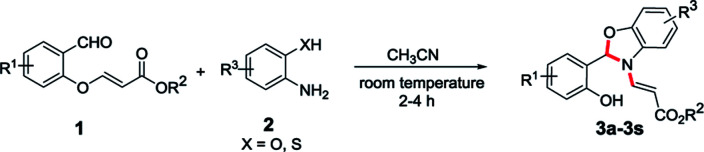
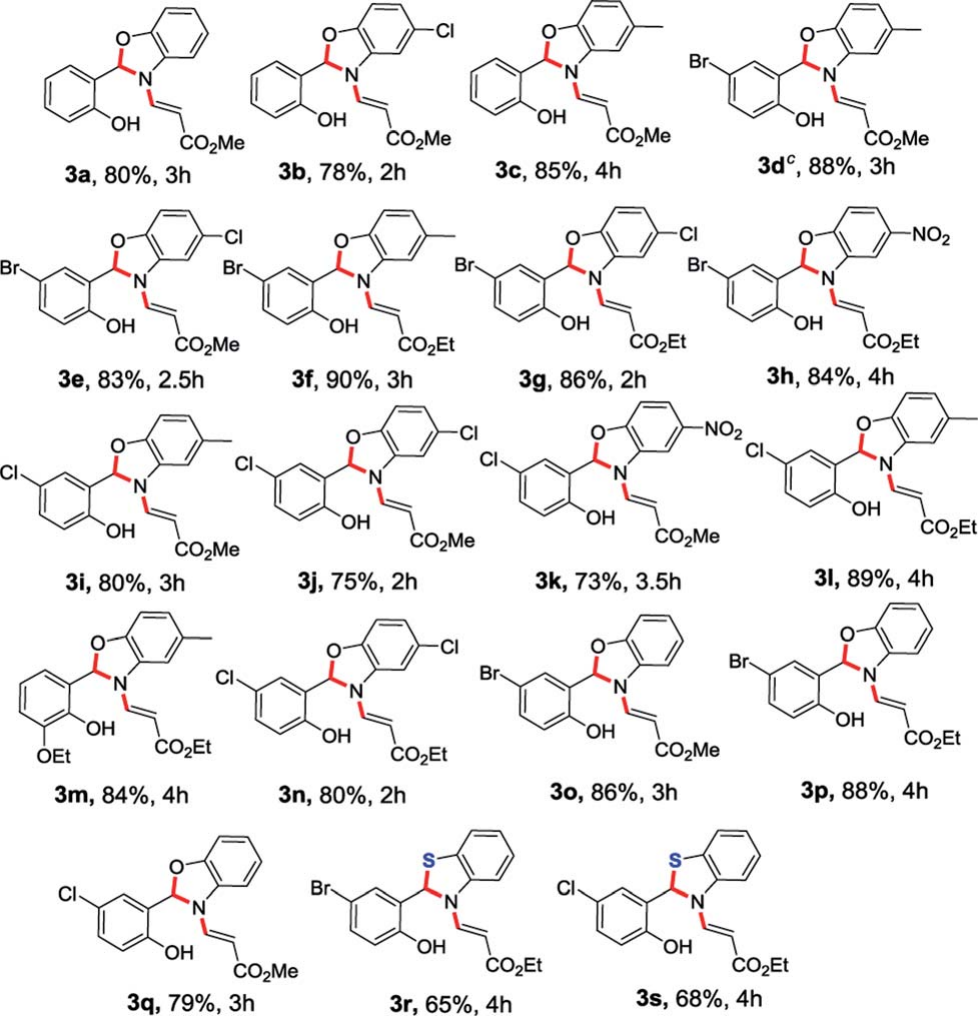

a All reactions were conducted with 1 mmol of 1, 1 mmol of 2 in MeCN (10 ml) at room temperature for the time mentioned for each product (see ESI).

b Yields of the isolated products are given.

c Structure confirmed by single crystal X-ray analysis.

As we anticipated, all the starting materials 1 and 2 were smoothly converted into the corresponding desired benzoxazole derivatives (3a–3q) in excellent yields (73–90%). To further extend the scope of the reaction, we have also extended the methodology to the sulphur version. Accordingly, the treatment of aminothiophenol with *O*-vinyl salicylaldehyde derivatives smoothly provided the desired compounds 3r and 3s in 65% and 68% yields respectively (Table 1). The *O*-vinyl salicylaldehyde derivatives (1) carrying both electron withdrawing and electron releasing substituents worked well and were transformed into corresponding *N*-vinyl benzoxazole derivatives in very good yields. The generality of the present protocol was also extended by using various amine components (2a–2e). When 2-aminophenol derivatives bearing –Me and –Cl groups were used, the desired products were obtained in very good yields. In addition, 2-amino phenol possessing a strong electron withdrawing NO_2_ group also afforded the *N*-vinyl benzoxazoles (3h and 3k) in very good yields. In general, this methodology worked smoothly with a wide variety of *O*-vinyl salicylaldehydes and substituted *o*-aminophenols and 2-aminothiophenols to give the benzoxazole as well as benzothiazole products with very good yields.

To enhance the scope of the reaction even further, we have also used 2-aminobenzyl alcohol (4) with *O*-vinyl salicylaldehyde (1). The treatment of *O*-vinyl salicylaldehyde derivatives (1) with 2-aminobenzyl alcohol (4) in acetonitrile at room temperature successfully led to desired *N*-vinyl 1,3-benzoxazines (5a–e) in excellent yields (84–92%) as shown in the Table 2.

**Table 2:** Substrate scope of the *N*-vinyl benzoxazine derivatives^a^^,^^b^

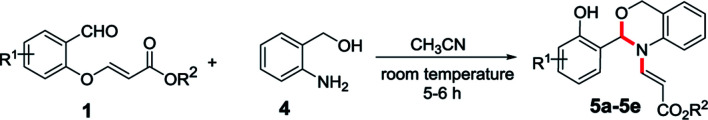
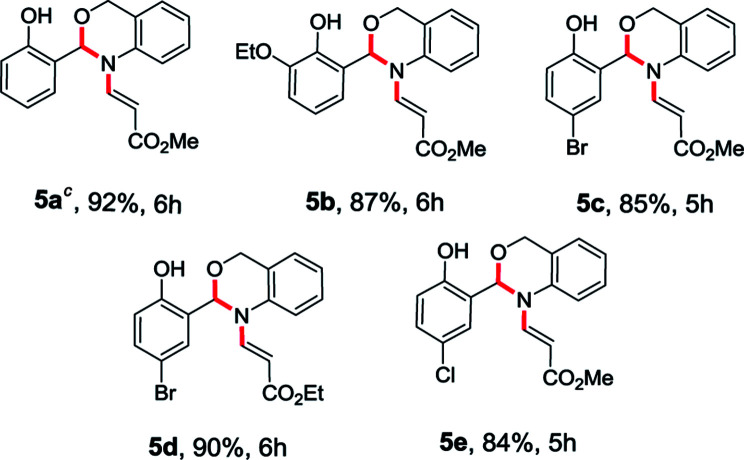

a All reactions were conducted with 1 mmol of 1, 1 mmol of 4 in MeCN (10 ml) at room temperature for the time given for each product (see ESI).

b Yields of the isolated products are given.

c Structure confirmed by single crystal X-ray analysis.

After successfully achieving the 1,5-vinyl shift from oxygen atom to nitrogen atom, we were interested to check this novel 1,5-vinyl shift from nitrogen atom to another nitrogen atom which is in fifth position. Accordingly, we synthesized the requisite starting material such as *N*-tosyl derivative of *N*-vinyl salicylaldehyde (6) and treated with 2-aminophenol in acetonitrile which successfully led to the corresponding *N*-vinylated benzoxazole derivatives (7a and 7b) and clearly indicate that the 1,5-vinyl shift is possible between nitrogen atoms present in first and fifth positions of the molecule (Table 3).

**Table 3:** *N*-vinyl shift from nitrogen to nitrogen atom^a^^,^^b^

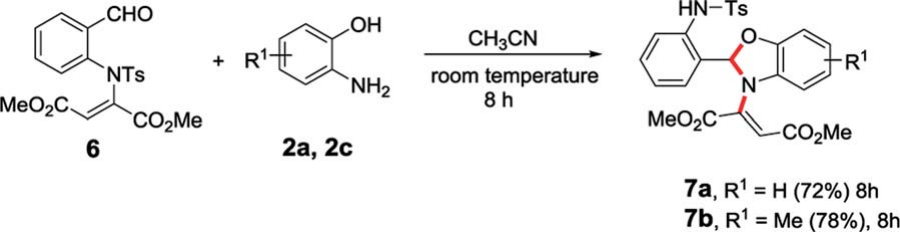

a All reactions were conducted with 1 mmol of 6, 1 mmol of 2 in MeCN (10 ml) at room temperature for 8 h.

b Yields of the isolated products are given.

**Scheme 2:**
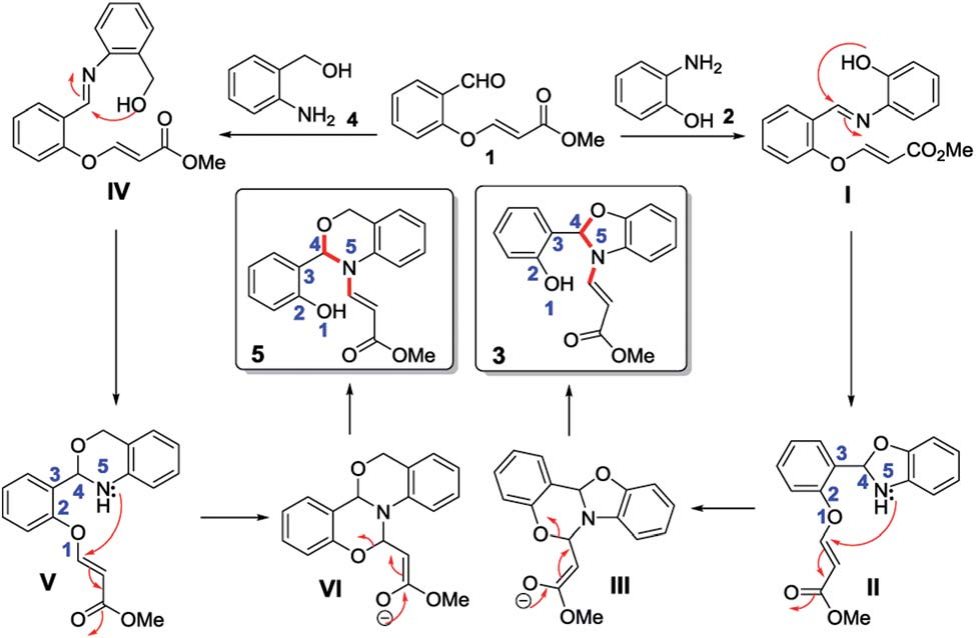
Proposed mechanistic pathway.

The plausible mechanism for the 1,5-vinyl shift and the formation of *N*-vinyl benzoxazole/*N*-vinyl 1,3-benzoxazine from *O*-vinyl salicylaldehyde and 2-aminophenol/2-aminobenzyl alcohol involving quadruple domino reaction is shown in Scheme 2.

This reaction proceeds initially with the formation of imine followed by the attack of the phenolic oxygen on the imine carbon leading to the formation of dihydrobenzoxazole intermediate (II). Generally, the dihydrobenzoxazole moiety undergoes aerial oxidation very easily to form the aromatic benzoxazole, but in our case, the nitrogen on the benzoxazole moiety attacks the double bond of the *O*-vinylic moiety in an aza-Michael addition which leads to the intermediate (III). In intermediate (III), there are two possibilities for cleavage due to retro Michael addition (cleavage of O–C and N–C bond). However, we observed only the cleavage of O–C bond (except in case of starting material 6) rather than N–C bond which clearly indicate the chemoselective cleavage of this reaction *via* retro oxa-Michael reaction instead of retro aza-Michael reaction thus leading to the formation of *N*-vinyl benzoxazoles (3) as shown in Scheme 2. Similarly, in the case of 2-aminobenzyl alcohol (4), the aldehyde reacts with the amine to form imine which inturn cyclizes to form intermediate (V). The intermediate (V) further undergoes aza-Michael addition to give a tetracyclic intermediate (VI). Subsequently the intermediate (VI) undergoes retro oxa-Michael addition by the selective cleavage of phenolic O–C bond to provide corresponding *N*-vinyl derivative 5 as shown in Scheme 2.

**Fig. 2:**
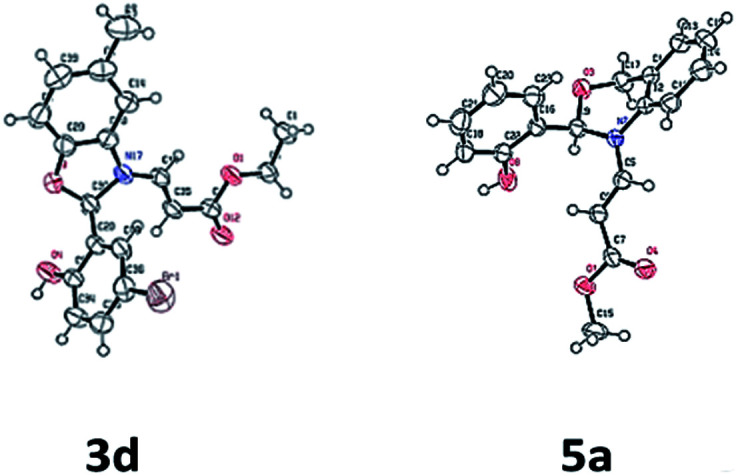
ORTEP diagrams of compounds 3d and 5a.^21^

The structure of the compounds 3d and 5a were further confirmed by single crystal X-ray analyses and the ORTEP diagrams are shown in Fig. 2.

## Conclusions

We have developed a conceptually novel strategy for the synthesis of *N*-vinyl benzoheterocyclic scaffolds by means of intramolecular distal vinyl migration. This unprecedented 1,5-vinyl shift proceeds through quadruple domino reaction *via* imine formation, oxazole/thiazole/oxazine formation, aza-Michael addition and retro oxa/aza-Michael addition in a single process thereby providing an efficient access to functionalized *N*-vinyl benzoxazoles/benzothiazoles/1,3-benzoxazines. Wide varieties of *N*-vinyl benzoxazole/benzothiazole and 1,3-benzoxazine derivatives were constructed in good to excellent yields. Interesting features of this new protocol also includes workup and column free synthesis, absence of catalyst and use of readily available starting materials.

## Conflicts of interest

There are no conflicts to declare.

## Supplementary Material

RA-008-C8RA01478G-s001

RA-008-C8RA01478G-s002
