# Supplementary material for: A distal vinyl shift (DVS) through quadruple domino reaction: synthesis of N-vinyl benzoheterocyclic scaffolds
Source: RSC Adv. 2018 Mar 28;8(22):12152–6. doi: 10.1039/c8ra01478g (PMC9079281; doi:10.1039/c8ra01478g)
Supplement: RA-008-C8RA01478G-s001 [file RA-008-C8RA01478G-s001.pdf]

Electronic Supplementary Material (ESI) for RSC Advances.
This journal is © The Royal Society of Chemistry 2018

A Distal Vinyl Shift through Quadruple Domino Reaction: Synthesis of *N*-vinyl Benzoheterocyclic Scaffolds

Manickam Bakthadoss,* Mohammad Mushaf

[*] *Department of Chemistry, Pondicherry University, Pondicherry-605 014, India*

E-mail: bhakthadoss@yahoo.com

Supplementary Information

Table of Contents

1. General Consideration.. 2

2. Typical experimental procedure for the synthesis of compounds 1 and 6……………………...2

3. Typical experimental procedure for the synthesis of compounds **(3a-s)** and **(5a-e)**…….……..3

4. Typical experimental procedure for the synthesis of compounds **(7a-b)**…………………….....3

5. Analytical Data of the Products……………………………………………………………..….3

6. X-ray Analysis of compound **3d** & **5a** ……...14

7. ^{1}H, ^{13}C NMR & ESI-HRMS spectra for the compounds (**3a-s, 5a-e and 7a-b**)......................17

General Consideration:

Commercial reagents were used without further purification. IR spectra were recorded on a Perkin Elmer-FTIR spectrometer using solid samples as KBr plates. For compounds ^{1}H NMR (400 MHz, $CDCl_3$) and ^{13}C NMR (100 MHz, $CDCl_3$) spectra were recorded in deuterochloroform with one drop of DMSO-d_6 (some compounds were recorded in pure DMSO-d_6)on a Bruker 400 MHz spectrometer using tetramethylsilane (TMS, δ = 0) as an internal standard at room temperature. Mass spectra were recorded on Agilent 1200 LC/MS-6110 mass spectrometer. Aldehydes, propiolates and aminophenols were purchased from Sigma Aldrich. Compounds spectral data and copy of ^{1}H, ^{13}C NMR and ESI-HRMS spectra of all compounds **3a-s, 5a-e** and **7a-b** are listed below (pages 17-95).

Preparation of vinylogous carbonates (1)[1]

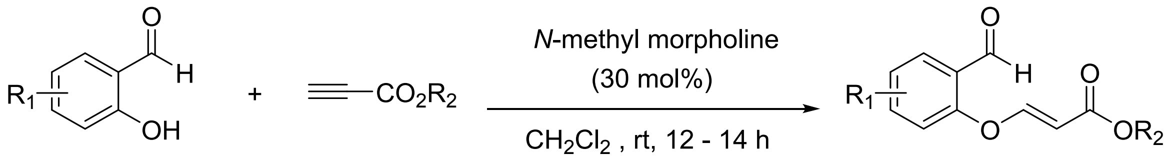

To a solution of salicyladehyde (1 equiv) in dichloromethane was added *N*-methyl morpholine (30 mol %) and the mixture stirred at room tempertaure for 10 minutes. Alkyl propiolates (1.1 equiv) were added and the reaction kept for stirring for 12-14h. The progress of the reaction was monitored by TLC. DCM was removed using rotary evaporator and the mixture was quenched by addition of water and 5% dilute HCl. The aqueous layer was extracted with ethyl acetate. The organic layer was washed with brine, dried over Na_2SO_4, filtrated, and concentrated under reduced pressure to give the crude product. The crude product was purified by silica gel column chromatography (60-120 mesh) [ethyl acetate / hexanes (0.5:9.5)] to give the final compound.

References

1. (a) V. Srinivas, M. Koketsu. *J. Org. Chem*. 2013, **78**, 11612-11617.

(b) L.-Q. Lu, F. Li, J. An, J.-J. Zhang, X.-L. An, Q.-L. Hua, W.-J. Xiao, *Angew. Chem., Int. Ed.* 2009, **48**, 9542.

Typical experimental procedure for the synthesis of compounds (3a-s) and (5a-e)

A mixture of methyl (*E*)-3-(2-formylphenoxy)acrylate (**1**, 1mmol), and 2-aminophenol or 2-aminothiophenol or 2-aminobenzylalcohol (**2** or **4,** 1 mmol) in acetonitrile (10 mL) was placed in a round bottom flask and stirred at room temperature for 2-4 h. The reaction was monitored by the TLC. After the completion of the reaction (based on the dissapearance of the starting material), the reaction was stopped and the precipitate was filtered and washed with ethylacetate : hexanes (0.5 : 9.5) to afford **3** or **5** as the final compound.

Typical experimental procedure for the synthesis of compounds (7a-b)

A mixture of dimethyl 2-((*N*-(2-formylphenyl)-4-methylphenyl)sulfonamido)fumarate (**6**, 1mmol), and 2-aminophenol (**2**, 1 mmol) in acetonitrile (10 mL) was placed in a round bottom flask and stirred at room temperature for 8h. The reaction was monitored by the TLC. After the completion of the reaction (based on the dissapearance of the starting material), the reaction was stopped and the precipitate was filtered and washed with ethylacetate : hexanes (0.5 : 9.5) to afford the final compound.

Analytical Data of the Products

Methyl (*E*)-3-(2-(2-hydroxyphenyl)benzo[d]oxazol-3(2H)-yl)acrylate (3a)

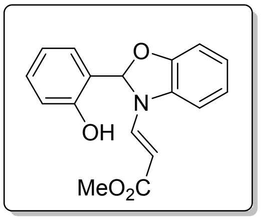

Yield : 80%; white solid; M.P. = 181-182 °C; Reaction time: 3 h; ^{1}H NMR (400 MHz, $CDCl_3$) : δ 3.72 (s, 3H), 4.92 (d, J = 13.6 Hz, 1H), 6.86 – 7.55 (m, 8H), 7.97 (d, J = 13.6 Hz, 1H), 9.73 (s, 1H) ^{13}C NMR (100 MHz, $CDCl_3$) : δ 50.51, 90.67, 92.09, 107.38, 108.70, 115.94, 119.40, 120.88, 120.98, 122.67, 126.67, 130.91, 131.89, 137.78, 149.78, 155.17, 168.12; IR (KBr) : 1635, 1660, 3202, 3482; HRMS calculated for $C_{17}H_{15}NO_4$ $[M+H]^+$ 298.1077, found 298.1068.

Methyl (*E*)-3-(5-chloro-2-(2-hydroxyphenyl)benzo[d]oxazol-3(2H)-yl)acrylate (3b)

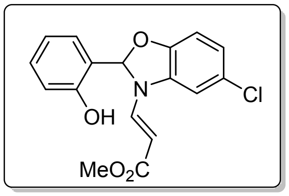

Yield : 78%; white solid; M.P. = 202-203 ˚C; Reaction time: 2 h; ^{1}H NMR (400 MHz, $CDCl_3$) : δ 3.55 (s, 3H), 4.66 (d, J = 13.6 Hz, 1H), 6.80 – 7.48 (m, 8H), 7.92 (d, J = 13.6Hz, 1H), 10.29 (s,1H); ^{13}C NMR (100 MHz, $CDCl_3$) : δ 50.86, 92.33, 93.25, 108.96, 109.50, 116.25, 119.72, 120.93, 122.22, 125.42, 127.76, 131.81, 133.85, 138.27, 148.97, 155.78, 167.34 ; IR (KBr) : 1655, 1680, 3226, 3418 cm^{-1}; HRMS calculated for $C_{17}H_{14}ClNO_4$ $[M+H]^+$ 332.0687, found 332.0683.

Methyl(E)-3-(2-(2-hydroxyphenyl)-5-methylbenzo[d]oxazol-3(2H)-yl)acrylate (3c)

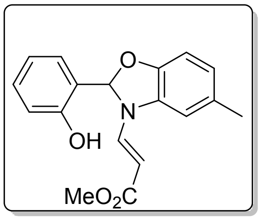

Yield : 85%; white solid; M.P. = 202-203 ˚C; Reaction time: 4 h; ^{1}H NMR (400 MHz, $CDCl_3$) : δ 2.33 (s, 3H), 3.63 (s, 3H), 4.79 (d, J = 16 Hz, 1H), 6.63-7.20 (m, 8H), 7.83 (d, J = 12Hz, 1H), 9.72 (s, 1H); ^{13}C NMR (75 MHz, $CDCl_3$) : δ 20.57, 50.18, 90.33, 91.57, 95.41, 107.91, 107.98, 115.60, 119.06, 120.73, 122.48, 126.30, 130.34, 130.55, 131.49, 137.39, 147.44, 154.87, 167.69; IR (KBr) : 1632, 1667, 3231, 3455 cm^{-1}; HRMS calculated for $C_{18}H_{17}NO_4$ $[M+H]^+$ 312.1234, found 312.1235

Methyl(E)-3-(2-(5-bromo-2-hydroxyphenyl)-5-methylbenzo[d]oxazol-3(2H)-yl)acrylate (3d)

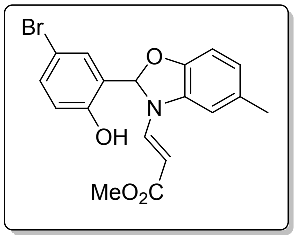

Yield : 88%; white solid; M.P. = 202-203 ˚C; Reaction time: 3 h; ^{1}H NMR (400 MHz, $CDCl_3$) : δ 2.27 (s, 3H), 3.54 (s, 3H), 4.67 (d, J = 13.2 Hz, 1H), 6.66 (s, 2H), 6.87 (d, J = 8.4Hz, 1H), 7.02 (s, 2H), 7.12 (s, 1H), 7.31 (d, J = 7.6Hz, 1H), 7.81 (d, J = 13.6Hz, 1H), 10.46 (s, 1H) ; ^{13}C NMR

(100 MHz, $CDCl_3$) : δ 20.70, 50.35, 78.30, 78.96, 89.71, 91.87, 108.12, 108.95, 110.38, 118.13, 122.89, 123.44, 129.03, 130.76, 131.49, 133.62, 137.76, 147.36, 154.68, 167.12; IR (KBr) : 1628, 1661, 3227, 3443 cm^{-1}; HRMS calculated for $C_{18}H_{16}BrNO_4$ $[M+H]^+$ 390.0329, found 390.0327.

Methyl (E)-3-(2-(5-bromo-2-hydroxyphenyl)-5-chlorobenzo[d]oxazol-3(2H)-yl)acrylate (3e)

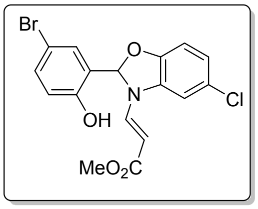

Yield : 83%; white solid; M. P : 201-202 °C; Reaction time : 2.5 h; ^1H NMR (400 MHz, $CDCl_3$) : δ 3.55 (s, 3H), 4.73 (d, J = 13.6Hz, 1H), 6.64 – 7.74 (m, 7H), 7.78 (d, J = 13.6Hz, 1H), 10.28 (s, 1H) ; ^{13}C NMR (100 MHz, $CDCl_3$) : δ 50.46, 90.77, 93.56, 108.16, 109.06, 110.61, 117.95, 121.88, 122.69, 125.71, 129.10, 133.02, 133.68, 137.22, 148.27, 154.56, 167.05; IR (KBr) : 1631, 1670, 3181, 3467 cm^{-1}; HRMS calculated for $C_{17}H_{13}BrClNO_4$ $[M+H]^+$ 409.9792, found 409.9789

Ethyl (*E*)-3-(2-(5-bromo-2-hydroxyphenyl)-5-methylbenzo[d]oxazol-3(2H)-yl)acrylate (3f)

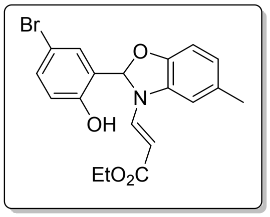

Yield : 90%; white solid; M. P : 190-191°C; Reaction time : 3 h; ^1H NMR (400 MHz, $CDCl_3$) : δ 1.14 (d, J = 6.8Hz, 3H), 2.26 (s, 3H), 4.00 (q, J = 4Hz, 2H), 4.64 (d, J = 13.6Hz, 1H), 6.71 – 7.43 (m, 7H), 7.86 (d, J = 13.6Hz, 1H), 10.62 (s, 1H) ; ^{13}C NMR (100 MHz, $CDCl_3$) : δ 14.44, 20.79, 59.08, 90.11, 92.14, 108.34, 109.48, 110.36, 118.53, 123.14, 123.71, 129.35, 131.00, 131.71, 134.07, 138.12, 147.50, 155.05, 166.90; IR (KBr) : 1587, 1642, 2991, 3484cm^{-1}; HRMS calculated for $C_{19}H_{18}BrNO_4$ $[M+H]^+$ 404.0495, found 404.0493

Ethyl (*E*)-3-(2-(5-bromo-2-hydroxyphenyl)-5-chlorobenzo[d]oxazol-3(2H)-yl)acrylate (3g)

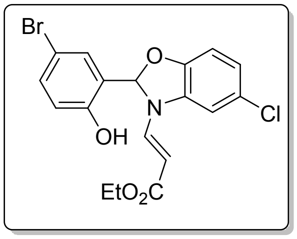

Yield : 86%; white solid; M. P : 189-190°C; Reaction time : 2 h; ^{1}H NMR (400 MHz, $CDCl_3$) : δ 1.16 (t, J = 6.8 Hz, 3H), 4.00 (q, J = 1.8Hz, 2H), 4.68 (d, J=13.6 Hz, 1H), 6.71-7.33 (m, 7H), 7.83 (d, J = 13.6Hz, 1H), 10.47 (s, 1H); ^{13}C NMR (75 MHz, $CDCl_3$) : δ 14.19, 58.99, 90.93, 93.79, 108.58, 109.12, 110.42, 118.16, 121.88, 122.93, 125.57, 129.28, 133.27, 133.83, 137.58, 148.34, 154.79, 166.50; IR (KBr) : 1603, 1634, 2987, 3411cm^{-1}; HRMS calculated for $C_{18}H_{15}BrClNO_4$ $[M+H]^+$ 423.9949, found 423.9946.

Ethyl (*E*)-3-(2-(5-bromo-2-hydroxyphenyl)-5-nitrobenzo[d]oxazol-3(2H)-yl)acrylate (3h)

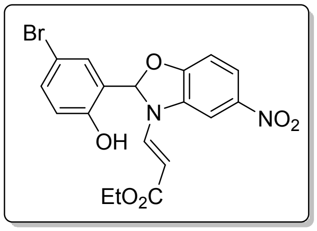

Yield : 84%; Pale yellow solid; M. P : 155-156°C; Reaction time : 4 h; ^{1}H NMR (400 MHz, $CDCl_3$) : δ 1.29 (t, J=7.2 Hz, 3H), 4.22 (q, J = 7.2Hz, 2H), 5.70 (d, J=12.4 Hz, 1H), 7.06-8.32 (m, 8H), 9.01 (s, 1H); ^{13}C NMR (100 MHz, $CDCl_3$) : δ 14.41, 60.71, 105.14, 112.53, 115.60, 118.75, 120.12, 125.49, 127.30, 130.94, 135.12, 136.87, 141.41, 153.15, 155.13, 157.54, 158.09, 166.50; IR (KBr) : 1596, 1698, 2831, 3437cm^{-1}; HRMS calculated for $C_{18}H_{15}BrN_2O_6$ $[M+H]^+$ 435.0189, found 435.0179.

Methyl (*E*)-3-(2-(5-chloro-2-hydroxyphenyl)-5 methylbenzo[d]oxazol-3(2H)-yl)acrylate (3i)

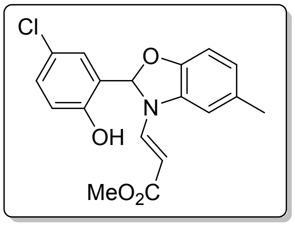

Yield : 80%; white solid; M. P : 204-205°C; Reaction time : 3 h; ^{1}H NMR (400 MHz, $CDCl_3$) : δ 2.27 (s, 3H), 3.55 (s, 3H), 4.65 (d, J = 12Hz, 1H) 6.69-7.32 (m, 7H), 7.87 (d, J = 12Hz, 1H), 10.59 (s, 1H); ^{13}C NMR (100 MHz, $CDCl_3$) : δ 20.76, 50.65, 90.16, 91.80, 108.32, 109.51, 118.02, 122.89, 123.13, 126.53, 130.98, 131.18, 131.66, 138.15, 147.52, 154.60, 167.25; IR (KBr) : 1611, 1663, 3228, 3443cm^{-1}; HRMS calculated for $C_{18}H_{16}ClNO_4$ $[M+H]^+$ 346.0844, found 346.0843.

Methyl (E)-3-(5-chloro-2-(5-chloro-2-hydroxyphenyl)benzo[d]oxazol-3(2H)-yl)acrylate (3j)

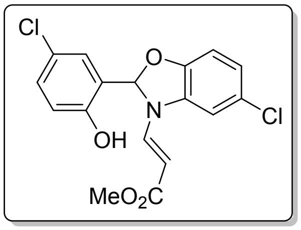

Yield : 75%; white solid; M. P : 201-202°C; Reaction time : 2 h; ^{1}H NMR (400 MHz, $CDCl_3$) : δ 3.55 (s, 3H), 4.67 (d, J = 13.6Hz, 1H), 6.83 – 7.53 (m, 7H), 7.95 (d, J = 13.6Hz, 1H), 10.58 (s, 1H); ^{13}C NMR (100 MHz, $CDCl_3$) : δ 49.16, 89.54, 92.27, 106.86, 107.77, 116.19, 120.59, 120.86, 122.16, 124.42, 124.90, 129.51, 131.73, 135.92, 146.98, 152.77, 165.78; IR (KBr) : 1601, 1665, 2831, 3427cm^{-1}; **HRMS** calculated for $C_{17}H_{13}Cl_2NO_4$ $[M+H]^+$ 366.0298, found 366.0299.

Methyl (*E*)-3-(2-(5-chloro-2-hydroxyphenyl)-5-nitrobenzo[d]oxazol-3(2H)-yl)acrylate (3k)

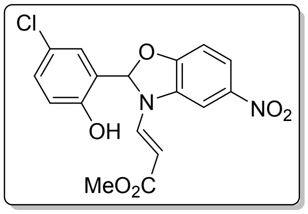

Yield : 73%; Pale yellow solid; M. P : 155-156°C; Reaction time : 3.5 h; ^{1}H NMR (400 MHz, $CDCl_3$) : δ 3.59 (s, 3H), 4.83 (d, J = 13.6Hz, 1H), 6.77 – 7.82 (m, 7H), 10.08 (s, 1H); ^{13}C NMR (100 MHz, $CDCl_3$) : δ 50.54, 92.42, 95.07, 102.52, 107.58, 117.38, 119.97, 121.01, 123.66, 126.16, 131.07, 133.03, 136.46, 142.07, 144.04, 153.97, 154.66, 166.86; IR (KBr) : 1651, 1697, 3073, 3425 cm^{-1}; HRMS calculated for $C_{17}H_{13}ClN_2O_6$ $[M+H]^+$ 377.0538, found 377.0525.

Ethyl (*E*)-3-(2-(5-chloro-2-hydroxyphenyl)-5-methylbenzo[d]oxazol-3(2H)-yl)acrylate (3l)

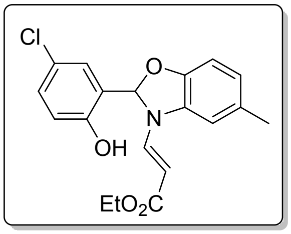

Yield : 89%; white solid; M. P : 186-187°C; Reaction time : 4 h; ^{1}H NMR (400 MHz, $CDCl_3$) : *δ* 1.14 (t, *J* = 8Hz, 3H), 2.27 (s, 3H), 4.01 (q, J = 7.2Hz, 2H), 4.63 (d, *J* = 12Hz, 1H), 6.71 – 7.32 (m, 7H), 7.86 (d, J = 12Hz, 1H), 10.59 (s, 1H) ; ^{13}C NMR (100 MHz, $CDCl_3$) : δ 14.45, 20.79, 59.08, 90.17, 92.11, 108.35, 109.50, 118.05, 122.91, 123.17, 126.52, 131.00, 131.21, 131.71, 138.12, 147.50, 154.62, 166.90; IR (KBr) : 1620, 1670, 3181, 3448cm^{-1}; HRMS calculated for $C_{19}H_{18}ClNO_4$ $[M+H]^+$ 360.1000, found 360.0997.

Methyl (*E*)-3-(2-(3-ethoxy-2-hydroxyphenyl)-5-methylbenzo[d]oxazol-3(2H)-yl)acrylate (3m)

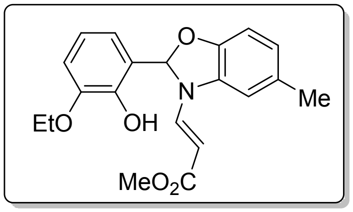

Yield : 84%; M. P : 159-160°C; Reaction time : 4 h; ^{1}H NMR (400 MHz, $CDCl_3$) : *δ* 1.45 (t, *J*=7.2Hz, 3H), 2.33 (s, 3H), 3.66 (s, 3H), 4.11 (q, J= 6.8Hz, 2H), 4.80 (d, *J*=13.6 Hz, 1H), 6.09 (s, 1H), 6.66 – 6.88 (m, 6H), 7.12 (s, 1H), 7.86 (d, *J* = 13.6, 1H) ; ^{13}C NMR (75 MHz, $CDCl_3$) : δ 14.98, 21.36, 51.13, 64.91, 91.13, 92.58, 108.84, 112.94, 118.80, 120.39, 120.88, 123.31, 131.35, 132.25, 138.27, 144.22, 146.13, 148.26, 168.75; IR (KBr) : 1620, 3031, 3243, 3434cm^{-1}; HRMS calculated for $C_{25}H_{22}N_3O_2$ $[M+H]^+$ 356.1496, found 356.1496.

Methyl (*E*)-3-(5-chloro-2-(5-chloro-2-hydroxyphenyl)benzo[d]oxazol-3(2H)-yl)acrylate (3n)

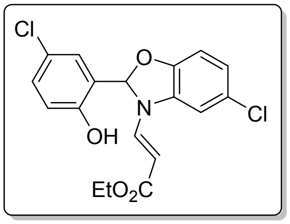

Yield : 80%; white solid; M. P : 171-172°C; Reaction time : 2 h; ^{1}H NMR (400 MHz, $CDCl_3$) : δ 1.14 (t, J = 7.2Hz, 3H), 4.02 (q, J = 7.2Hz, 2H), 4.65 (d, J = 13.6Hz, 1H), 6.82 – 7.52 (m, 7H), 7.93 (d, J = 13.6Hz, 1H), 10.60 (s, 1H) ; ^{13}C NMR (100 MHz, $CDCl_3$) : δ14.41, 59.19, 91.48, 93.62, 109.15, 109.43, 118.07, 122.12, 122.67, 122.93, 125.42, 127.00, 131.43, 133.60, 138.19, 148.62, 154.76, 166.63 ; IR (KBr) : 1631, 1676, 2990, 3456cm^{-1} ; HRMS calculated for $C_{18}H_{15}Cl_2NO_4$ $[M+H]^+$ 380.0454, found 380.0455.

Methyl (*E*)-3-(2-(5-bromo-2-hydroxyphenyl)benzo[d]oxazol-3(2H)-yl)acrylate (3o)

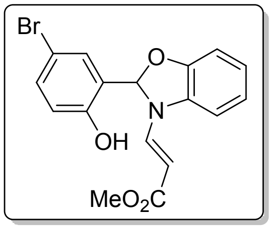

Yield : 86%; white solid; M. P : 188-189°C; Reaction time: 3 h; ^{1}H NMR (400 MHz, $CDCl_3$) : δ 3.64 (s, 3H), 4.79 (d, J = 13.6 Hz, 1H), 6.78 – 7.72 (m, 8H), 7.86 (d, J = 13.6Hz, 1H), 10.21 (s, 1H) ; ^{13}C NMR (100 MHz, $CDCl_3$) : δ 50.75, 89.96, 92.66, 107.71, 109.02, 111.27, 118.03, 121.36, 122.99, 123.14, 129.28, 131.79, 133.75, 137.90, 149.64, 154.58, 168.12 ; IR (KBr) : 1600, 1656, 2946, 3437 cm^{-1}; HRMS calculated for $C_{17}H_{14}BrNO_4$ $[M+H]^+$ 376.0182, found 376.0171.

(Ethyl (*E*)-3-(2-(5-bromo-2-hydroxyphenyl)benzo[d]oxazol-3(2H)-yl)acrylate (3p)

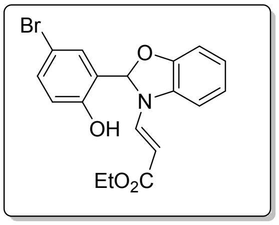

Yield : 88%; white solid; M. P : 170-171 °C; Reaction time : 4 h; ^{1}H NMR (400 MHz, $CDCl_3$) : δ 1.23 (t, J = 7.2Hz, 3H), 4.09 (q, J = 6.8Hz, 2H), 4.79 (d, J = 13.6Hz, 1H), 6.78 – 7.54 (m, 6H), 7.86 (d, J = 13.6Hz, 1H), 10.05 (s, 1H); ^{13}C NMR (75 MHz, $CDCl_3$) : δ 13.93, 58.99, 89.59, 92.68, 107.40, 108.59, 110.76, 117.73, 121.03, 122.59, 122.89, 128.84, 131.47, 133.36, 137.43,

149.21, 154.32, 167.24; IR (KBr) : 1621, 1667, 3179, 3433cm^{-1}, HRMS calculated for $C_{18}H_{16}BrNO_4$ $[M+H]^+$ 390.0339, found 390.0338.

Methyl (*E*)-3-(2-(5-chloro-2-hydroxyphenyl)benzo[d]oxazol-3(2H)-yl)acrylate (3q)

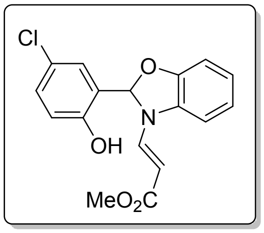

Yield : 79%; white solid; M. P : 191-192°C; Reaction time : 3 h; ^{1}H NMR (400 MHz, $CDCl_3$) : δ 3.55 (s, 3H), 4.66 (d, J = 13.6Hz, 1H), 6.85-7.36 (m, 8H), 7.91 (d, J = 13.6Hz, 1H), 10.59 (s, 1H); ^{13}C NMR (100 MHz, $CDCl_3$) : δ 50.38, 89.62, 92.25, 107.49, 108.62, 117.33, 121.14, 122.43, 122.71, 123.62, 126.02, 130.54, 131.45, 137.52, 149.31, 153.90, 167.60; IR (KBr) : 1600, 1665, 3224, 3452 cm^{-1}; HRMS calculated for $C_{17}H_{14}ClNO_4$ $[M+H]^+$ 332.0687, found 332.0684.

Ethyl *(E)*-3-(2-(5-bromo-2-hydroxyphenyl)benzo*[d]*thiazol-3*(2H)*-yl)acrylate (3r)

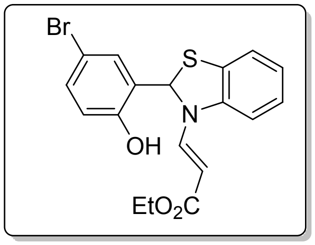

Yield : 65 %; M. P : 161-162°C; Reaction time : 4 h; ^{1}H NMR (400 MHz, $CDCl_3$) : δ 1.29 (t, J = 6.8Hz, 3H), 4.20 (q, J = 7.2 Hz, 2H), 5.80 (d, J = 12.4 Hz, 1H); 7.08 – 8.12 (m, 7H), 8.69 (s, 1H); ^{13}C NMR (100 MHz, $CDCl_3$) : δ 14.41, 60.60, 104.73, 118.82, 120.31, 121.56, 123.53, 125.76, 126.64, 132.69, 134.65, 136.23, 152.22, 152.41, 157.48, 160.02, 166.59; IR (KBr) : 1650, 1693, 2832, 3425 cm^{-1}; HRMS calculated for $C_{18}H_{16}BrNO_3S$ $[M-H]^+$ 403.9958, found 403.9954.

Ethyl *(E)*-3-(2-(5-bromo-2-hydroxyphenyl)benzo*[d]*thiazol-3*(2H)*-yl)acrylate (3s)

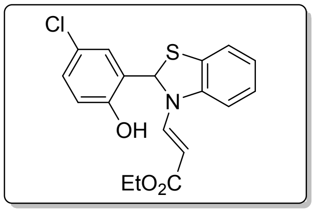

Yield : 68 %; M. P : 163-164°C; Reaction time : 4 h; ^{1}H NMR (400 MHz, $CDCl_3$) : δ 1.29 (t, J =8 Hz, 3H), 4.19 (q, J = 4Hz, 2H), 5.80 (d, J = 12Hz, 1H), 7.15-8.55 (m,7H), 8.56 (s, 1H); ^{13}C NMR (75 MHz, $CDCl_3$) : δ 14.42, 60.60, 104.62, 120.09, 121.58, 123.53, 125.77, 126.03, 126.65, 129.77, 131.39, 131.73, 136.23, 151.89, 152.23, 157.66, 160.16, 166.63 ; IR (KBr) : 1615, 1680, 2945, 3437 cm^{-1}; HRMS calculated for $C_{18}H_{16}NO_3S$ $[M-H]^+$ 360.0463, found 360.0459.

Ethyl (*E*)-3-(2-(2-hydroxyphenyl)-2H-benzo*[d]*[1,3]oxazin-1*(4H)*-yl)acrylate (5a)

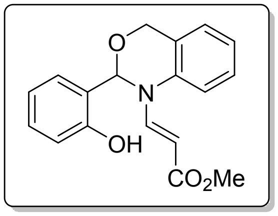

Yield : 92%; M. P : 202-203°C; Reaction time : 6 h; ^{1}H NMR (400 MHz, $CDCl_3$) : δ 3.53 (s, 3H), 4.58 (d, J = 14Hz, 1H), 4.68 (dd, J = 14Hz, 8.8Hz, 2H), 6.63 (s, 1H), 6.71-7.40 (m, 8H), 7.93 (d, J = 14Hz, 1H), 10.04 (s, 1H); ^{13}C NMR (100 MHz, $CDCl_3$) : δ 50.50, 62.31, 81.49, 92.18, 114.25, 116.07, 118.82, 120.84, 122.02, 124.27, 124.84, 127.00, 128.21, 130.13, 137.53, 141.62, 155.32, 168.52; IR (KBr) : 1605, 1663, 3179, 3433cm^{-1}; HRMS calculated for $C_{18}H_{17}NO_4$ $[M+H]^+$ 312.1234, found 312.1214.

Methyl (*E*)-3-(2-(5-bromo-2-hydroxyphenyl)-2H-benzo*[d]*[1,3]oxazin-1*(4H)*-yl)acrylate (5b)

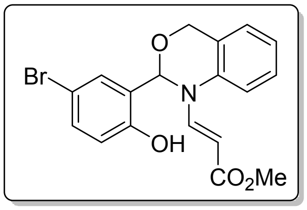

Yield : 85%; M. P : 204-205°C; Reaction time : 6 h; ^{1}H NMR (400 MHz, $CDCl_3$) : δ 3.65 (s, 3H), 4.65 (s, 2H), 4.83 (d, J = 13.6Hz, 1H), 6.49 (s, 1H), 6.88 – 7.56 (m, 7H), 8.06 (d, J = 13.6Hz, 1H), 9.80 (s, 1H) ; ^{13}C NMR (100 MHz, $CDCl_3$) : δ 50.34, 62.70, 80.92, 92.18, 110.33, 114.47, 117.77, 122.12, 123.43, 124.55, 124.75, 128.29, 129.24, 132.61, 137.32, 141.50, 154.49, 168.10 ; IR (KBr) : 1610, 1673, 3231, 3444 cm^{-1}; HRMS calculated for $C_{18}H_{16}BrNO_4$ $[M+H]^+$ 390.0339, found 390.0320.

Ethyl (*E*)-3-(2-(5-bromo-2-hydroxyphenyl)-2H-benzo*[d]*[1,3]oxazin-1*(4H)*-yl)acrylate (5c)

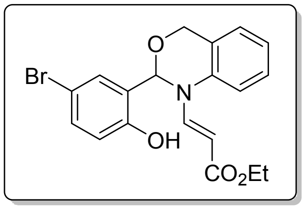

Yield : 90%; M. P : 200-201°C; Reaction time : 5 h; ^{1}H NMR (400 MHz, $CDCl_3$) : δ 1.27 (t, J = 7.2 Hz, 3H), 4.18 (q, J =7.2 Hz, 2H), 4.70 (dd, 14.8Hz, 14.4Hz, 2H), 5.03 (d, J = 13.6 Hz, 1H), 6.47 (s, 1H), 6.83-7.35 (m, 7H), 8.15 (d, J = 13.6Hz, 1H); ^{13}C NMR (100 MHz, $CDCl_3$) : δ 13.99, 58.97, 62.64, 80.98, 92.66, 110.41, 114.50, 117.84, 122.11, 123.44, 124.44, 124.77, 128.29, 129.33, 132.65, 137.35, 141.45, 154.55, 167.79 ; IR (KBr) : 1606, 1668, 3240, 3432cm^{-1}; HRMS calculated for $C_{19}H_{18}BrNO_4$ $[M+H]^+$ 404.0492, found 404.0496.

Methyl (*E*)-3-(2-(5-chloro-2-hydroxyphenyl)-2H-benzo*[d]*[1,3]oxazin-1*(4H)*-yl)acrylate (5d)

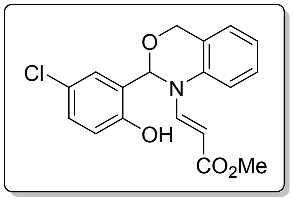

Yield : 84%; M. P : 201-202°C; Reaction time : 6 h; ^{1}H NMR (400 MHz, $CDCl_3$) : δ 3.55 (s, 3H), 4.57 (s, 2H), 4.72 (d, J = 12 Hz, 1H), 6.39 (s, 1H), 6.71 – 7.69 (m, 7H), 7.95 (d, J = 12Hz, 1H), 9.98 (s, 1H) ; ^{13}C NMR (100 MHz, $CDCl_3$) : δ 49.77, 62.16, 80.35, 91.48, 113.99, 116.77, 121.66, 122.33, 122.54, 124.23, 124.28, 125.76, 127.83, 129.14, 136.86, 140.95, 153.53, 167.39; IR (KBr) : 1611, 1673, 3237, 3439cm^{-1}; HRMS calculated for $C_{18}H_{16}ClNO_4$ $[M+H]^+$ 346.0833, found 346.0838.

Methyl (*E*)-3-(2-(3-ethoxy-2-hydroxyphenyl)-2H-benzo*[d]*[1,3]oxazin-1*(4H)*-yl)acrylate (5e)

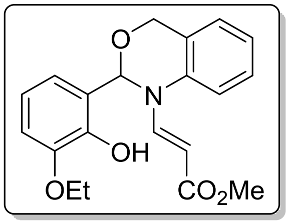

Yield : 87%; M. P : 203-204°C; Reaction time : 5 h; ^{1}H NMR (400 MHz, $CDCl_3$) : *δ* 1.46 (t, *J*=7.2 Hz, 3H), 3.66 (s, 3H), 4.10 (q, *J*=6.8 Hz, 2H), 4.66 (s, 2H), 4.89 (d, *J* = 13.6Hz, 1H), 6.16 (s, 1H), 6.53 – 7.03 (m, 8H), 8.14 (d, *J* = 13.6 Hz, 1H); ^{13}C NMR (75 MHz, $CDCl_3$) : δ 14.99, 31.04, 51.11, 63.03, 64.89, 81.85, 92.97, 112.64, 114.91, 119.39, 119.71, 121.14, 122.53, 124.73, 125.30, 128.75, 138.01, 142.19, 144.33, 146.34, 169.15; IR (KBr) : 1631, 3045, 3240, 3429cm^{-1}; HRMS calculated for $C_{25}H_{21}ClN_3O_2$ $[M+Na]^+$ 378.1317, found 378.1324

Dimethyl 2-(2-(2-((4-methylphenyl)sulfonamido)phenyl)benzo*[d]*oxazol-3*(2H)*-yl)fumarate (7a)

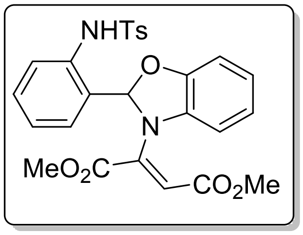

Yield : 72%; yellow solid, M. P : 159-160°C; Reaction time : 8 h; ^{1}H NMR (400 MHz, $CDCl_3$) : *δ* 2.19 (s, 3H), 3.62 (s, 3H), 4.01 (s, 3H), 5.02 (s, 1H), 6.77 – 7.58 (m, 12H), 8.19 (d, *J* = 1.6 Hz, 1H), 8.20 (s, 1H); ^{13}C NMR (75 MHz, $CDCl_3$) : δ 21.57, 51.94, 53.68, 105.71, 115.11, 116.62, 120.29, 128.49, 128.60, 129.86, 130.76, 132.23, 132.45, 133.75, 134.51, 134.59, 136.60, 145.98, 147.95, 150.90, 152.79, 164.45, 166.45 ; IR (KBr) : 1765, 1790, 3251, 3445cm^{-1}; HRMS calculated for $C_{26}H_{24}N_2O_7S$ $[M+H]^+$ 509.1380, found 509.1361.

Methyl *(E)*-3-(2-(3-ethoxy-2-hydroxyphenyl)-2H-benzo*[d]*[1,3]oxazin-1*(4H)*-yl)acrylate (7b)

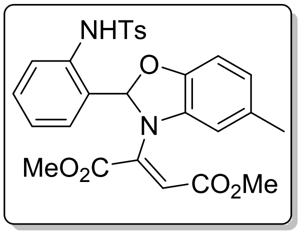

Yield : 78%; Pink solid, M. P : 171-172°C; Reaction time : 8 h; ^{1}H NMR (400 MHz, $CDCl_3$) : δ 2.22 (s, 3H), 2.34 (s, 3H), 3.62 (s, 3H), 4.01 (s, 3H), 5.02 (s, 1H), 6.85 – 8.50 (m, 10 H), 8.79 (s, 1H); ^{13}C NMR (75 MHz, $CDCl_3$) : δ 21.01, 21.59, 51.93, 53.09, 53.62, 105.67, 114.81, 116.89, 128.53, 128.63, 129.38, 129.83, 130.50, 130.73, 132.11, 132.26, 133.92, 134.15, 134.58, 136.54, 145.93, 147.96, 150.60, 165.49; IR (KBr) : 1735, 1785, 3267, 3466 cm^{-1}; HRMS calculated for $C_{27}H_{26}N_2O_7S$ $[M+H]^+$ 523.1537, found 523.1539.

X-ray Analysis

Ethyl (E)-3-(2-(5-bromo-2-hydroxyphenyl)-5-methylbenzo[d]oxazol-3(2H)-yl)acrylate (3d)

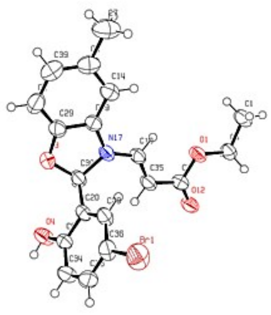

Table 1. Crystal data and structure refinement for (3d).

Identification code	EXP-134
Empirical formula	$C_{19}H_{18}BrNO_4$
Formula weight	404.25
Temperature/K	298
Crystal system	monoclinic
Space group	$P2_1/a$
a/Å	12.8540(7)
b/Å	9.8269(6)
c/Å	14.2696(11)
α/°	90
β/°	94.215(5)
γ/°	90
Volume/Å^3	1797.6(2)
Z	4

ρ_{calc}g/cm^3	1.494
μ/mm^{-1}	2.310
F(000)	824.0
Crystal size/mm^3	0.6 × 0.2 × 0.08
Radiation	MoKα (λ = 0.71073)
2Θ range for data collection/°	8.278 to 58.558
Index ranges	$-15 \le h \le 17$, $-12 \le k \le 13$, $-17 \le l \le 18$
Reflections collected	10756
Independent reflections	4183 [R_{int} = 0.0402, R_{sigma} = 0.0560]
Data/restraints/parameters	4183/0/232
Goodness-of-fit on F^2	1.017
Final R indexes [I>=2σ (I)]	R_1 = 0.0483, wR_2 = 0.1102
Final R indexes [all data]	R_1 = 0.1008, wR_2 = 0.1371
Largest diff. peak/hole / e Å^{-3}	0.41/-0.47

Methyl (E) -3-(2-(2-hydroxyphenyl)-2H-benzo[d][1,3]oxazin-1(4H)-yl)acrylate (5a)

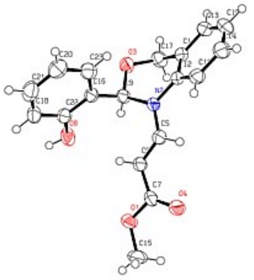

Table 2. Crystal data and structure refinement for (5a).

Identification code	MB-MM-39
Empirical formula	$C_{19}H_{18}NO_3$
Formula weight	308.34
Temperature/K	298(2)
Crystal system	orthorhombic
Space group	$P2_12_12_1$
a/Å	7.5340(5)
b/Å	13.2552(10)

c/Å	15.2014(12)
α/°	90.00
β/°	90.00
γ/°	90.00
Volume/$Å^3$	1518.09(19)
Z	4
ρ_{calc}g/cm^3	1.349
μ/mm^{-1}	0.091
F(000)	652.0
Crystal size/mm^3	0.5 × 0.04 × 0.03
Radiation	Mo Kα (λ = 0.7107)
2Θ range for data collection/°	8.62 to 58.2
Index ranges	-10 ≤ h ≤ 9, -16 ≤ k ≤ 17, -20 ≤ l ≤ 20
Reflections collected	5373
Independent reflections	3203 [R_{int} = 0.0374, R_{sigma} = 0.0614]
Data/restraints/parameters	3203/0/213
Goodness-of-fit on F^2	1.011
Final R indexes [I>=2σ (I)]	R_1 = 0.0458, wR_2 = 0.0843
Final R indexes [all data]	R_1 = 0.0758, wR_2 = 0.0976
Largest diff. peak/hole /e $Å^{-3}$	0.14/-0.18

^{1}H, ^{13}C NMR & ESI-HRMS Spectra for the Compounds 3a-s, 5a-e & 7a-b

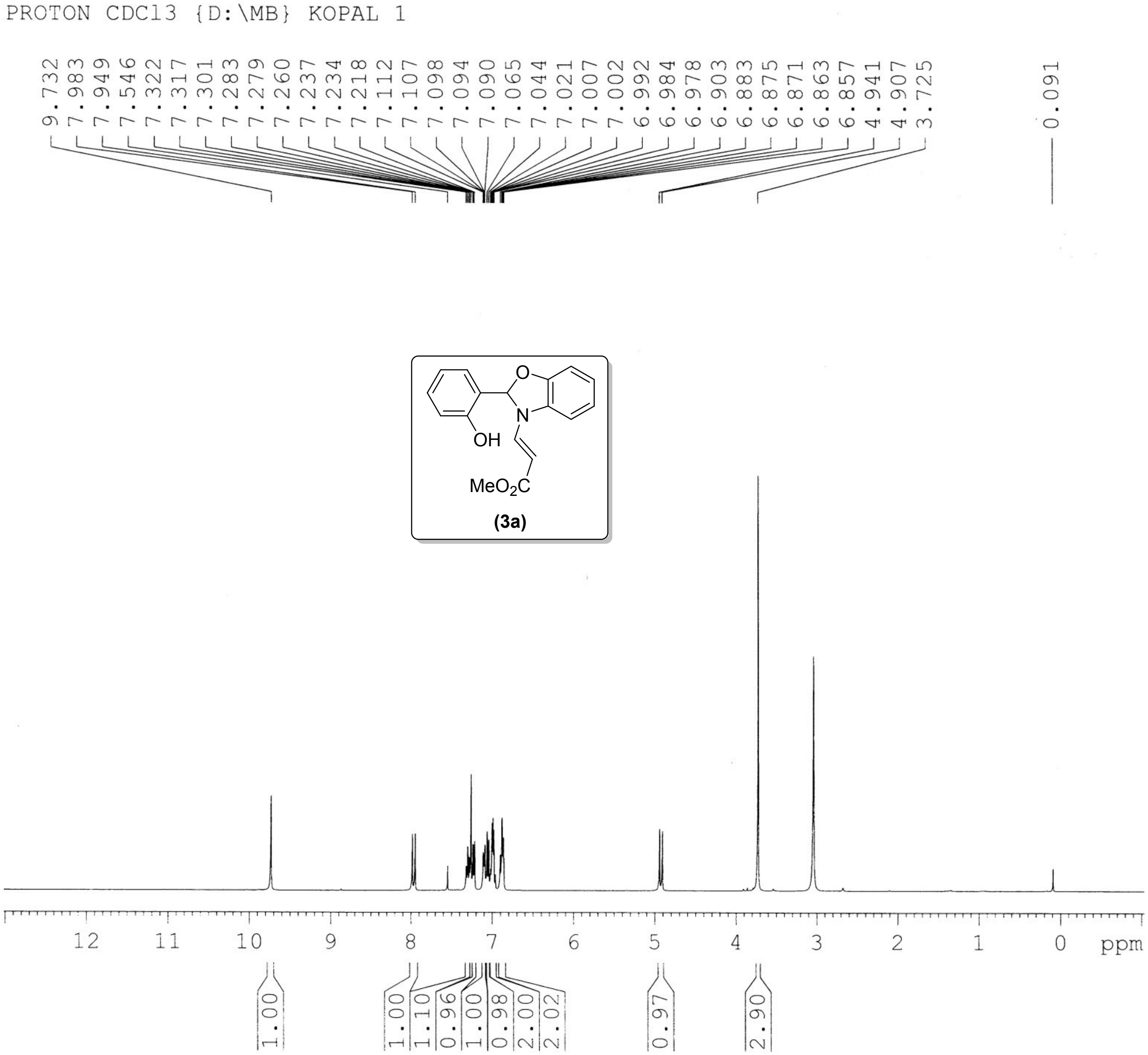

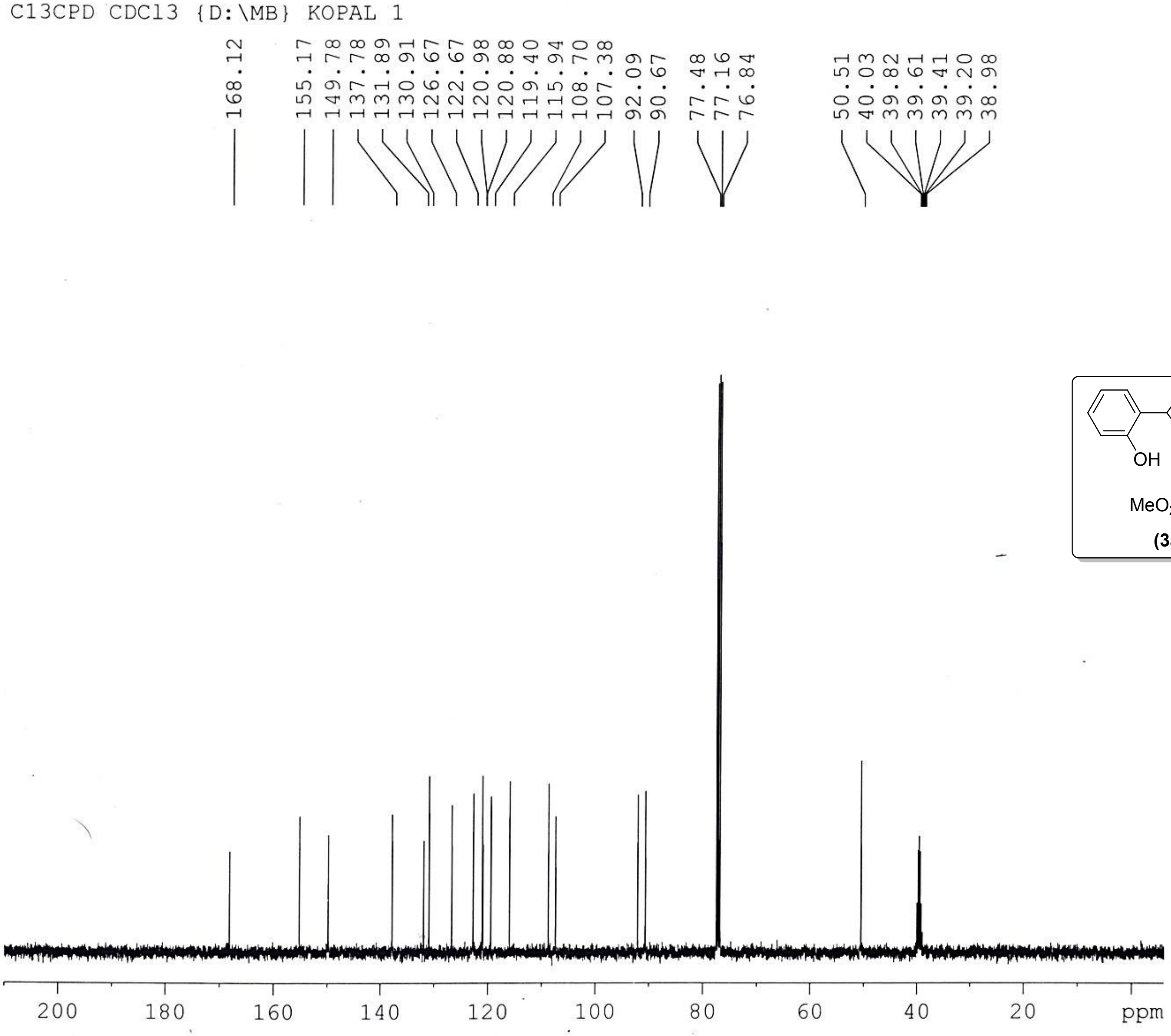
C13CPD CDCl3 {D:\MB} KOPAL 1
168.12
155.17
149.78
137.78
131.89
130.91
126.67
122.67
120.98
120.88
119.40
115.94
108.70
107.38
92.09
90.67
77.48
77.16
76.84
50.51
40.03
39.82
39.61
39.41
39.20
38.98
200
180
160
140
120
100
80
60
40
20
ppm

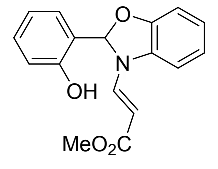
O
N
OH
MeO2C

(3a)

**Sample Name**	MM-29	**Position**		**Instrument Name**	Q-TOF	**User Name**	QTOF-PU\admin
**Inj Vol**	-1	**InjPosition**		**SampleType**	Sample	**IRM Calibration Status**	Success
**Data Filename**	MM-29.d	**ACQ Method**	Pondicherry Universi	**Comment**			

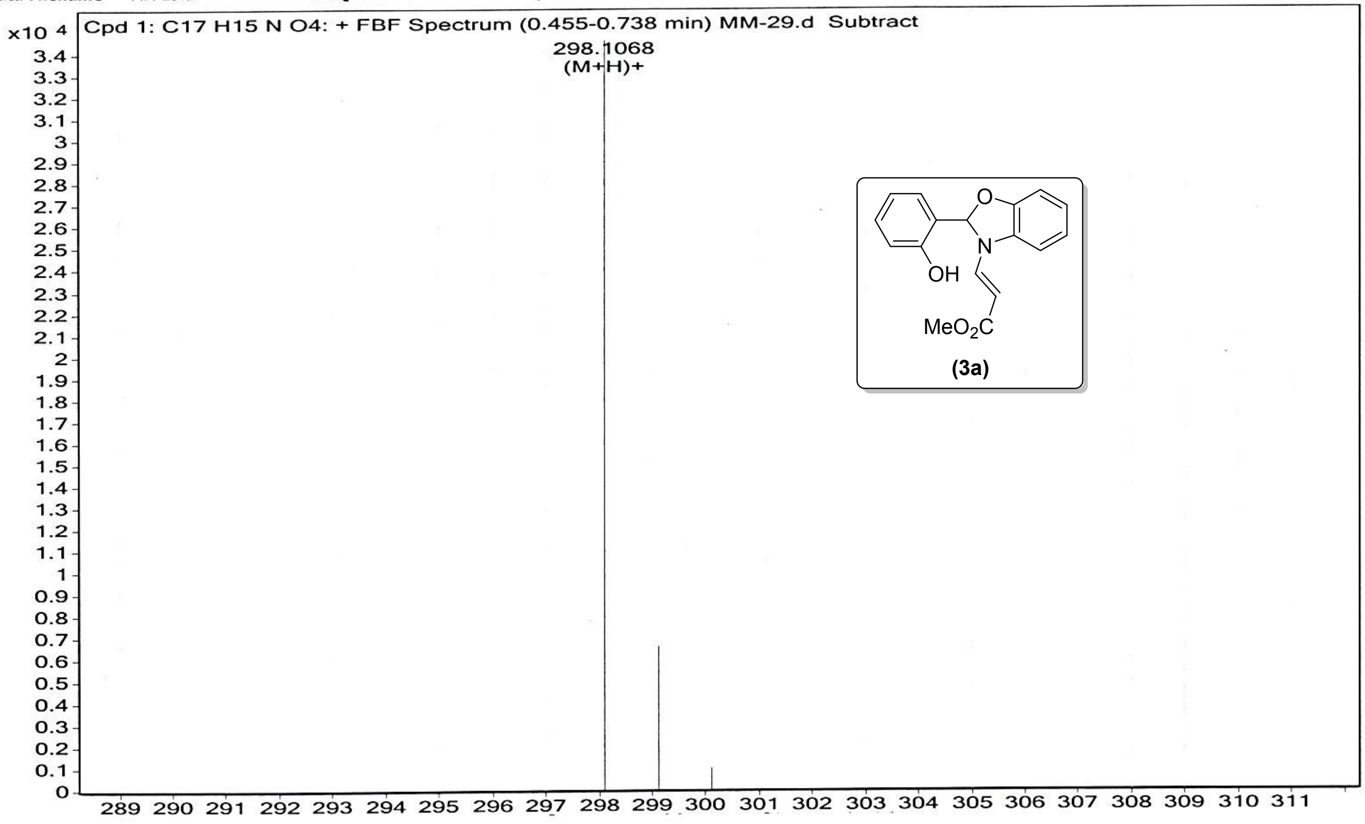

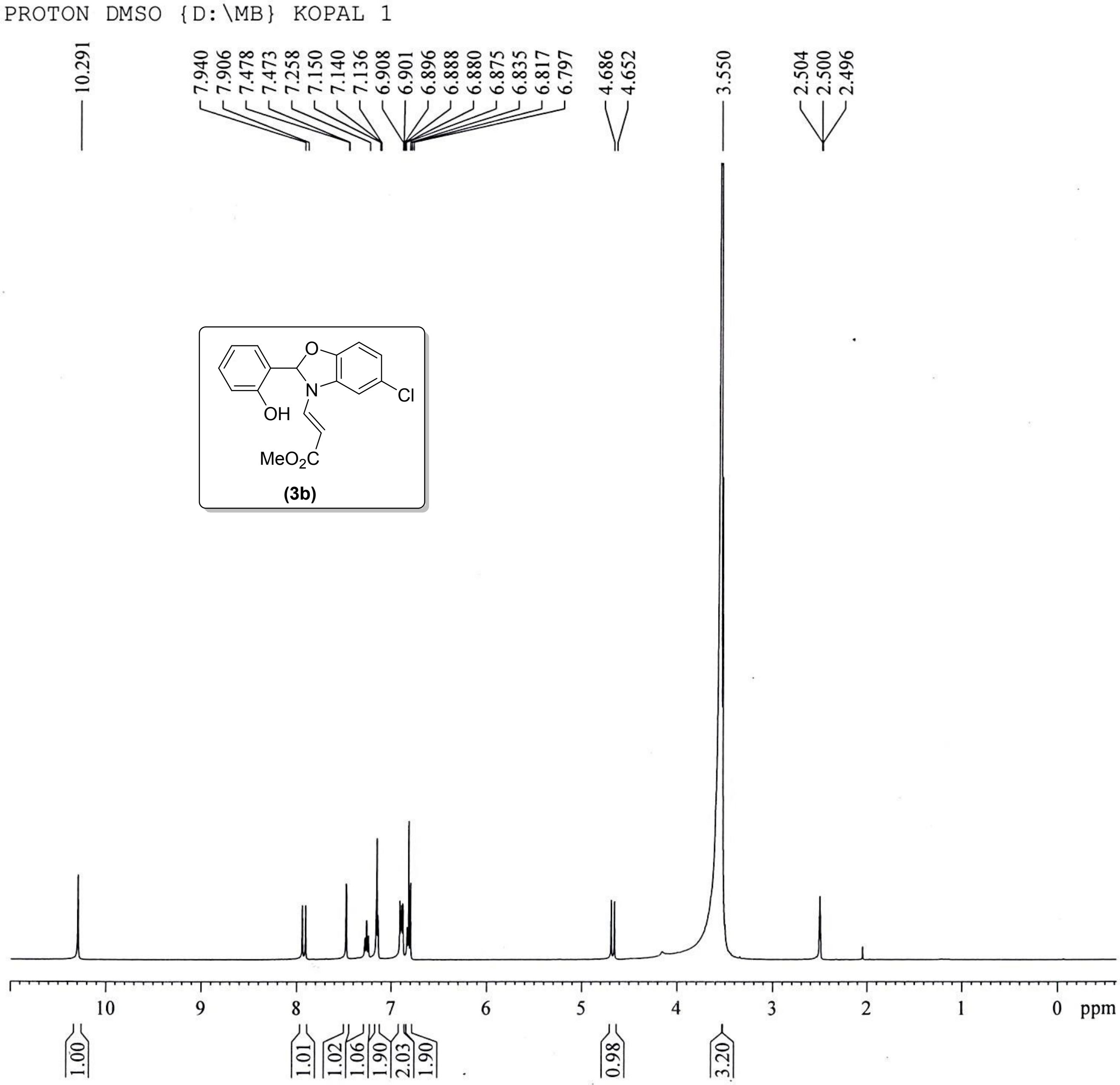
PROTON DMSO {D:\MB} KOPAL 1
10.291
7.940 7.906 7.478 7.473 7.258 7.150 7.140 7.136 6.908 6.901 6.896 6.888 6.880 6.875 6.835 6.817 6.797
4.686 4.652
3.550
2.504 2.500 2.496
O
N
Cl
OH
MeO2C
(3b)
10 9 8 7 6 5 4 3 2 1 0 ppm
1.00
1.01 1.02 1.06 1.90 2.03 1.90
0.98
3.20

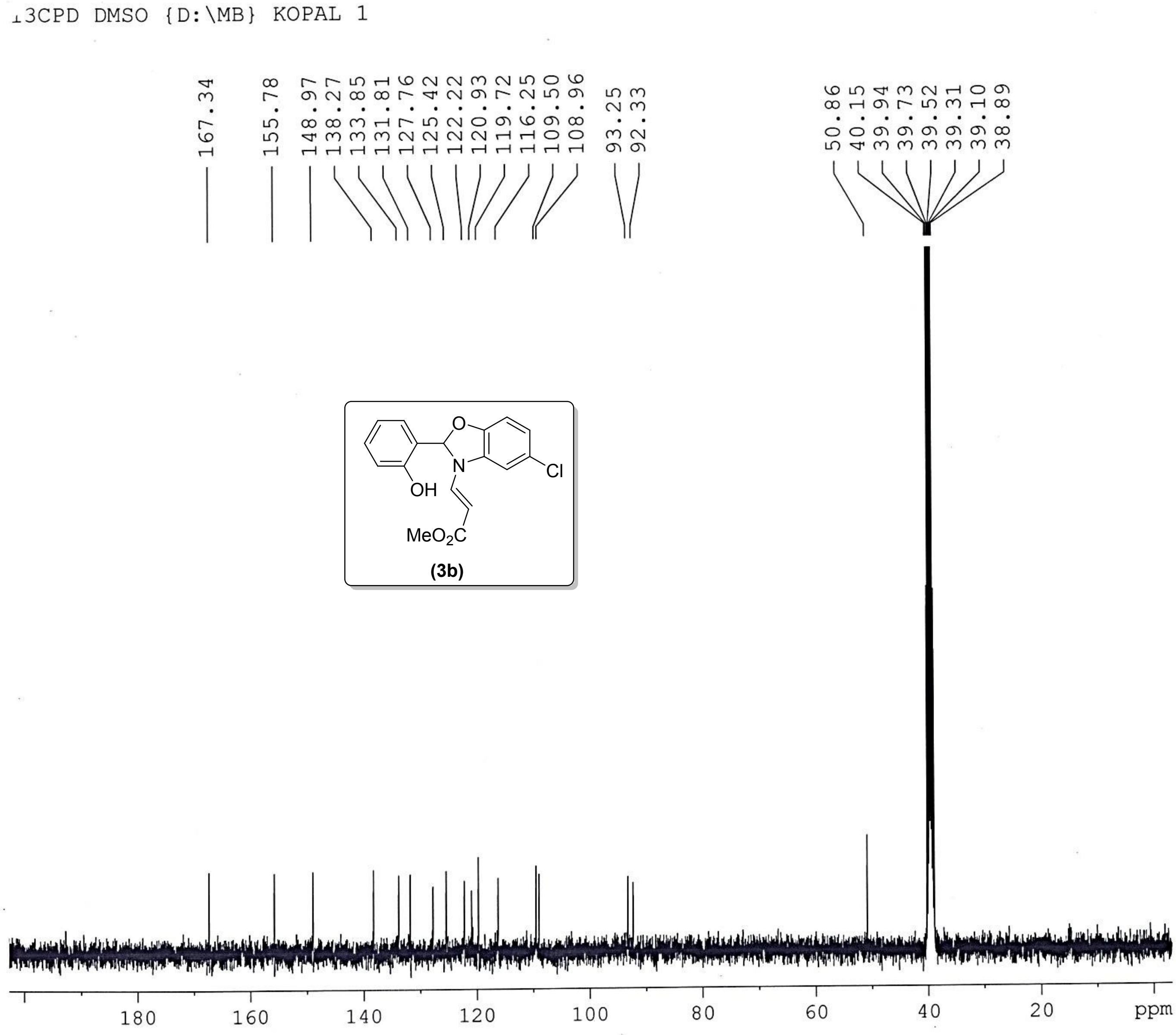
13CPD DMSO {D:\MB} KOPAL 1
167.34
155.78
148.97
138.27
133.85
131.81
127.76
125.42
122.22
120.93
119.72
116.25
109.50
108.96
93.25
92.33
50.86
40.15
39.94
39.73
39.52
39.31
39.10
38.89
OH
N
O
Cl
MeO2C
(3b)
180
160
140
120
100
80
60
40
20
ppm

**Sample Name**	MM-153	**Position**		**Instrument Name**	Q-TOF	**User Name**	QTOF-PU\admin
**Inj Vol**	-1	**InjPosition**		**SampleType**	Sample	**IRM Calibration Status**	Success
**Data Filename**	MM-153.d	**ACQ Method**	Pondicherry Universi				

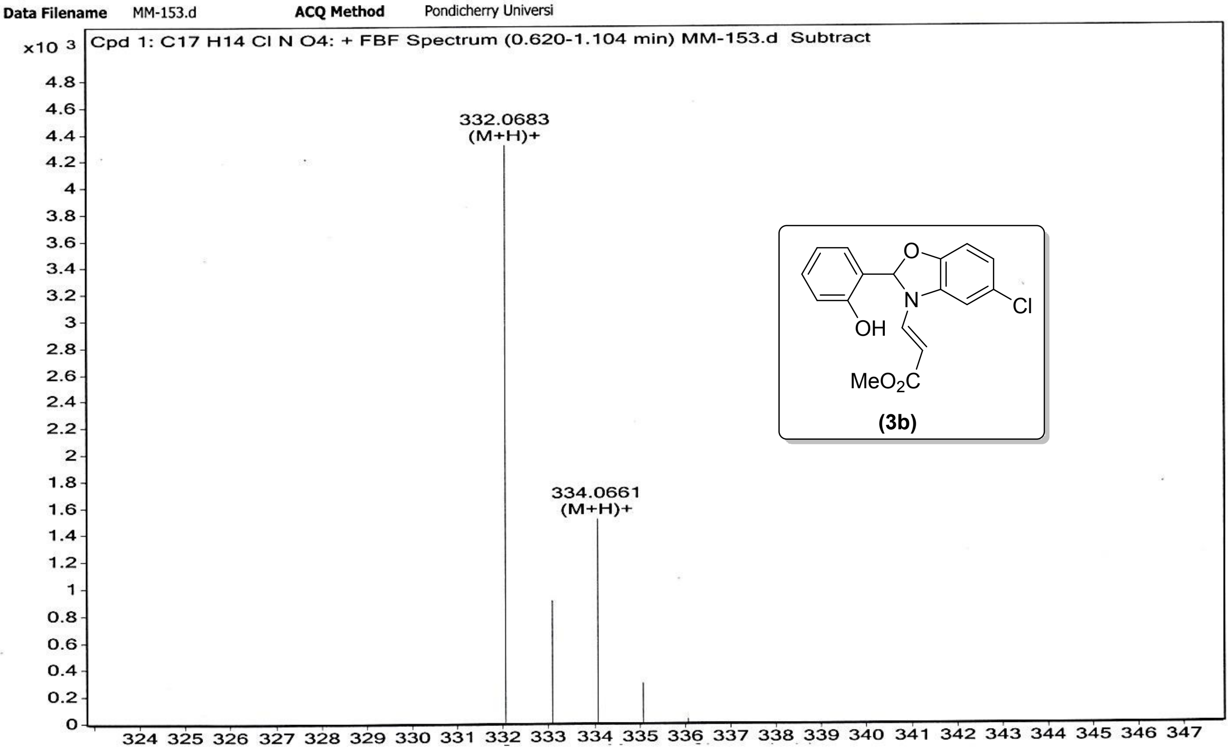

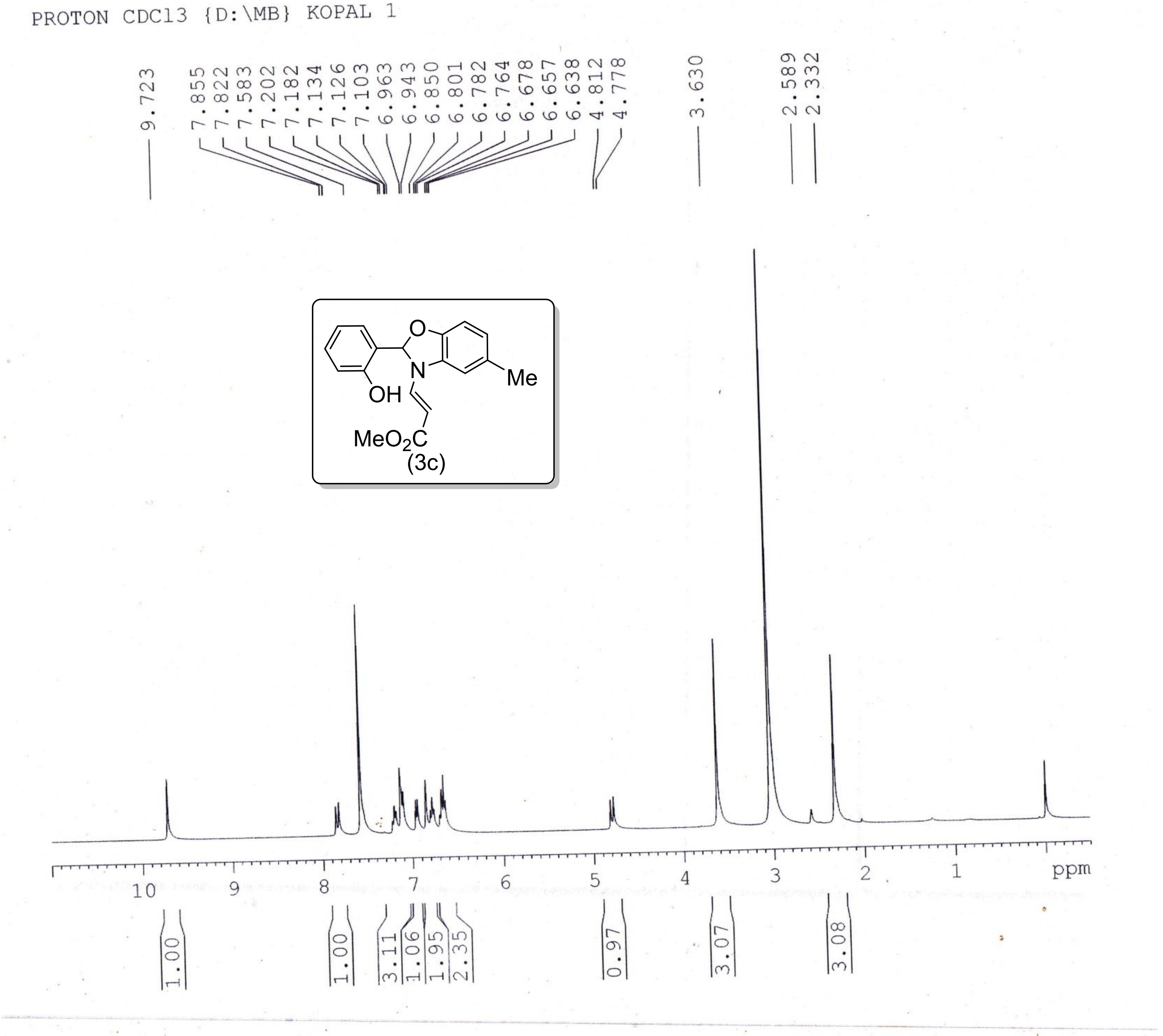
PROTON CDCl3 {D:\MB} KOPAL 1
9.723
7.855
7.822
7.583
7.202
7.182
7.134
7.126
7.103
6.963
6.943
6.850
6.801
6.782
6.764
6.678
6.657
6.638
4.812
4.778
3.630
2.589
2.332
O
N
Me
OH
MeO2C
(3c)
10 9 8 7 6 5 4 3 2 1 ppm
1.00
1.00
3.11
1.06
1.95
2.35
0.97
3.07
3.08

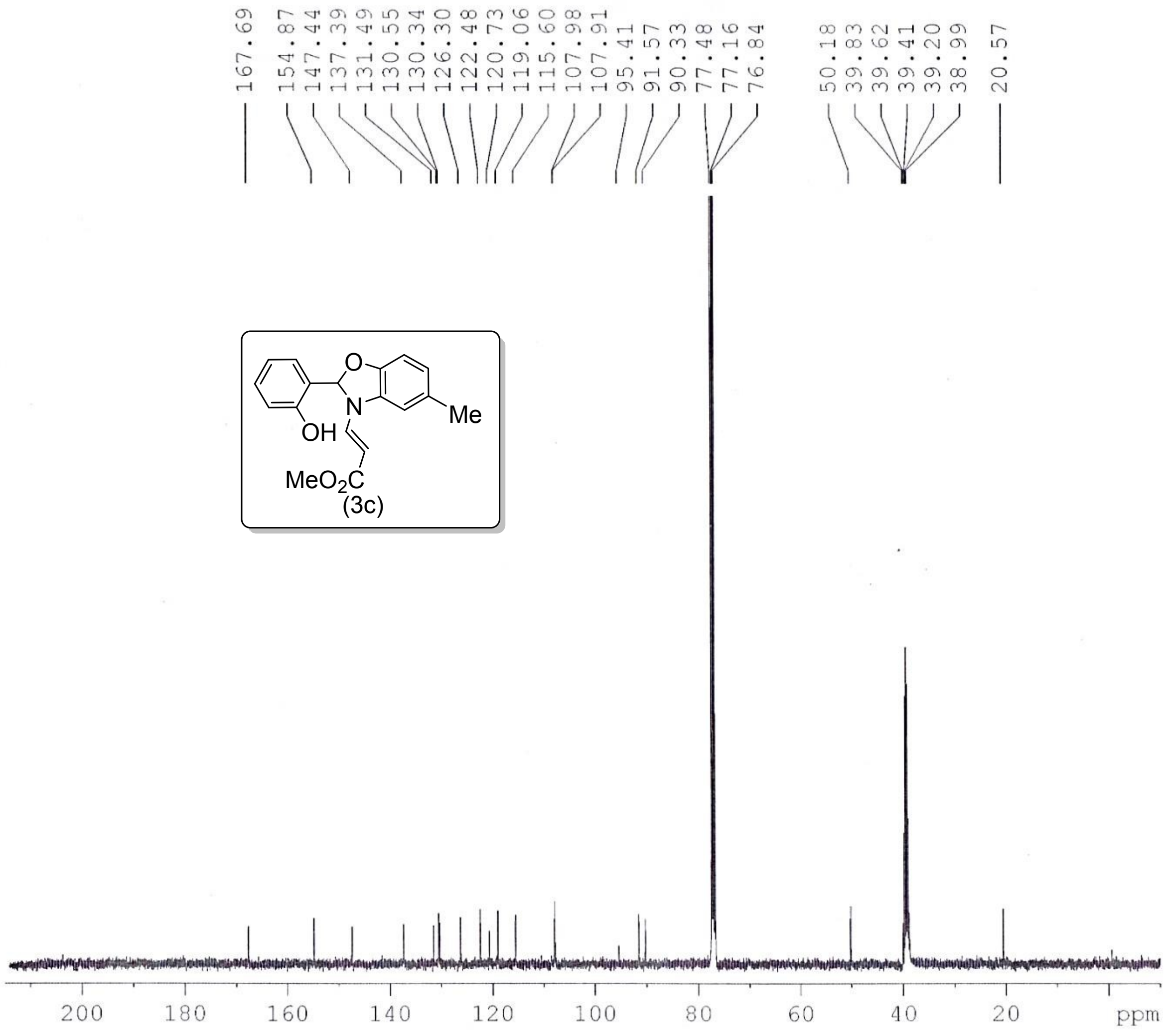
167.69
154.87
147.44
137.39
131.49
130.55
130.34
126.30
122.48
120.73
119.06
115.60
107.98
107.91
95.41
91.57
90.33
77.48
77.16
76.84
50.18
39.83
39.62
39.41
39.20
38.99
20.57
200
180
160
140
120
100
80
60
40
20
ppm
Me
OH
N
O
MeO2C
(3c)

**Sample Name**	MM-152	**Position**		**Instrument Name**	Q-TOF	**User Name**	QTOF-PU\admin
**Inj Vol**	-1	**InjPosition**		**SampleType**	Sample	**IRM Calibration Status**	Success
**Data Filename**	MM-152.d	**ACQ Method**	Pondicherry Universi				

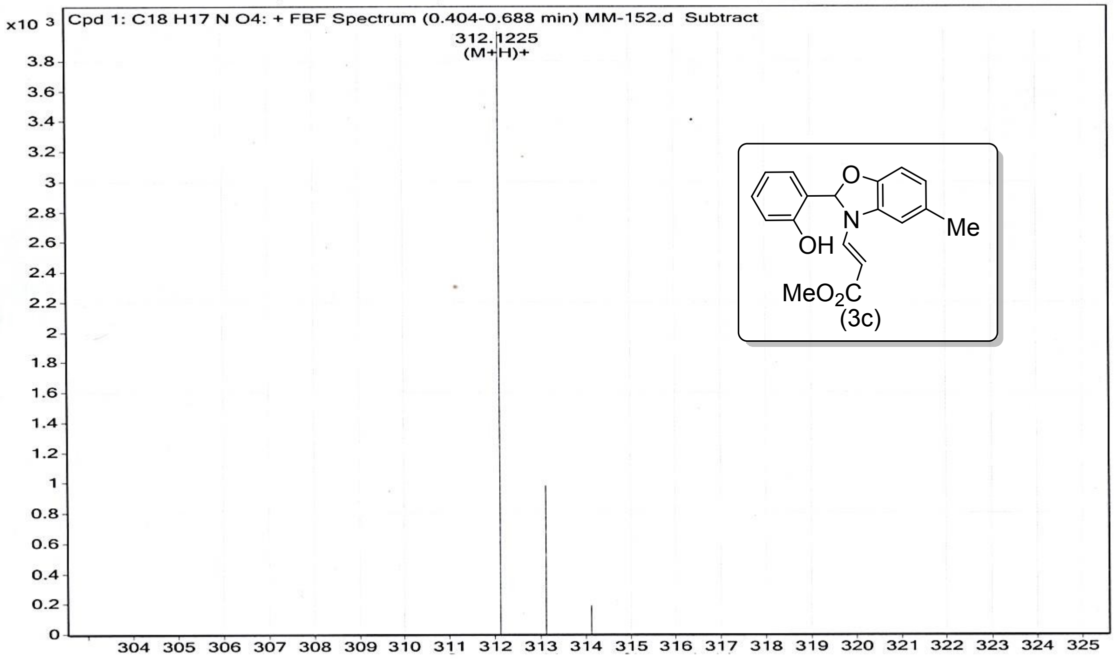

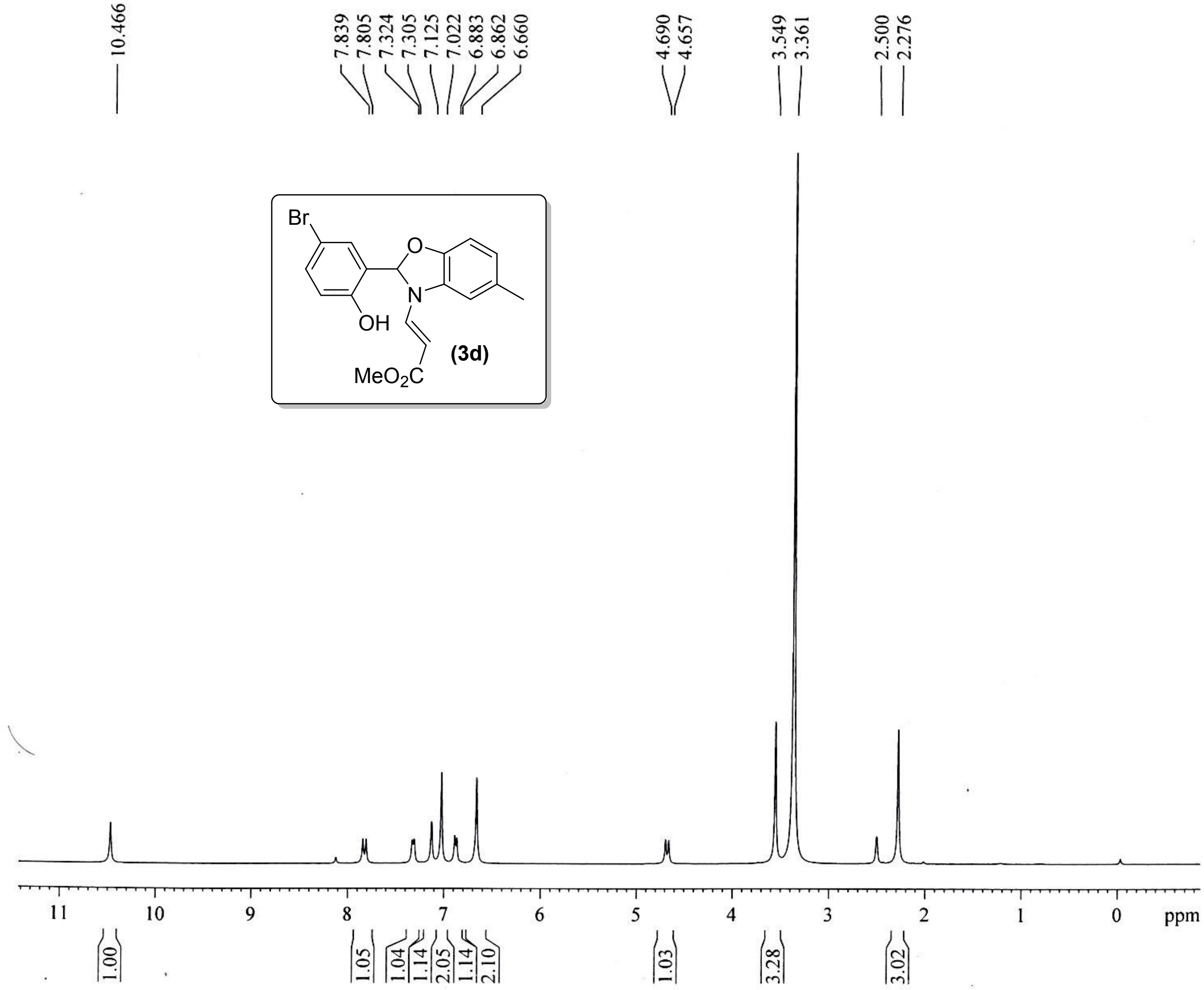
10.466
7.839
7.805
7.324
7.305
7.125
7.022
6.883
6.862
6.660
4.690
4.657
3.549
3.361
2.500
2.276
Br
O
N
OH
MeO2C
(3d)
11
10
9
8
7
6
5
4
3
2
1
0
ppm
1.00
1.05
1.04
1.14
2.05
1.14
2.10
1.03
3.28
3.02

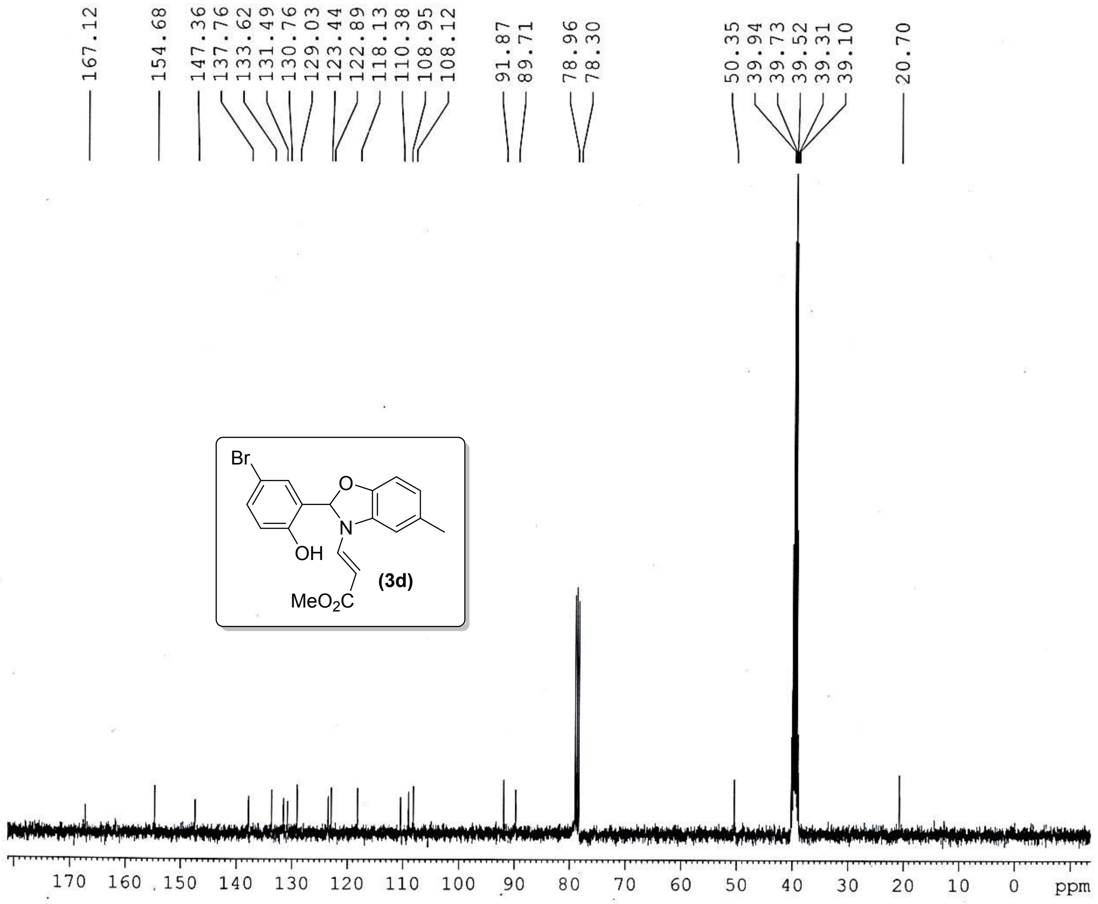
167.12
154.68
147.36
137.76
133.62
131.49
130.76
129.03
123.44
122.89
118.13
110.38
108.95
108.12
91.87
89.71
78.96
78.30
50.35
39.94
39.73
39.52
39.31
39.10
20.70
Br
O
N
OH
MeO$_2$C
(3d)
170 160 150 140 130 120 110 100 90 80 70 60 50 40 30 20 10 0 ppm

Sample Name	MM-137	Position		Instrument Name	Q-TOF	User Name	QTOF-PU\admin
Inj Vol	-1	InjPosition		SampleType	Sample	IRM Calibration Status	Success
Data Filename	MM-137.d	ACQ Method	Pondicherry Universi				

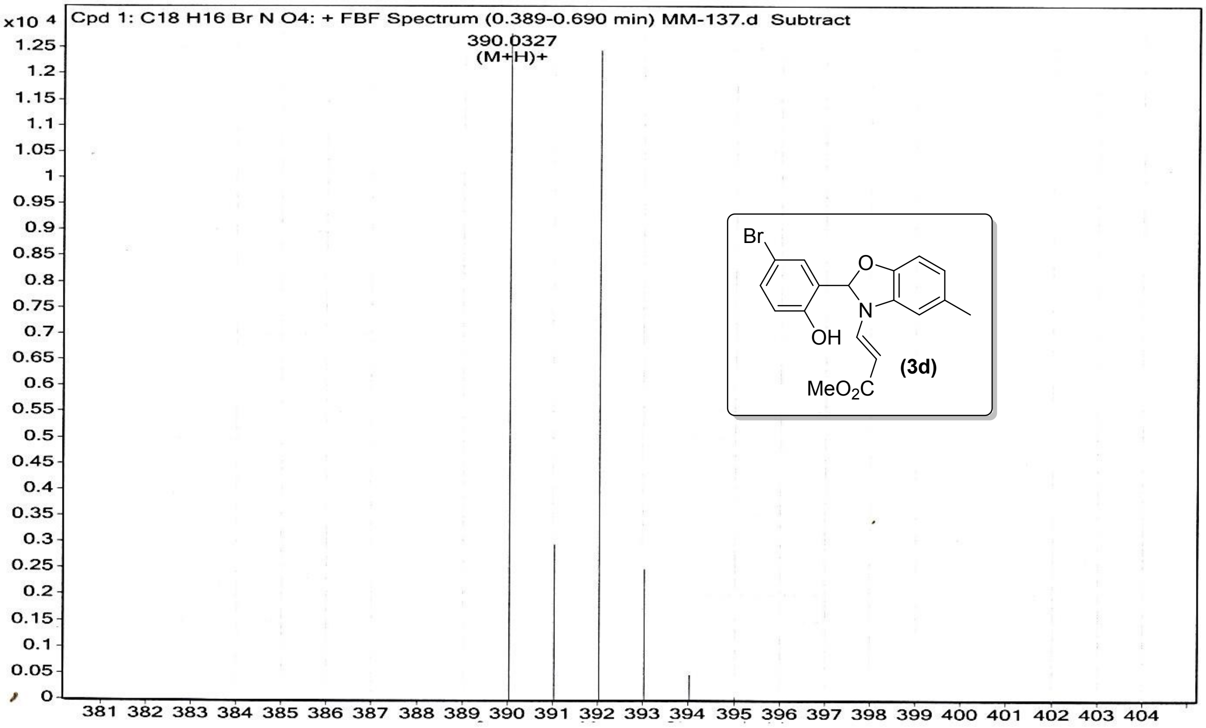

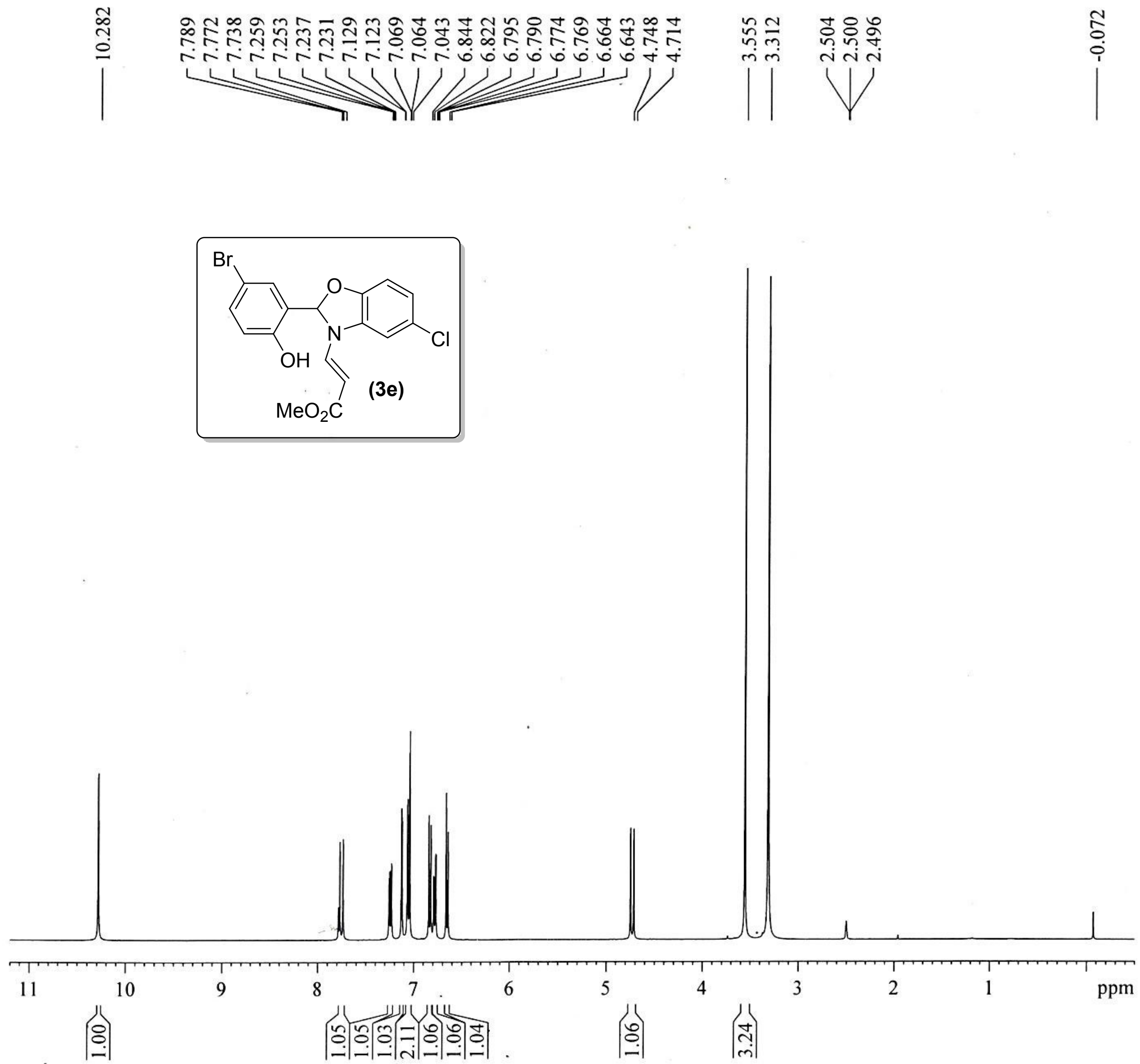
10.282
7.789
7.772
7.738
7.259
7.253
7.237
7.231
7.129
7.123
7.069
7.064
7.043
6.844
6.822
6.795
6.790
6.774
6.769
6.664
6.643
4.748
4.714
3.555
3.312
2.504
2.500
2.496
-0.072
Br
O
N
Cl
OH
MeO2C
(3e)
11
10
9
8
7
6
5
4
3
2
1
ppm
1.00
1.05
1.05
1.03
2.11
1.06
1.06
1.04
1.06
3.24

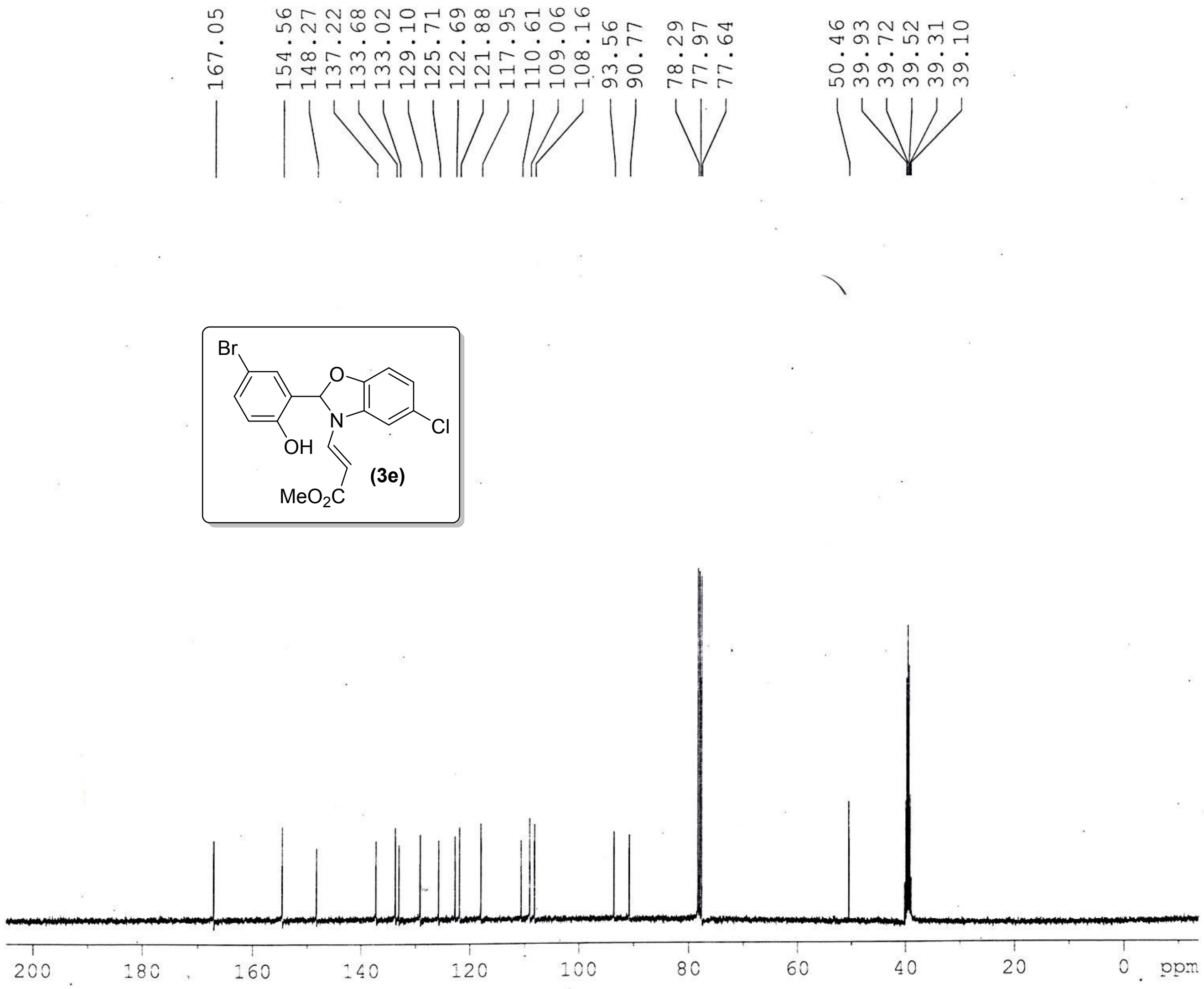

167.05
154.56
148.27
137.22
133.68
133.02
129.10
125.71
122.69
121.88
117.95
110.61
109.06
108.16
93.56
90.77
78.29
77.97
77.64
50.46
39.93
39.72
39.52
39.31
39.10
Br
O
N
Cl
OH
MeO2C
(3e)
200
180
160
140
120
100
80
60
40
20
0
ppm

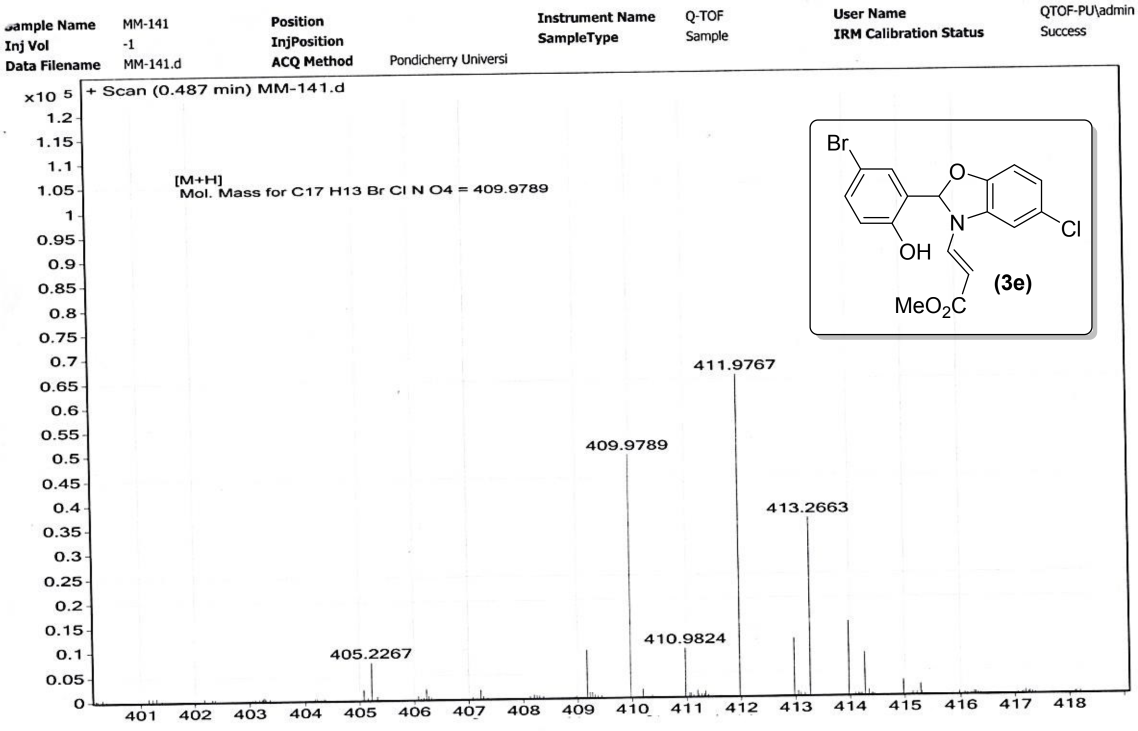
Sample Name MM-141
Inj Vol -1
Data Filename MM-141.d
Position
InjPosition
ACQ Method Pondicherry Universi
Instrument Name Q-TOF
SampleType Sample
User Name QTOF-PU\admin
IRM Calibration Status Success
x10 5
+ Scan (0.487 min) MM-141.d
[M+H]
Mol. Mass for C17 H13 Br Cl N O4 = 409.9789
Br
O
N
Cl
OH
MeO2C
(3e)
411.9767
409.9789
413.2663
410.9824
405.2267

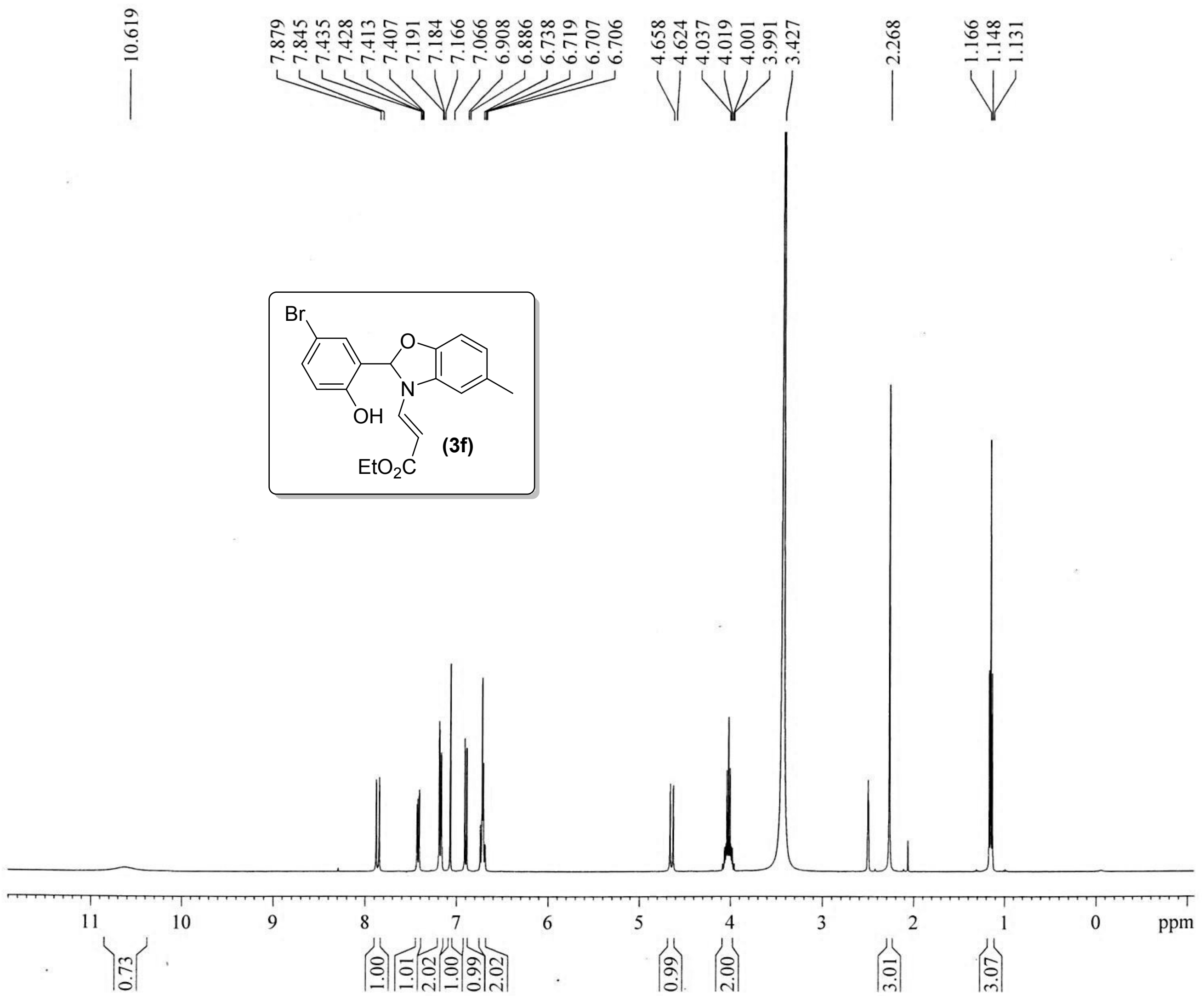
10.619
7.879
7.845
7.435
7.428
7.413
7.407
7.191
7.184
7.166
7.066
6.908
6.886
6.738
6.719
6.707
6.706
4.658
4.624
4.037
4.019
4.001
3.991
3.427
2.268
1.166
1.148
1.131
Br
O
N
OH
EtO2C
(3f)
11
10
9
8
7
6
5
4
3
2
1
0
ppm
0.73
1.00
1.01
2.02
1.00
0.99
2.02
0.99
2.00
3.01
3.07

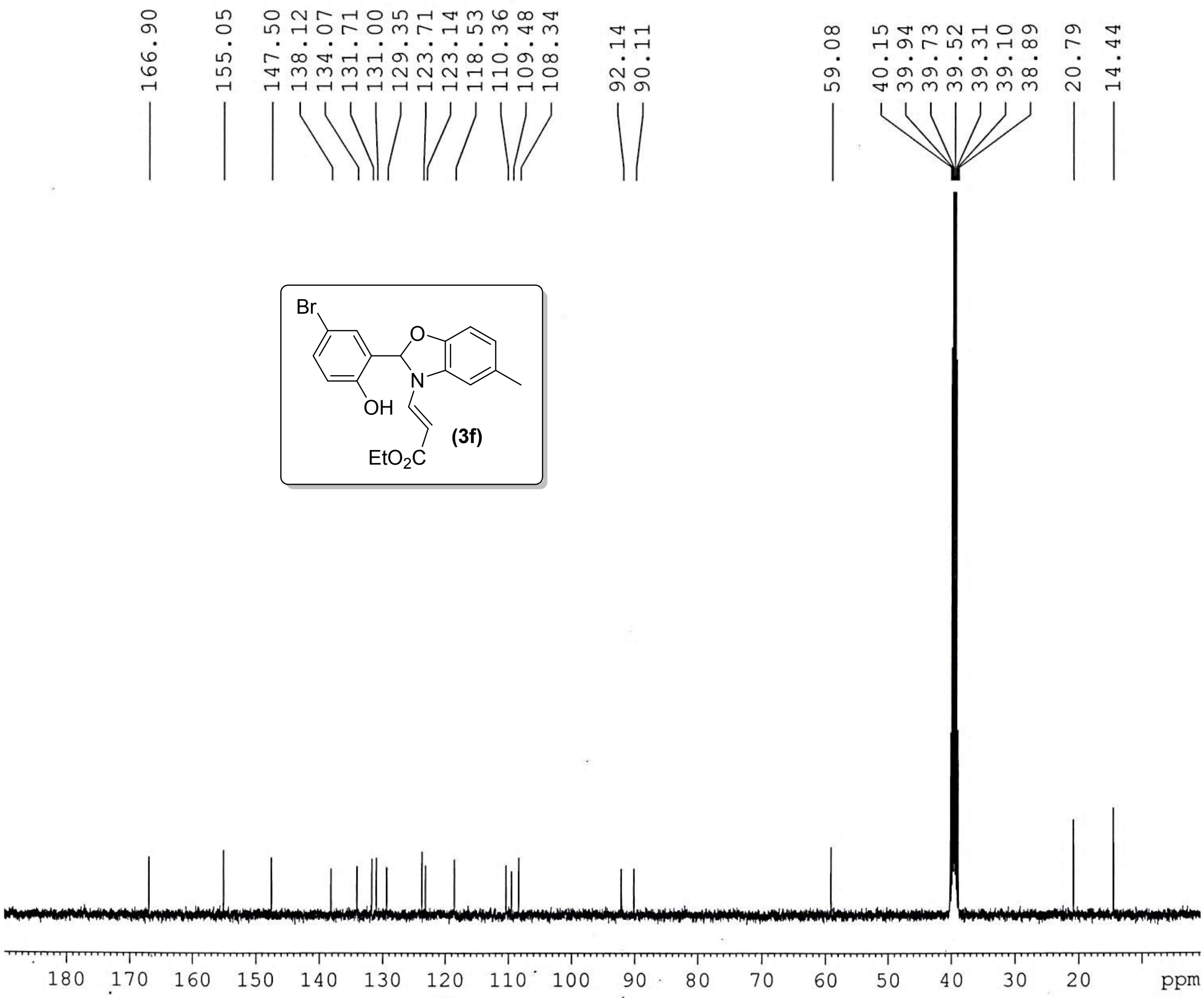
166.90
155.05
147.50
138.12
134.07
131.71
131.00
129.35
123.71
123.14
118.53
110.36
109.48
108.34
92.14
90.11
59.08
40.15
39.94
39.73
39.52
39.31
39.10
38.89
20.79
14.44
Br
O
N
OH
EtO2C
(3f)
180 170 160 150 140 130 120 110 100 90 80 70 60 50 40 30 20 ppm

**Sample Name**	MM-134	**Position**		**Instrument Name**	Q-TOF	**User Name**	QTOF-PU\admin
**Inj Vol**	-1	**InjPosition**		**SampleType**	Sample	**IRM Calibration Status**	Success
**Data Filename**	MM-134.d	**ACQ Method**	Pondicherry Universi				

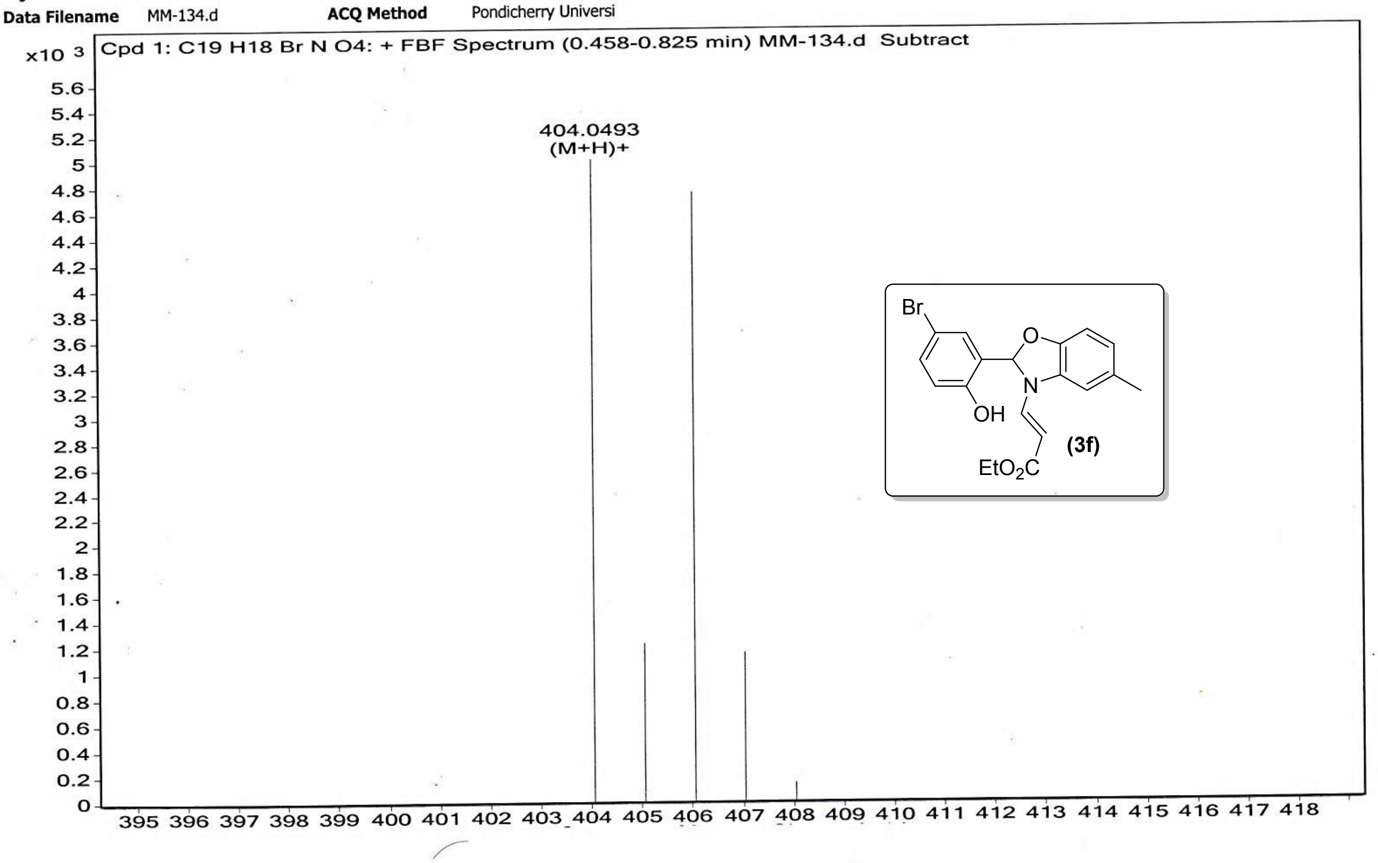

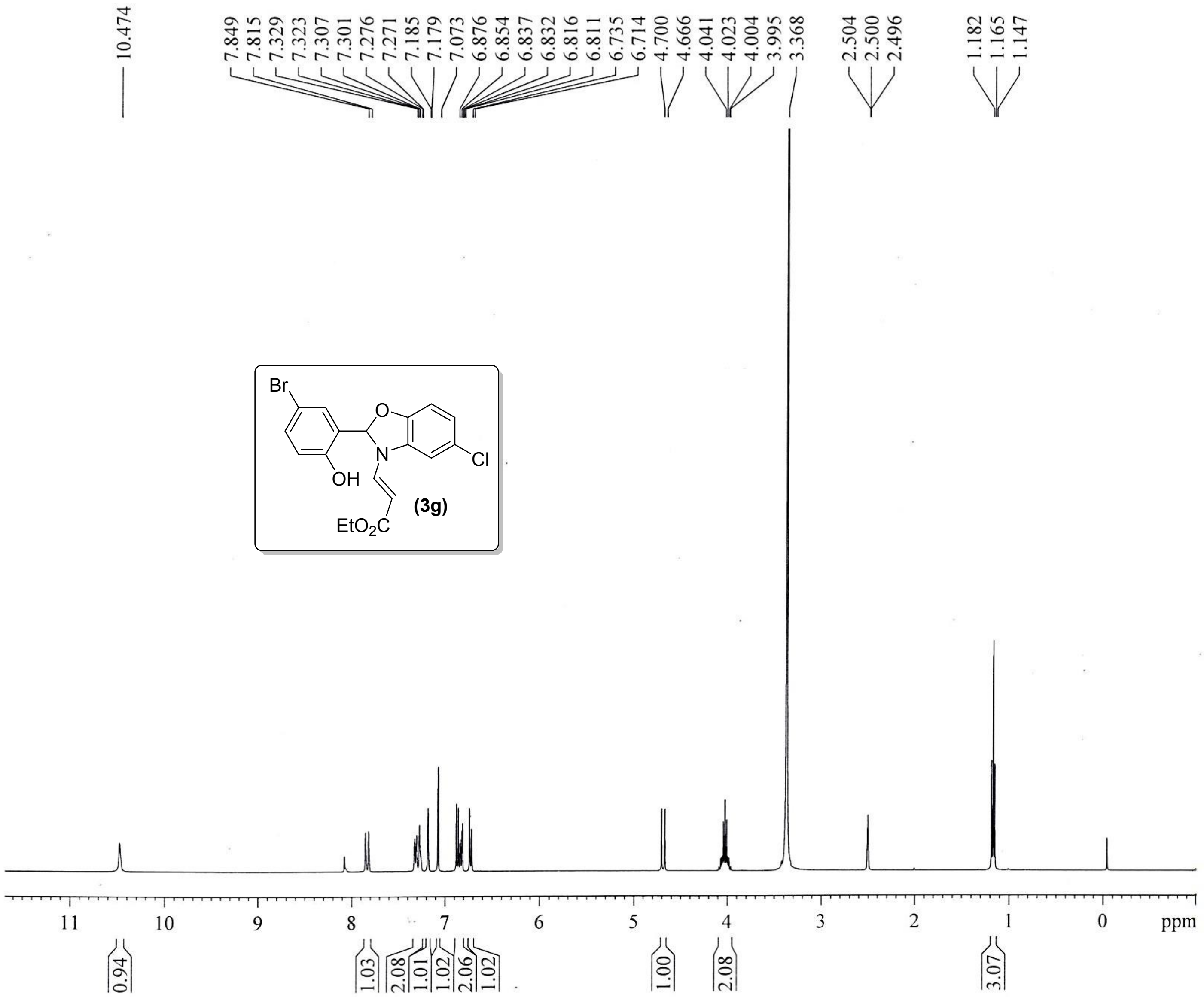
10.474
7.849 7.815 7.329 7.323 7.307 7.301 7.276 7.271 7.185 7.179 7.073 6.876 6.854 6.837 6.832 6.816 6.811 6.735 6.714
4.700 4.666 4.041 4.023 4.004 3.995 3.368
2.504 2.500 2.496
1.182 1.165 1.147
Br
O
N
Cl
OH
EtO2C
(3g)
11 10 9 8 7 6 5 4 3 2 1 0 ppm
0.94
1.03 2.08 1.01 1.02 2.06 1.02
1.00
2.08
3.07

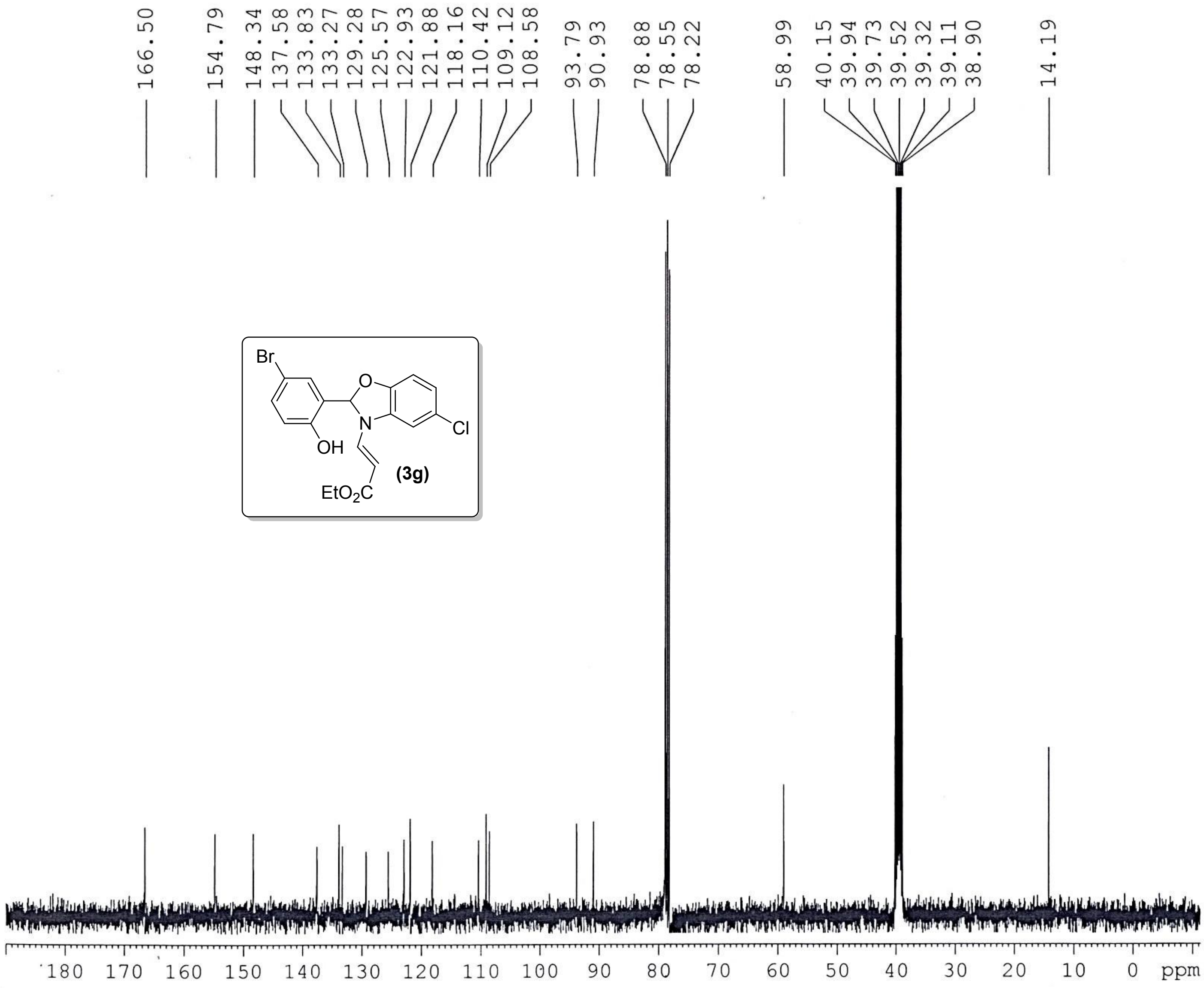
166.50
154.79
148.34
137.58
133.83
133.27
129.28
125.57
122.93
121.88
118.16
110.42
109.12
108.58
93.79
90.93
78.88
78.55
78.22
58.99
40.15
39.94
39.73
39.52
39.32
39.11
38.90
14.19
Br
O
N
Cl
OH
EtO2C
(3g)
180 170 160 150 140 130 120 110 100 90 80 70 60 50 40 30 20 10 0 ppm

**Sample Name**	MM-140	**Position**		**Instrument Name**	Q-TOF	**User Name**	QTOF-PU\admin
**Inj Vol**	-1	**InjPosition**		**SampleType**	Sample	**IRM Calibration Status**	Success
**Data Filename**	MM-140.d	**ACQ Method**	Pondicherry Universi				

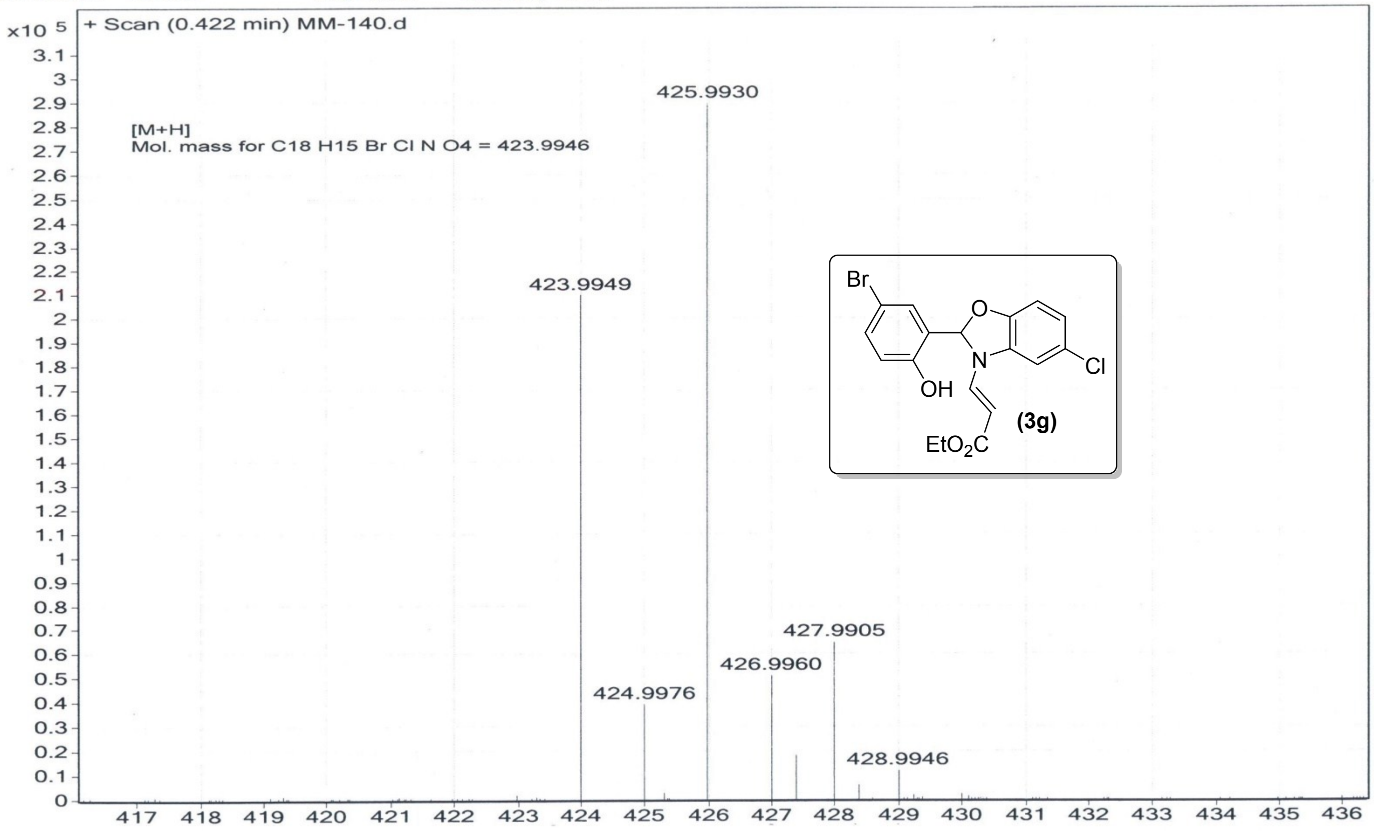

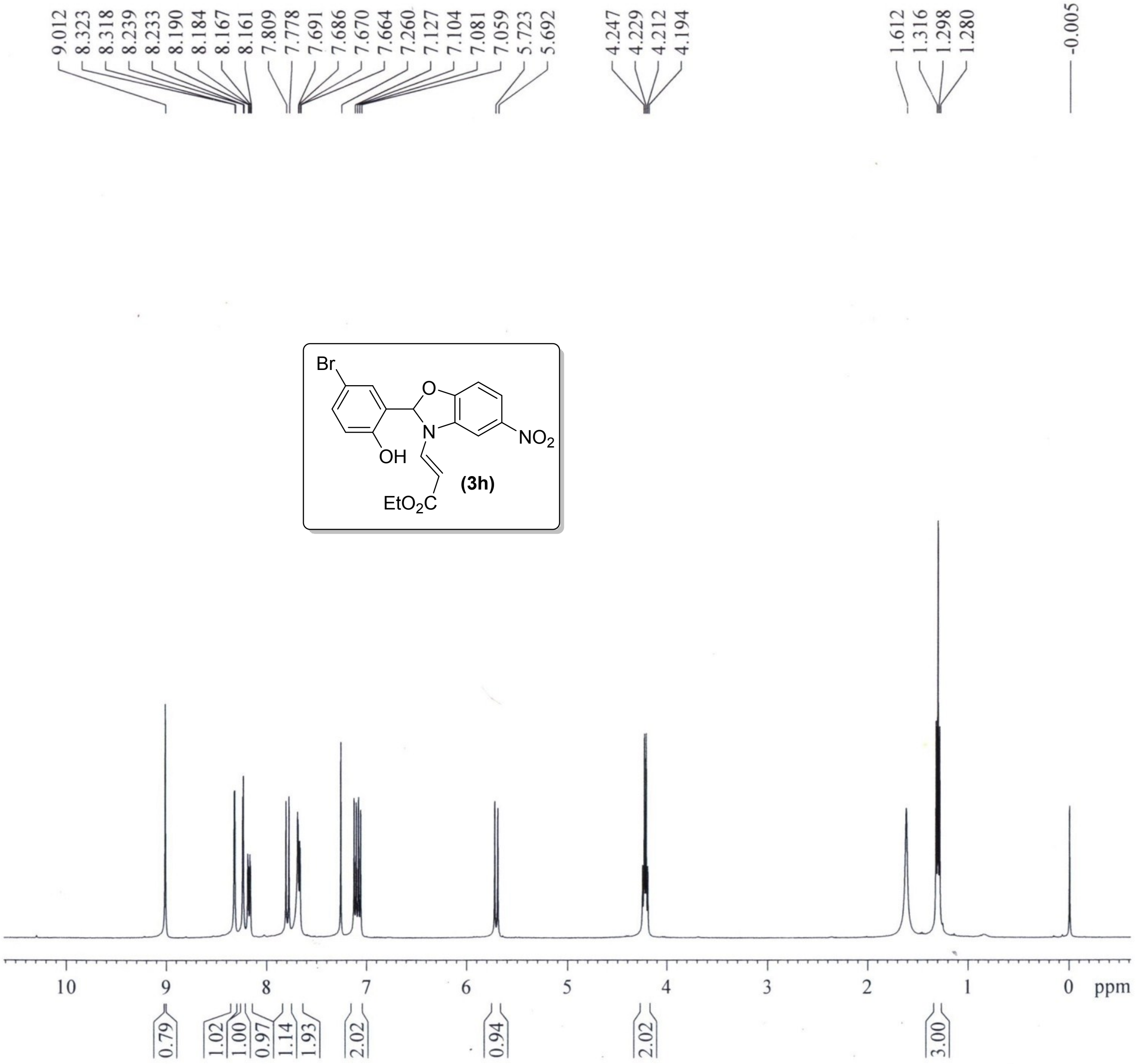
9.012
8.323
8.318
8.239
8.233
8.190
8.184
8.167
8.161
7.809
7.778
7.691
7.686
7.670
7.664
7.260
7.127
7.104
7.081
7.059
5.723
5.692
4.247
4.229
4.212
4.194
1.612
1.316
1.298
1.280
-0.005
Br
O
N
NO2
OH
EtO2C
(3h)
10
9
8
7
6
5
4
3
2
1
0
ppm
0.79
1.02
1.00
0.97
1.14
1.93
2.02
0.94
2.02
3.00

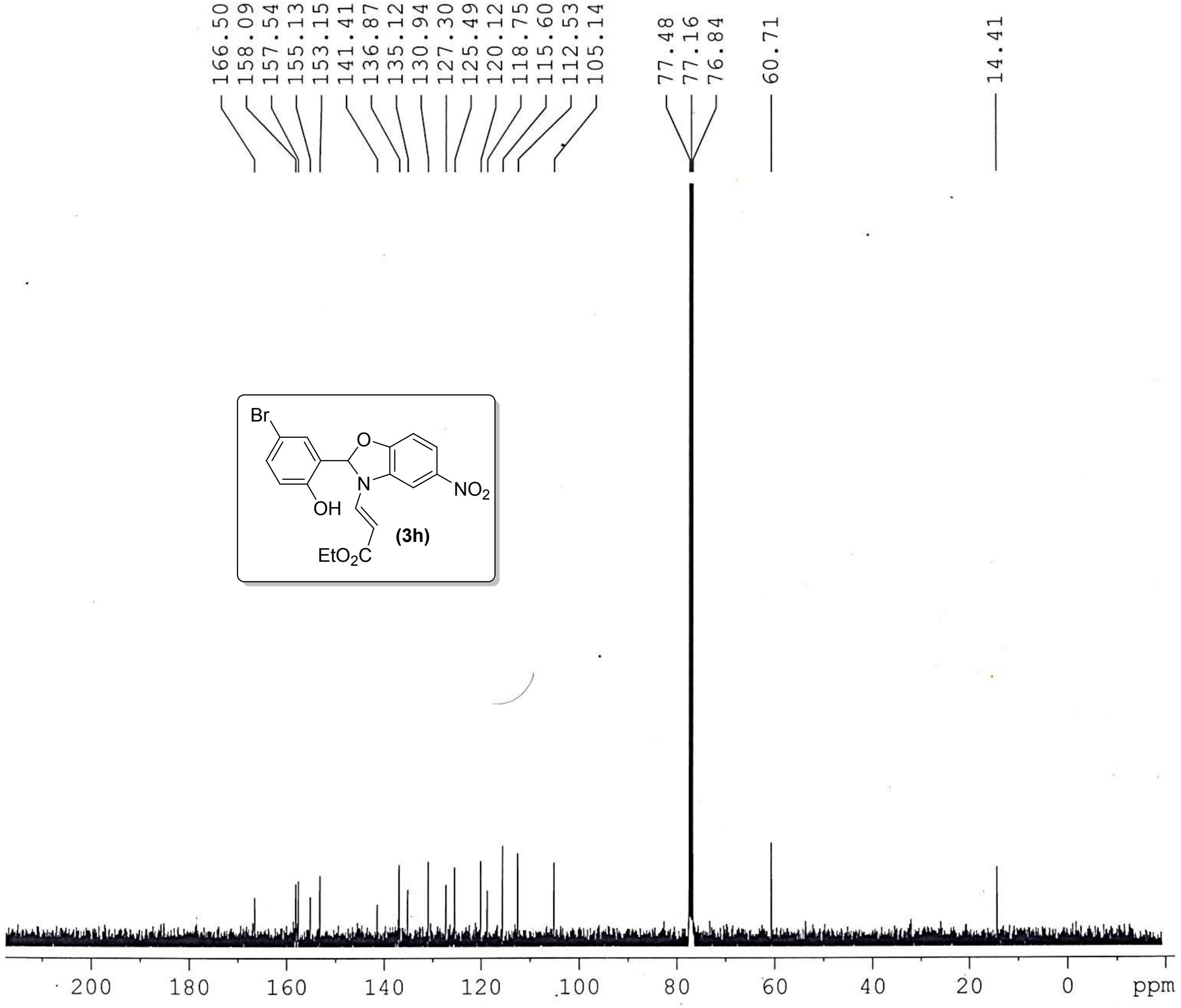

166.50
158.09
157.54
155.13
153.15
141.41
136.87
135.12
130.94
127.30
125.49
120.12
118.75
115.60
112.53
105.14
77.48
77.16
76.84
60.71
14.41
Br
O
N
NO2
OH
(3h)
EtO2C
200
180
160
140
120
100
80
60
40
20
0
ppm

**Sample Name**	MM-156	**Position**		**Instrument Name**	Q-TOF	**User Name**	QTOF-PU\admin
**Inj Vol**	-1	**InjPosition**		**SampleType**	Sample	**IRM Calibration Status**	Success
**Data Filename**	MM-156.d	**ACQ Method**	Pondicherry Universi				

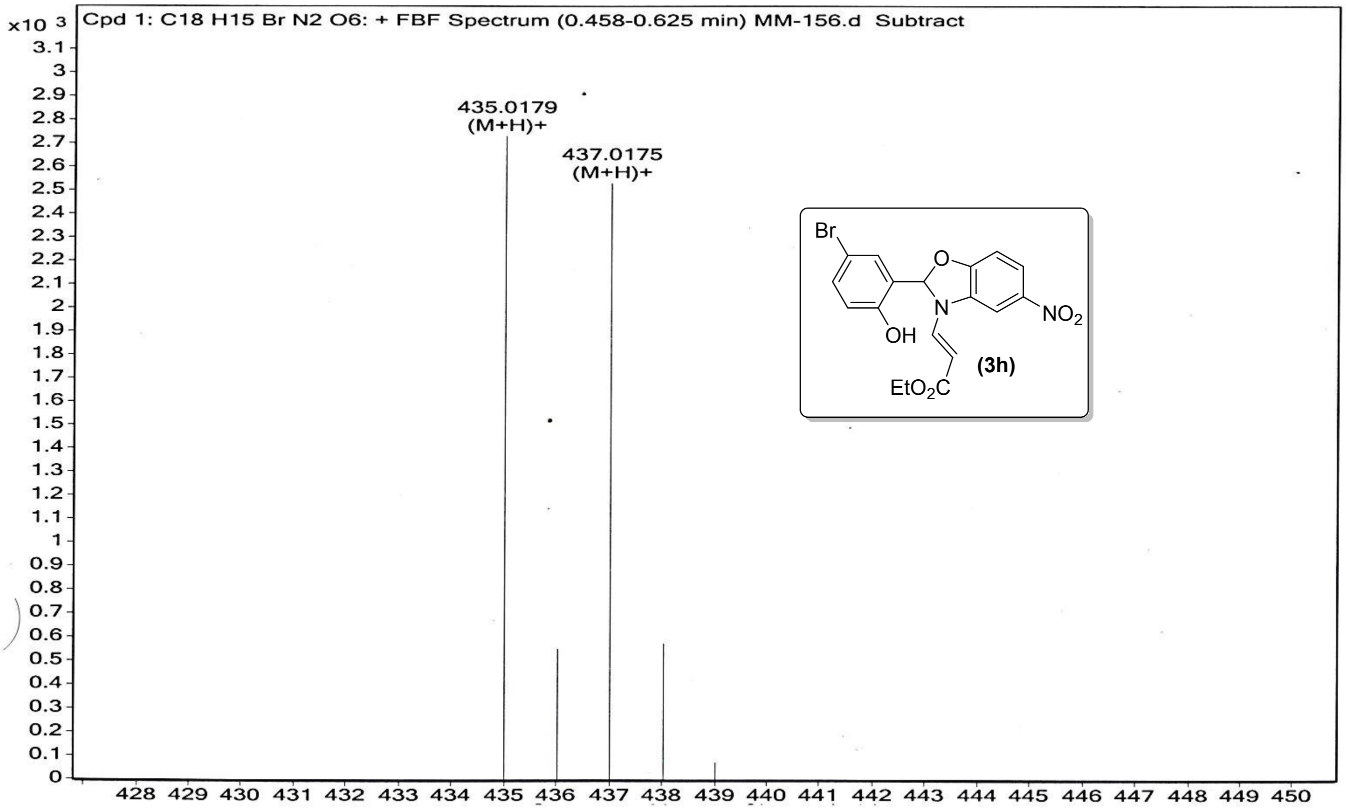

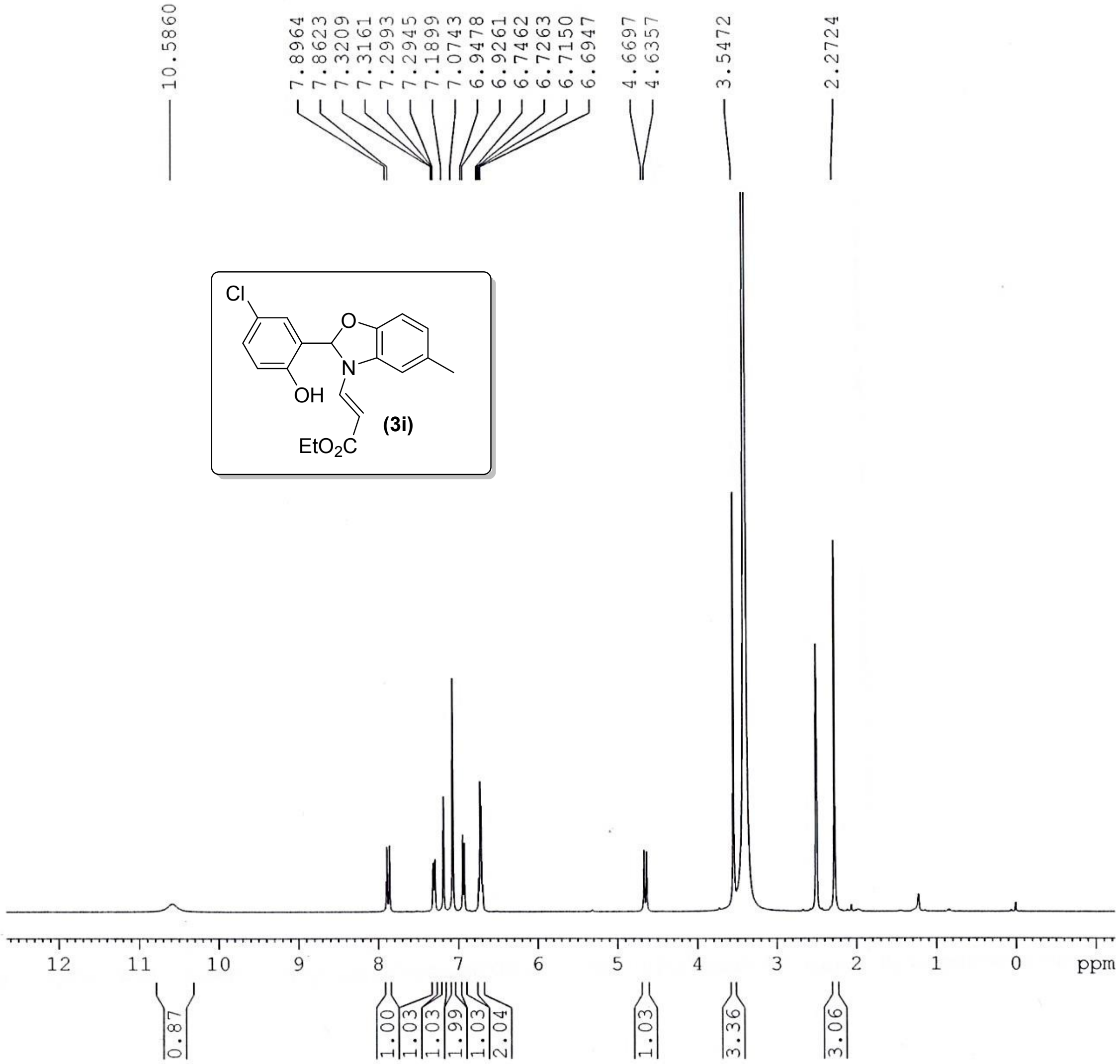
10.5860
7.8964
7.8623
7.3209
7.3161
7.2993
7.2945
7.1899
7.0743
6.9478
6.9261
6.7462
6.7263
6.7150
6.6947
4.6697
4.6357
3.5472
2.2724
Cl
O
N
OH
EtO2C
(3i)
12
11
10
9
8
7
6
5
4
3
2
1
0
ppm
0.87
1.00
1.03
1.03
1.99
1.03
2.04
1.03
3.36
3.06

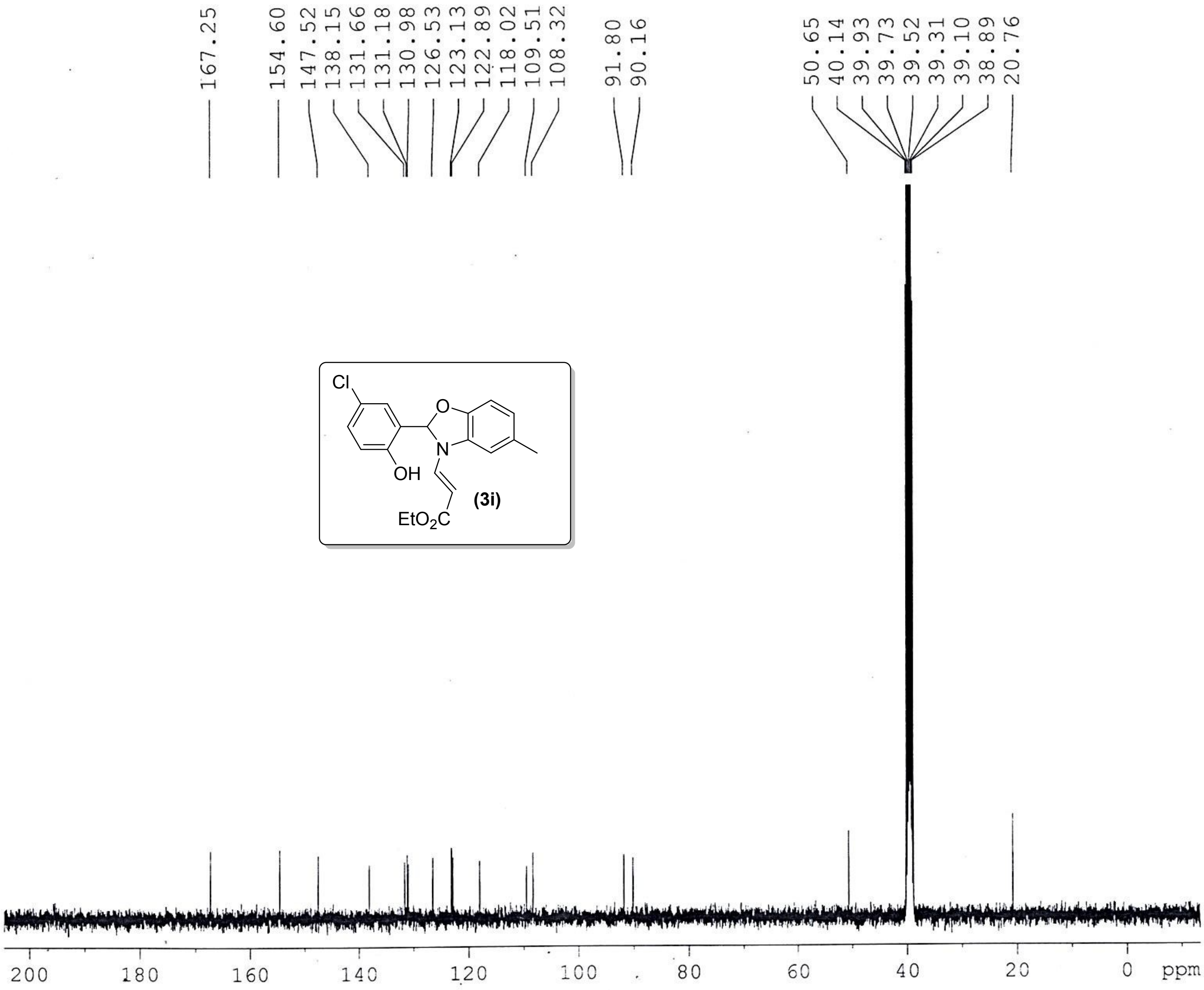
167.25
154.60
147.52
138.15
131.66
131.18
130.98
126.53
123.13
122.89
118.02
109.51
108.32
91.80
90.16
50.65
40.14
39.93
39.73
39.52
39.31
39.10
38.89
20.76
Cl
O
N
OH
EtO_2C
(3i)
200
180
160
140
120
100
80
60
40
20
0
ppm

**Sample Name**	MM-162	**Position**		**Instrument Name**	Q-TOF	**User Name**	QTOF-PU\admin
**Inj Vol**	-1	**InjPosition**		**SampleType**	Sample	**IRM Calibration Status**	Success
**Data Filename**	MM-162.d	**ACQ Method**	Pondicherry Universi				

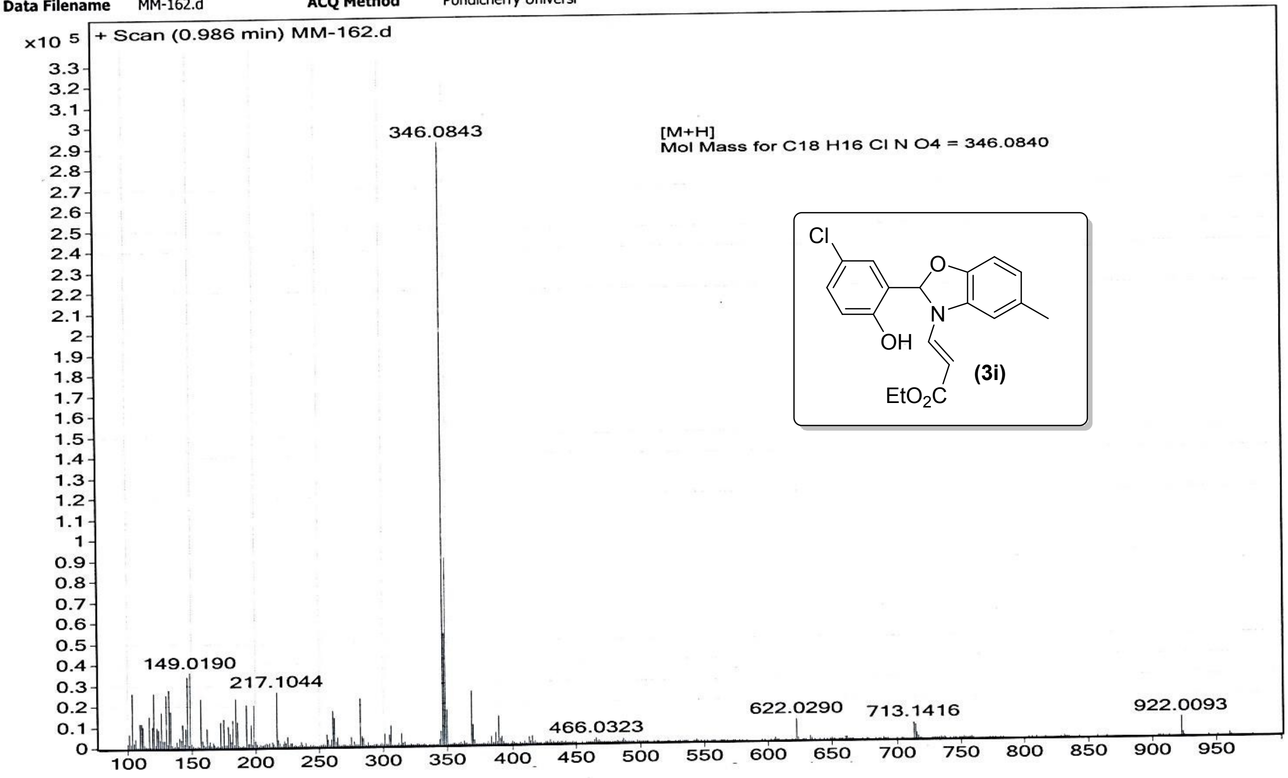

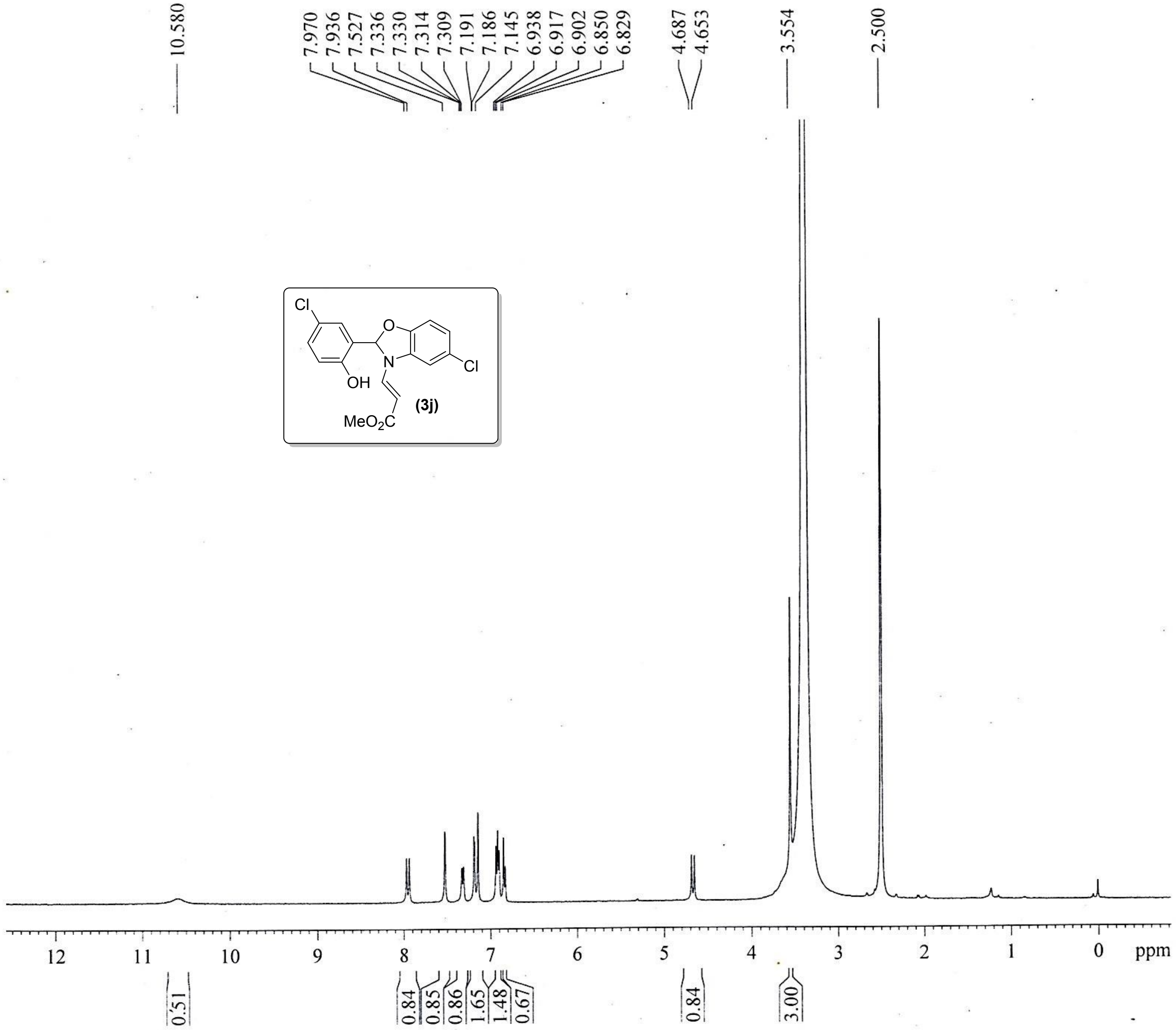
10.580
7.970
7.936
7.527
7.336
7.330
7.314
7.309
7.191
7.186
7.145
6.938
6.917
6.902
6.850
6.829
4.687
4.653
3.554
2.500
Cl
O
N
Cl
OH
MeO2C
(3j)
12 11 10 9 8 7 6 5 4 3 2 1 0 ppm
0.51
0.84
0.85
0.86
1.65
1.48
0.67
0.84
3.00

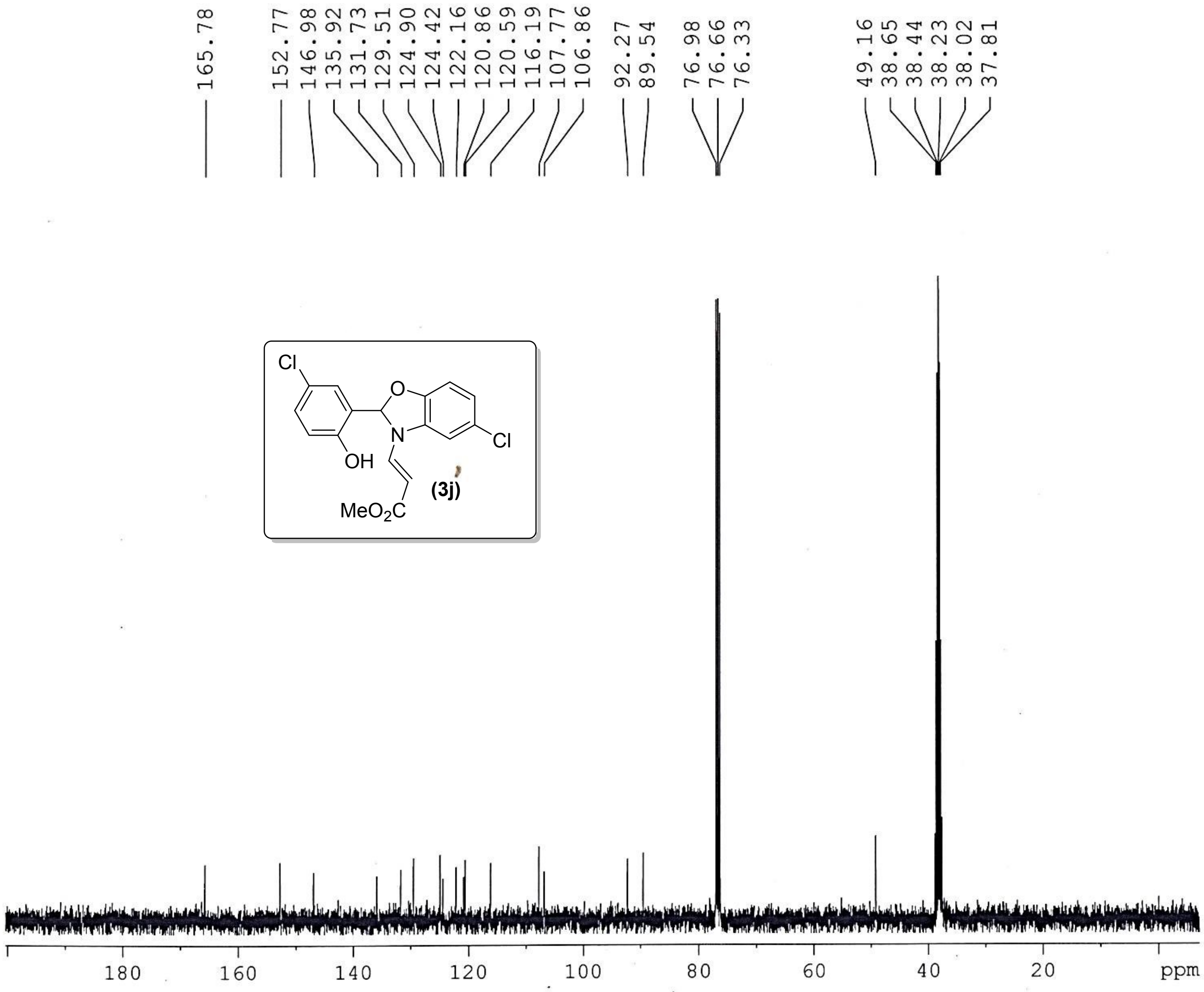
165.78
152.77
146.98
135.92
131.73
129.51
124.90
124.42
122.16
120.86
120.59
116.19
107.77
106.86
92.27
89.54
76.98
76.66
76.33
49.16
38.65
38.44
38.23
38.02
37.81
Cl
O
N
Cl
OH
MeO2C
(3j)
180
160
140
120
100
80
60
40
20
ppm

**Sample Name**	MM-161	**Position**		**Instrument Name**	Q-TOF	**User Name**	QTOF-PU\admin
**Inj Vol**	-1	**InjPosition**		**SampleType**	Sample	**IRM Calibration Status**	Success
**Data Filename**	MM-161.d	**ACQ Method**	Pondicherry Universi				

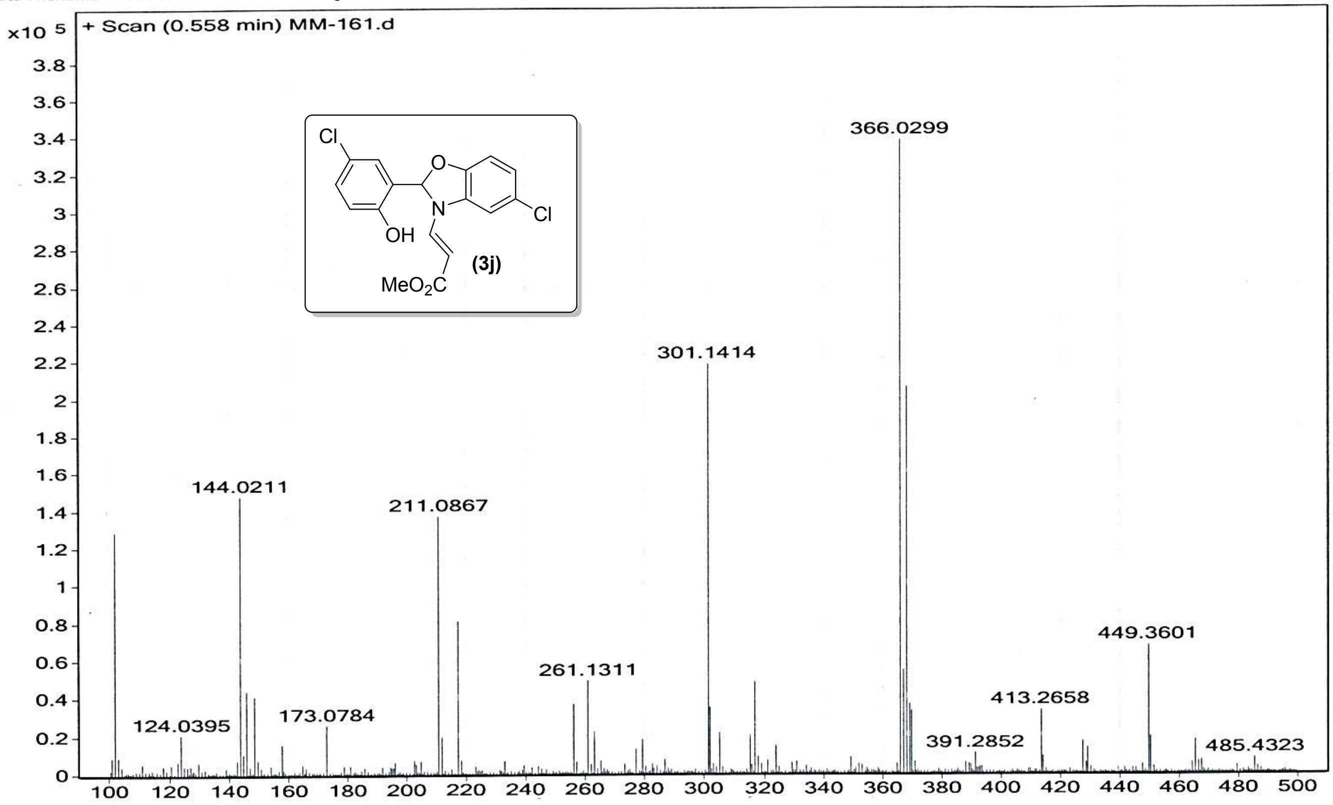

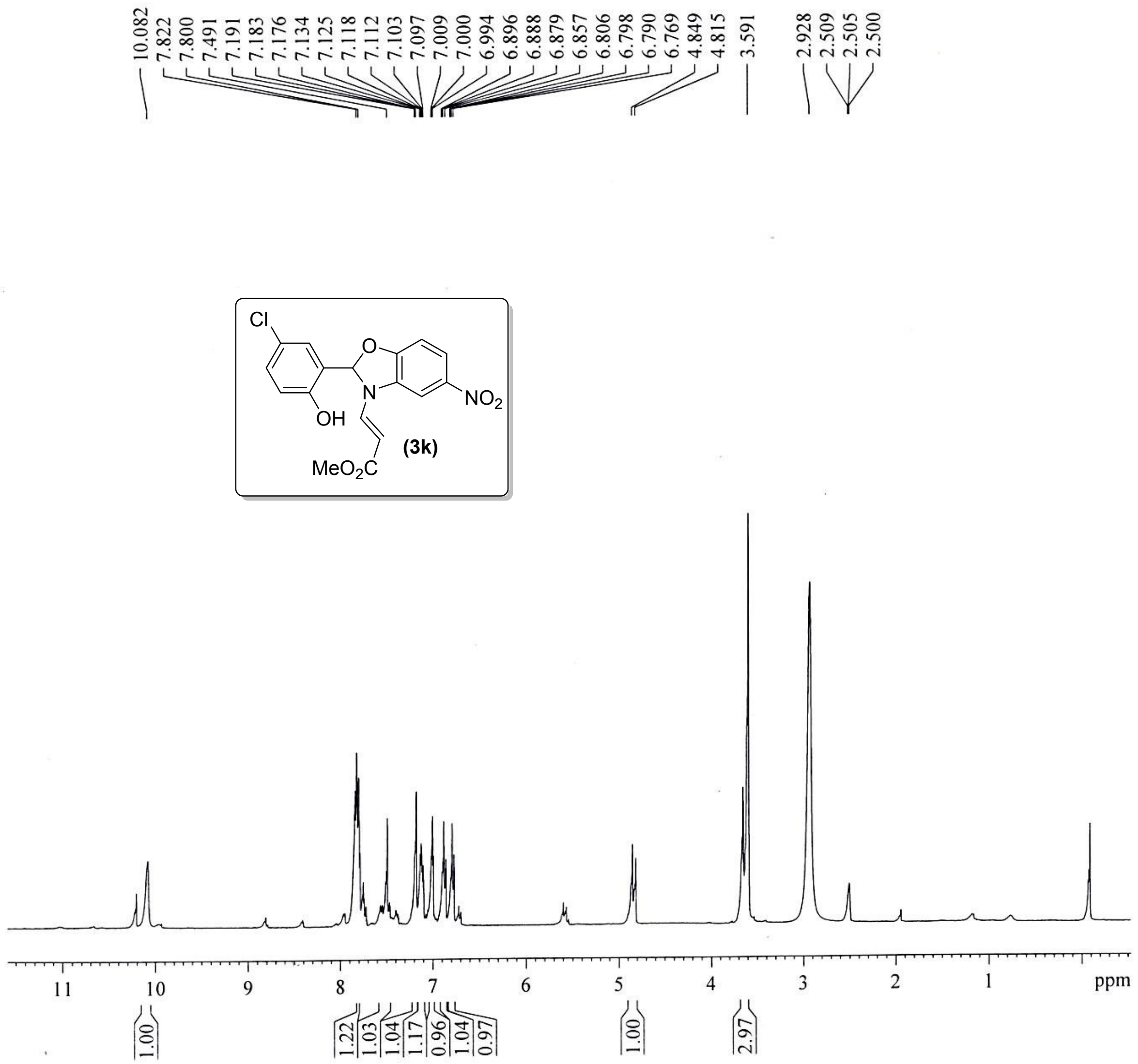
10.082
7.822
7.800
7.491
7.191
7.183
7.176
7.134
7.125
7.118
7.112
7.103
7.097
7.009
7.000
6.994
6.896
6.888
6.879
6.857
6.806
6.798
6.790
6.769
4.849
4.815
3.591
2.928
2.509
2.505
2.500
Cl
O
N
NO2
OH
(3k)
MeO2C
11
10
9
8
7
6
5
4
3
2
1
ppm
1.00
1.22
1.03
1.04
1.17
0.96
1.04
0.97
1.00
2.97

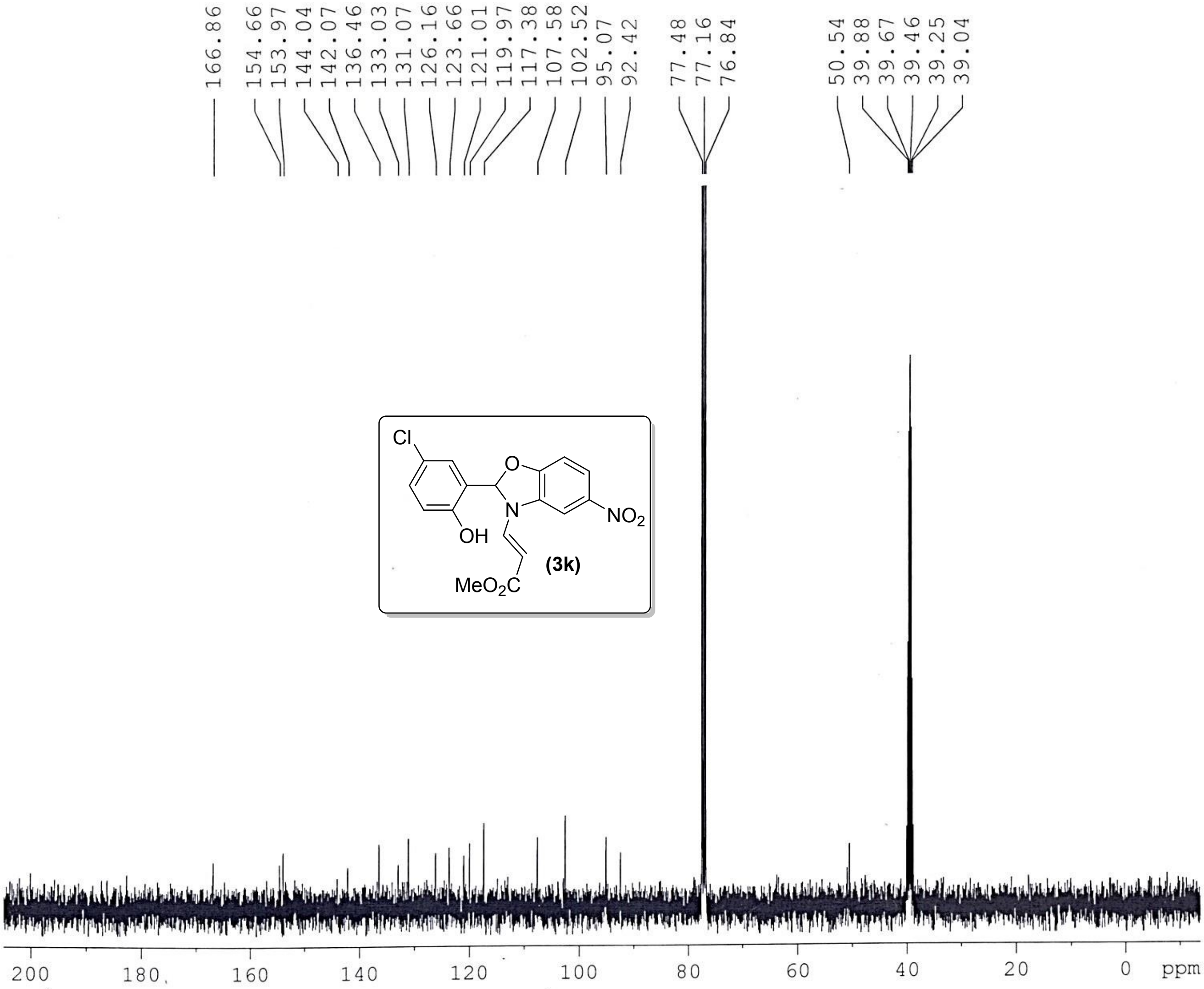

166.86
154.66
153.97
144.04
142.07
136.46
133.03
131.07
126.16
123.66
121.01
119.97
117.38
107.58
102.52
95.07
92.42
77.48
77.16
76.84
50.54
39.88
39.67
39.46
39.25
39.04
Cl
O
N
NO2
OH
MeO2C
(3k)
200
180
160
140
120
100
80
60
40
20
0
ppm

**Sample Name**	MM-164	**Position**		**Instrument Name**	Q-TOF	**User Name**	QTOF-PU\admin
**Inj Vol**	-1	**InjPosition**		**SampleType**	Sample	**IRM Calibration Status**	Success
**Data Filename**	MM-164.d	**ACQ Method**	Pondicherry Universi				

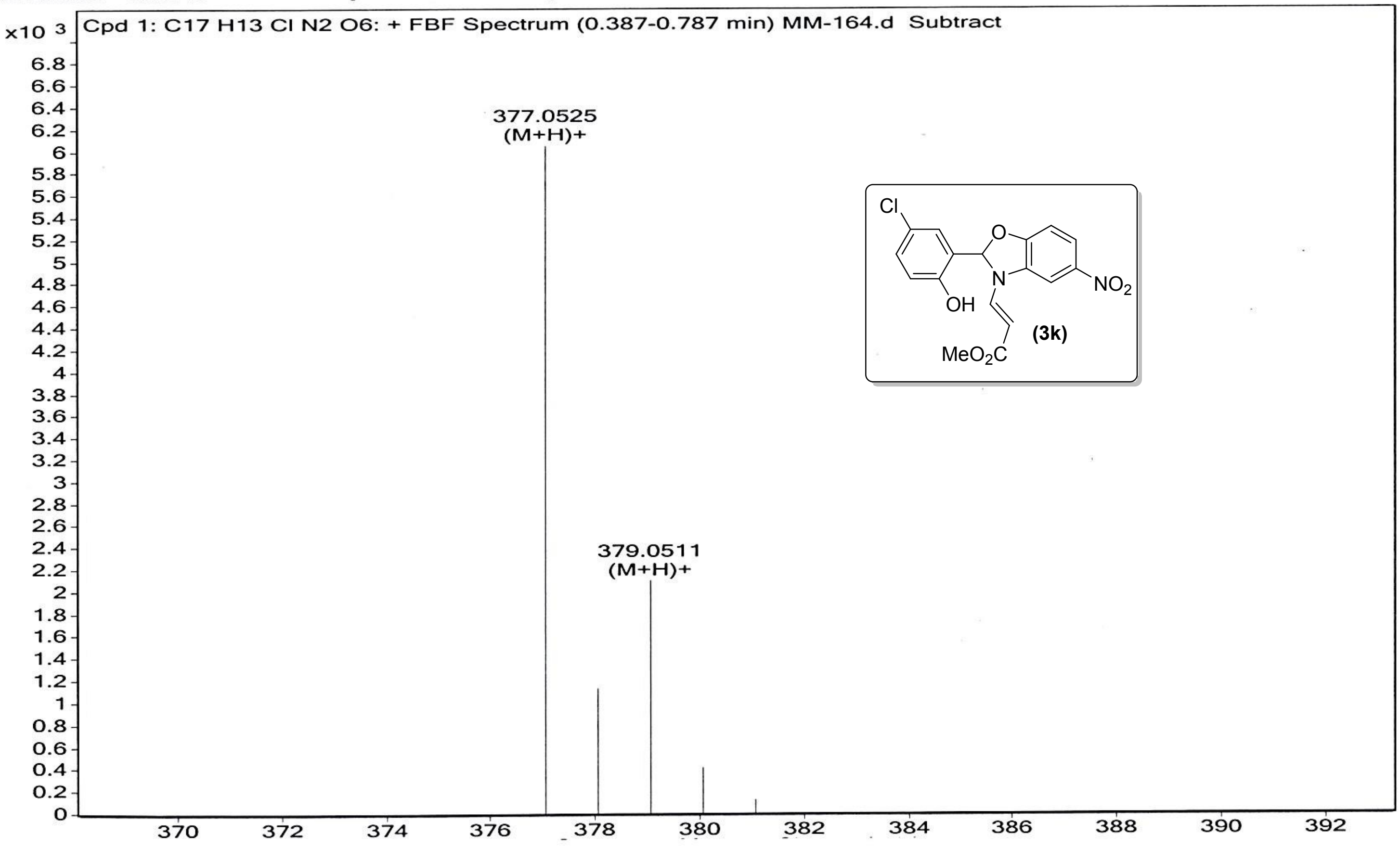

PROTON DMSO {D:\MB} KOPAL 1

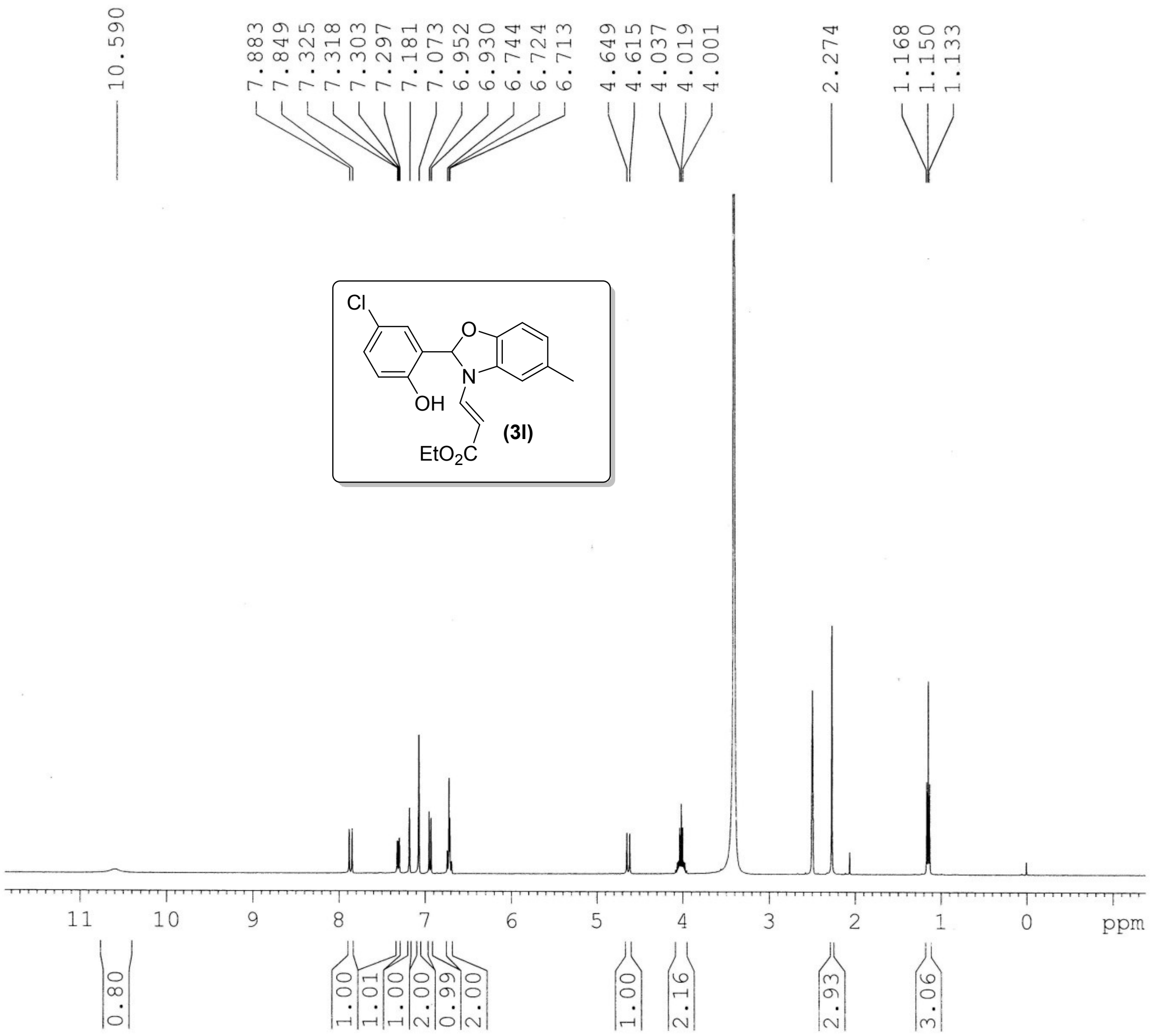

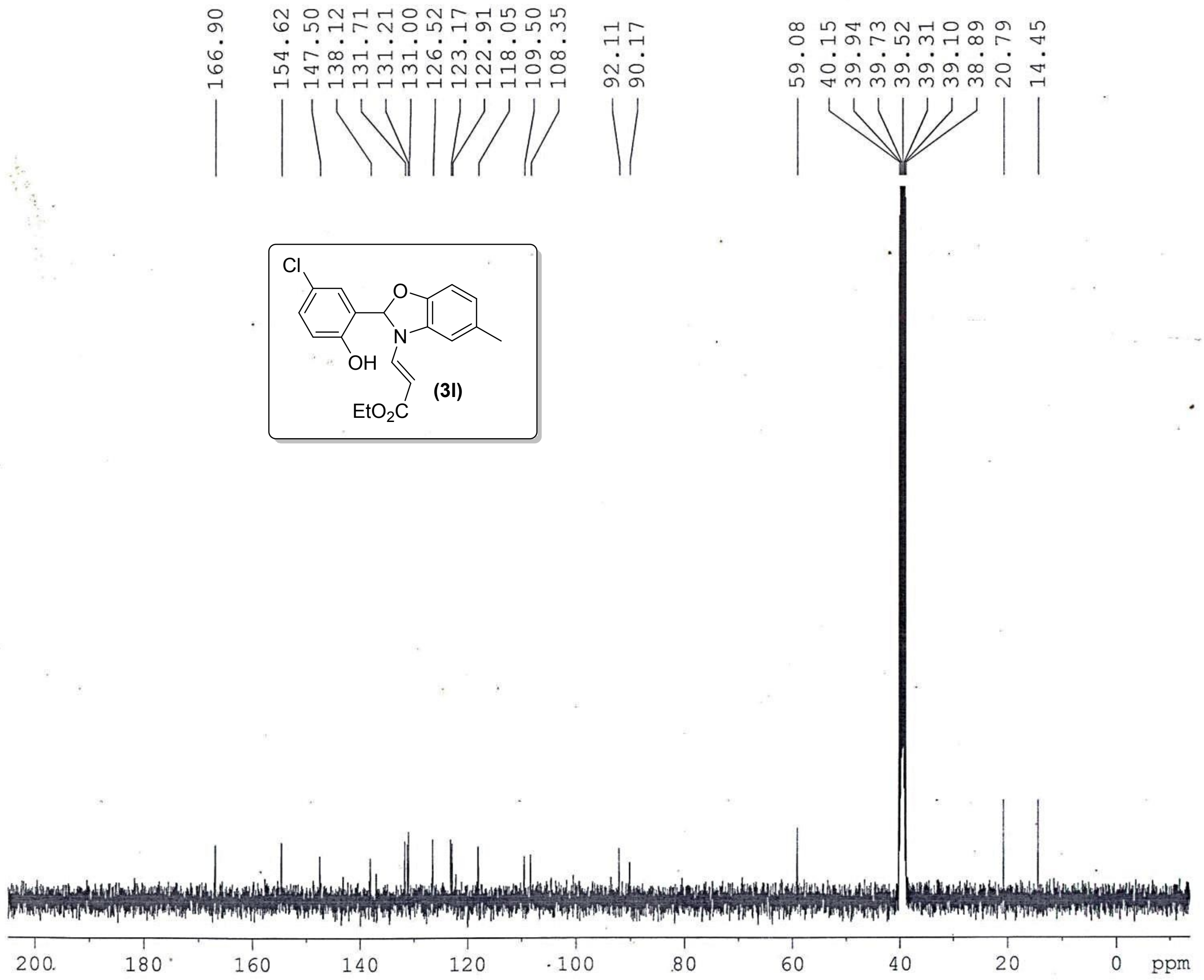
166.90
154.62
147.50
138.12
131.71
131.21
131.00
126.52
123.17
122.91
118.05
109.50
108.35
92.11
90.17
59.08
40.15
39.94
39.73
39.52
39.31
39.10
38.89
20.79
14.45
Cl
O
N
OH
EtO2C
(3l)
200
180
160
140
120
100
80
60
40
20
0
ppm

**Sample Name**	MM-170	**Position**		**Instrument Name**	Q-TOF	**User Name**	QTOF-PU\admin
**Inj Vol**	-1	**InjPosition**		**SampleType**	Sample	**IRM Calibration Status**	Success
**Data Filename**	MM-170.d	**ACQ Method**	Pondicherry Universi				

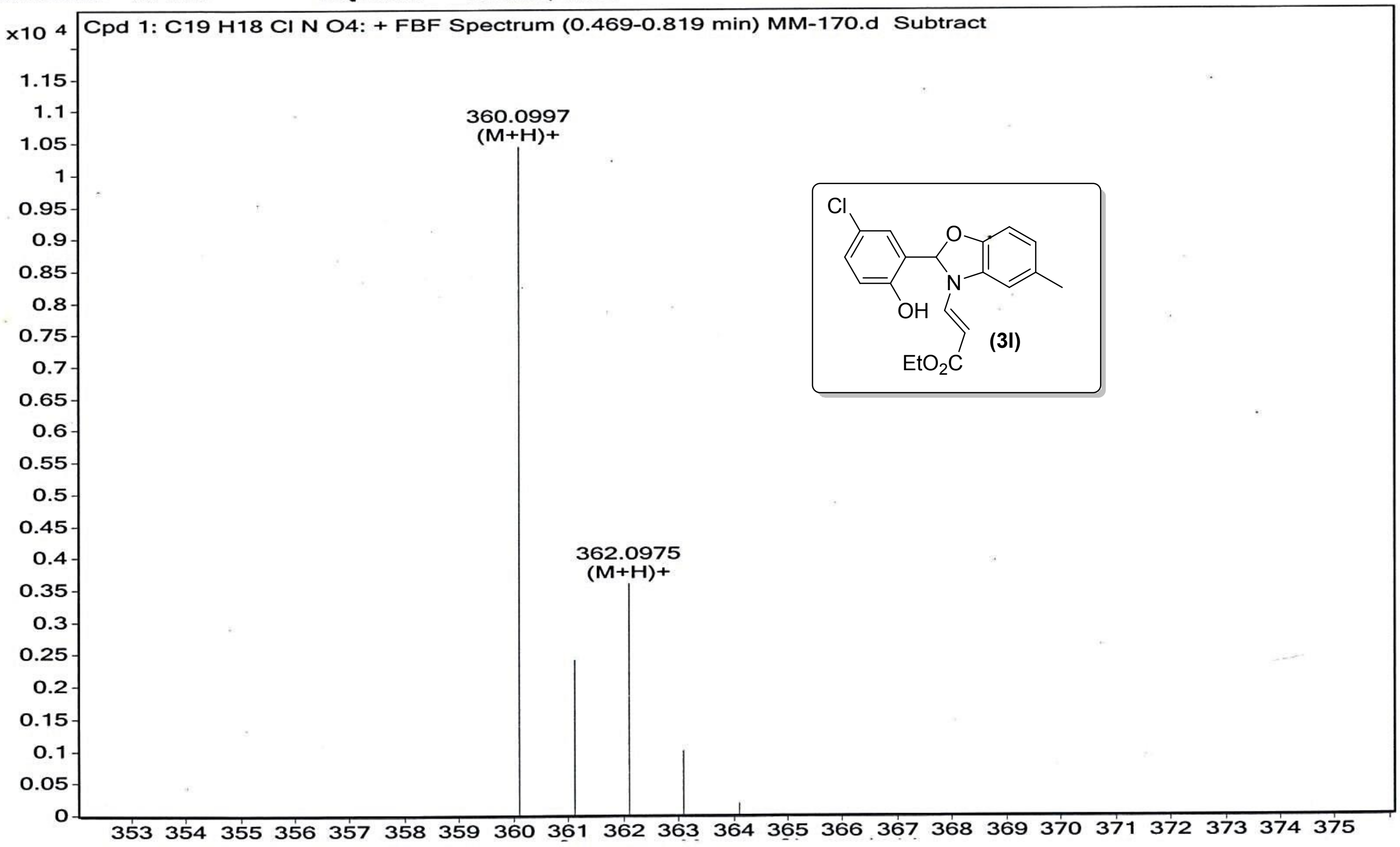

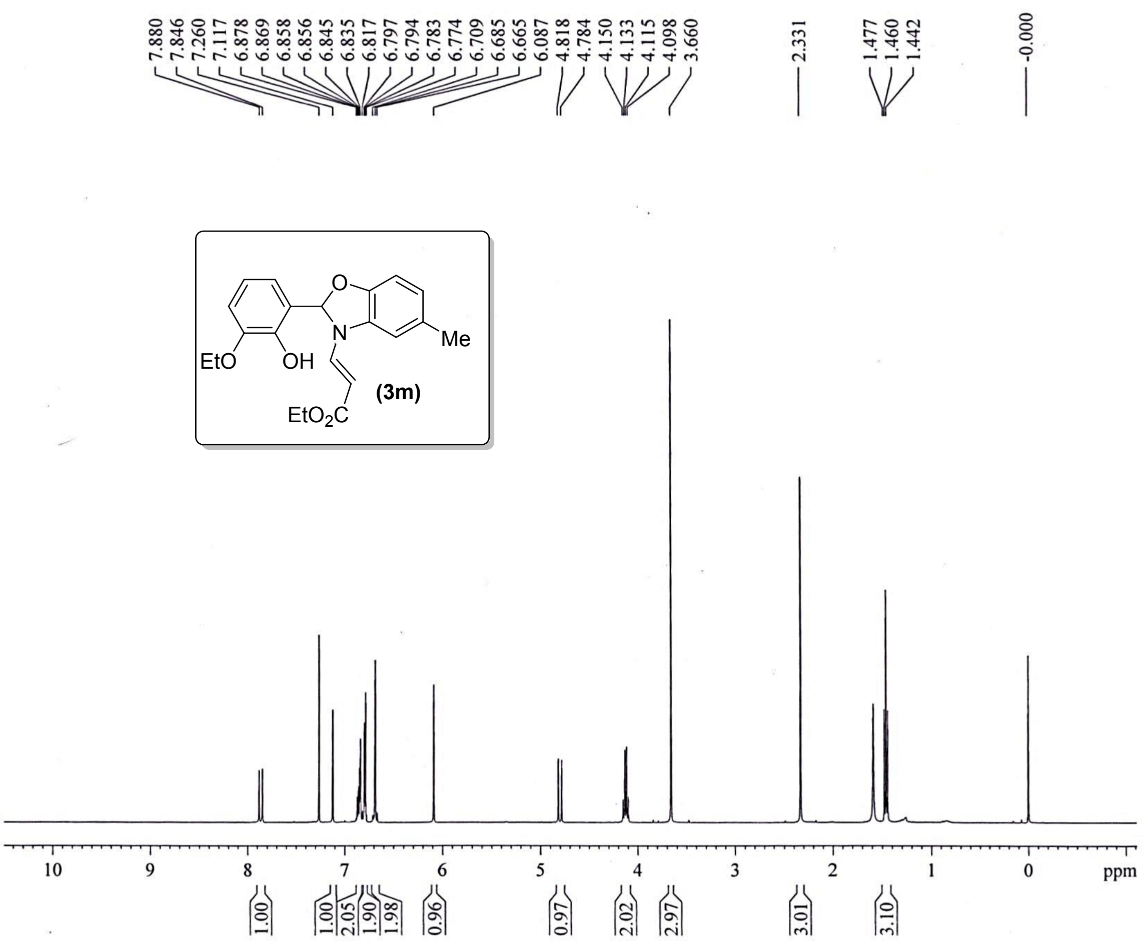
7.880
7.846
7.260
7.117
6.878
6.869
6.858
6.856
6.845
6.835
6.817
6.797
6.794
6.783
6.774
6.709
6.685
6.665
6.087
4.818
4.784
4.150
4.133
4.115
4.098
3.660
2.331
1.477
1.460
1.442
-0.000
EtO
OH
O
N
Me
EtO2C
(3m)
10
9
8
7
6
5
4
3
2
1
0
ppm
1.00
1.00
2.05
1.90
1.98
0.96
0.97
2.02
2.97
3.01
3.10

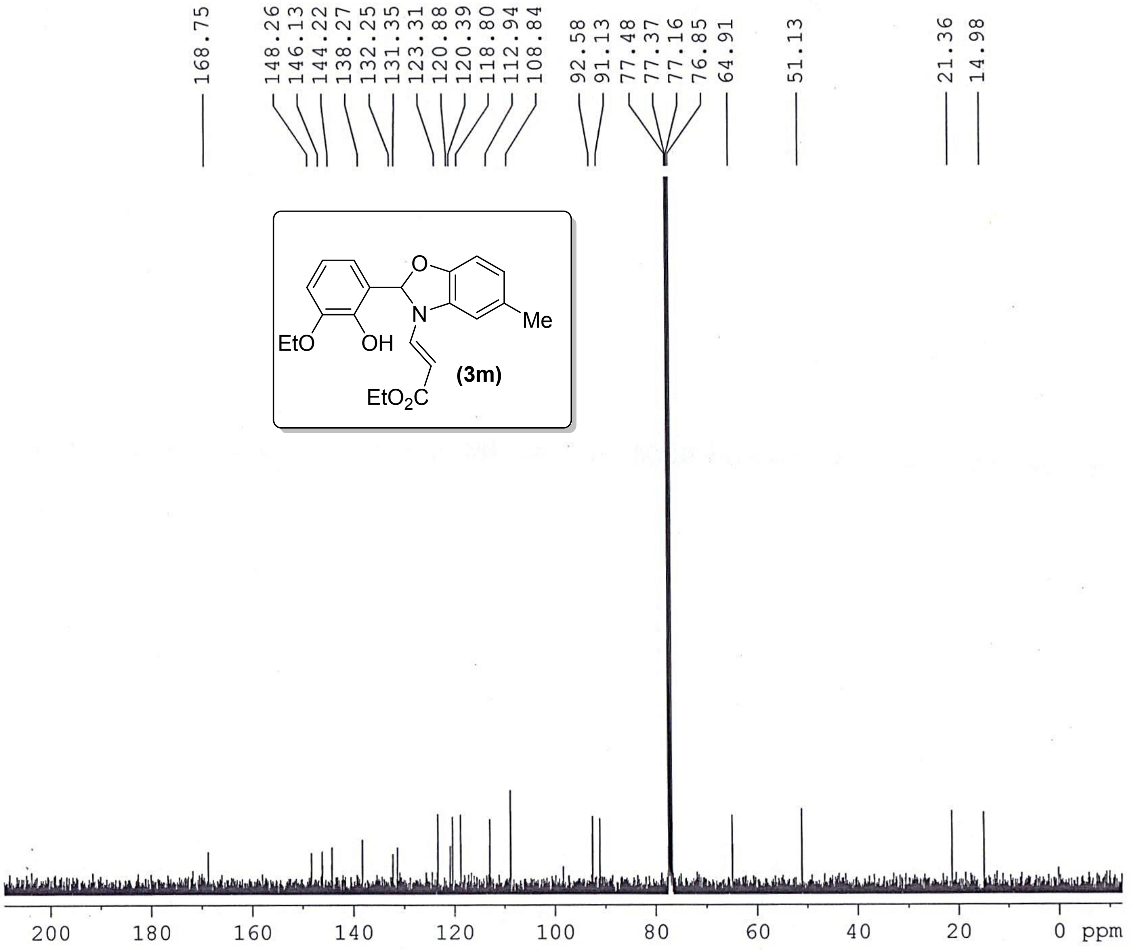
168.75
148.26
146.13
144.22
138.27
132.25
131.35
123.31
120.88
120.39
118.80
112.94
108.84
92.58
91.13
77.48
77.37
77.16
76.85
64.91
51.13
21.36
14.98
EtO
OH
O
N
Me
EtO2C
(3m)
200
180
160
140
120
100
80
60
40
20
0 ppm

**Sample Name**	MM-150	**Position**		**Instrument Name**	Q-TOF	**User Name**	QTOF-PU\admin
**Inj Vol**	-1	**InjPosition**		**SampleType**	Sample	**IRM Calibration Status**	Success
**Data Filename**	MM-150a.d	**ACQ Method**	Pondicherry Universi				

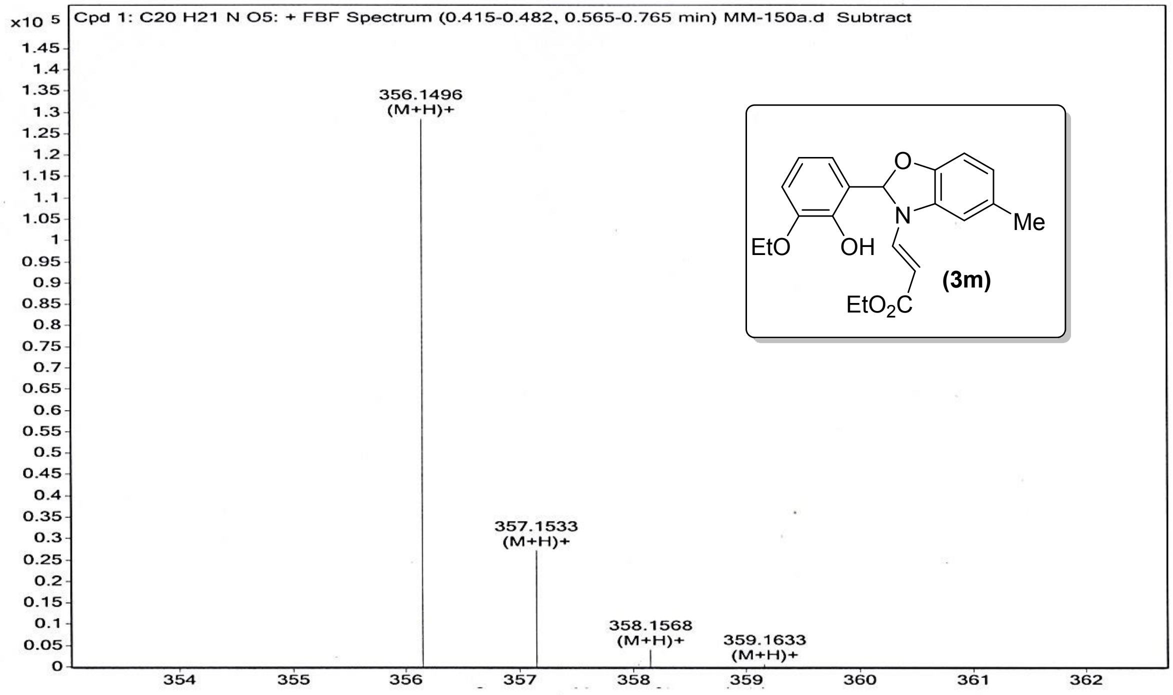

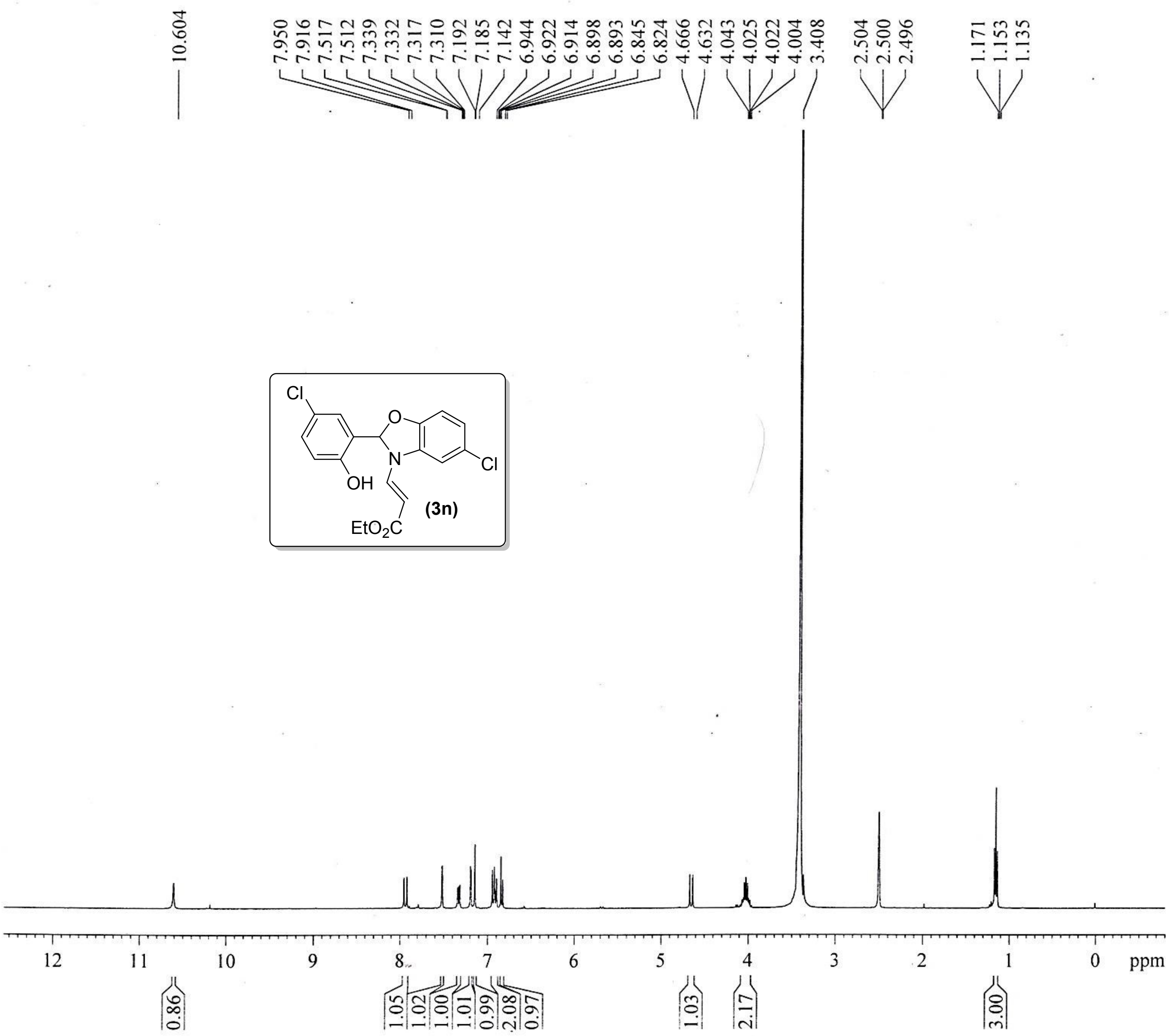
10.604
7.950
7.916
7.517
7.512
7.339
7.332
7.317
7.310
7.192
7.185
7.142
6.944
6.922
6.914
6.898
6.893
6.845
6.824
4.666
4.632
4.043
4.025
4.022
4.004
3.408
2.504
2.500
2.496
1.171
1.153
1.135
Cl
O
N
Cl
OH
EtO2C
(3n)
12
11
10
9
8
7
6
5
4
3
2
1
0
ppm
0.86
1.05
1.02
1.00
1.01
0.99
2.08
0.97
1.03
2.17
3.00

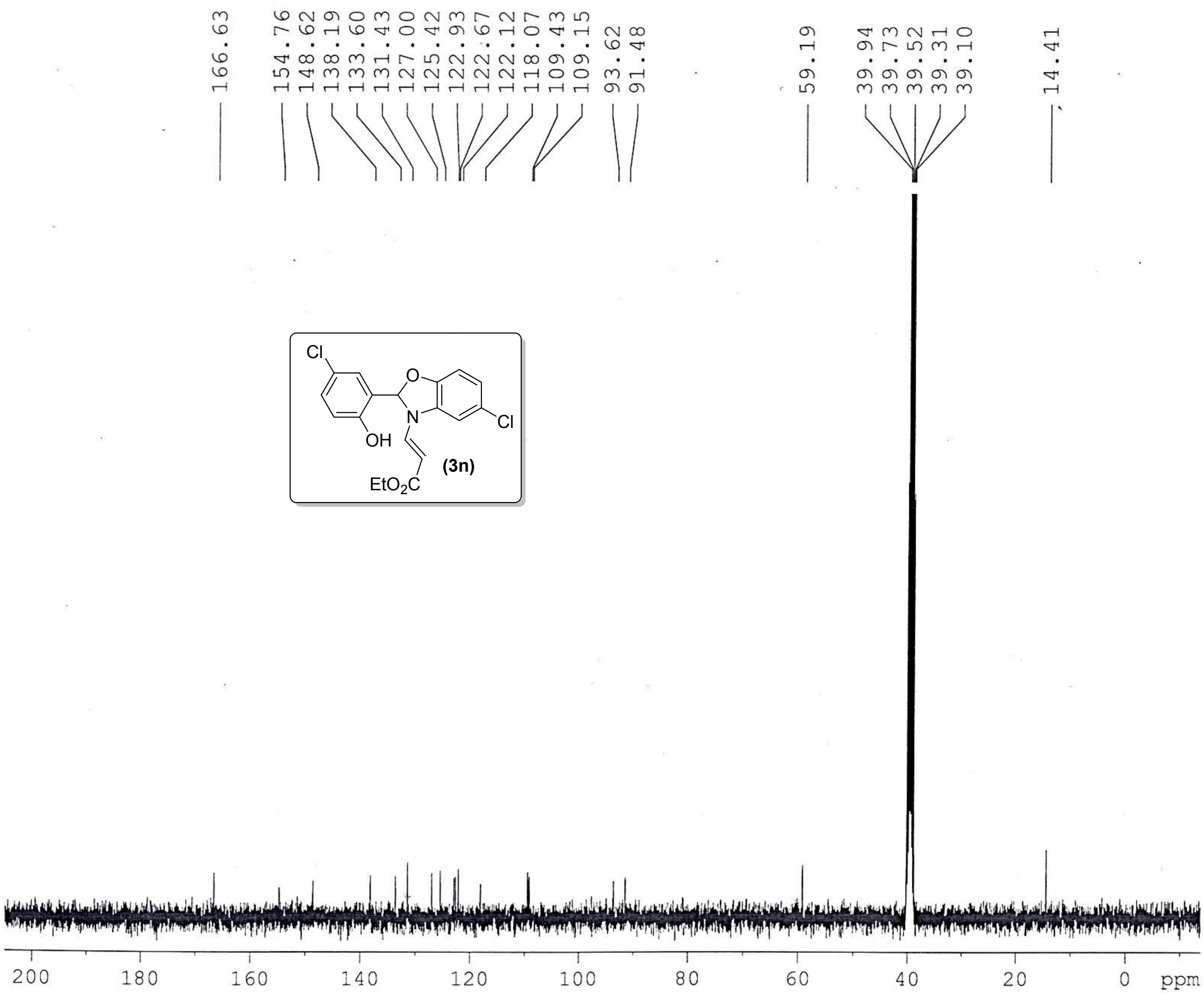

166.63
154.76
148.62
138.19
133.60
131.43
127.00
125.42
122.93
122.67
122.12
118.07
109.43
109.15
93.62
91.48
59.19
39.94
39.73
39.52
39.31
39.10
14.41
Cl
O
N
Cl
OH
EtO2C
(3n)
200
180
160
140
120
100
80
60
40
20
0
ppm

**Sample Name**	MM-169	**Position**		**Instrument Name**	Q-TOF	**User Name**	QTOF-PU\admin
**Inj Vol**	-1	**InjPosition**		**SampleType**	Sample	**IRM Calibration Status**	Success
**Data Filename**	MM-169.d	**ACQ Method**	Pondicherry Universi				

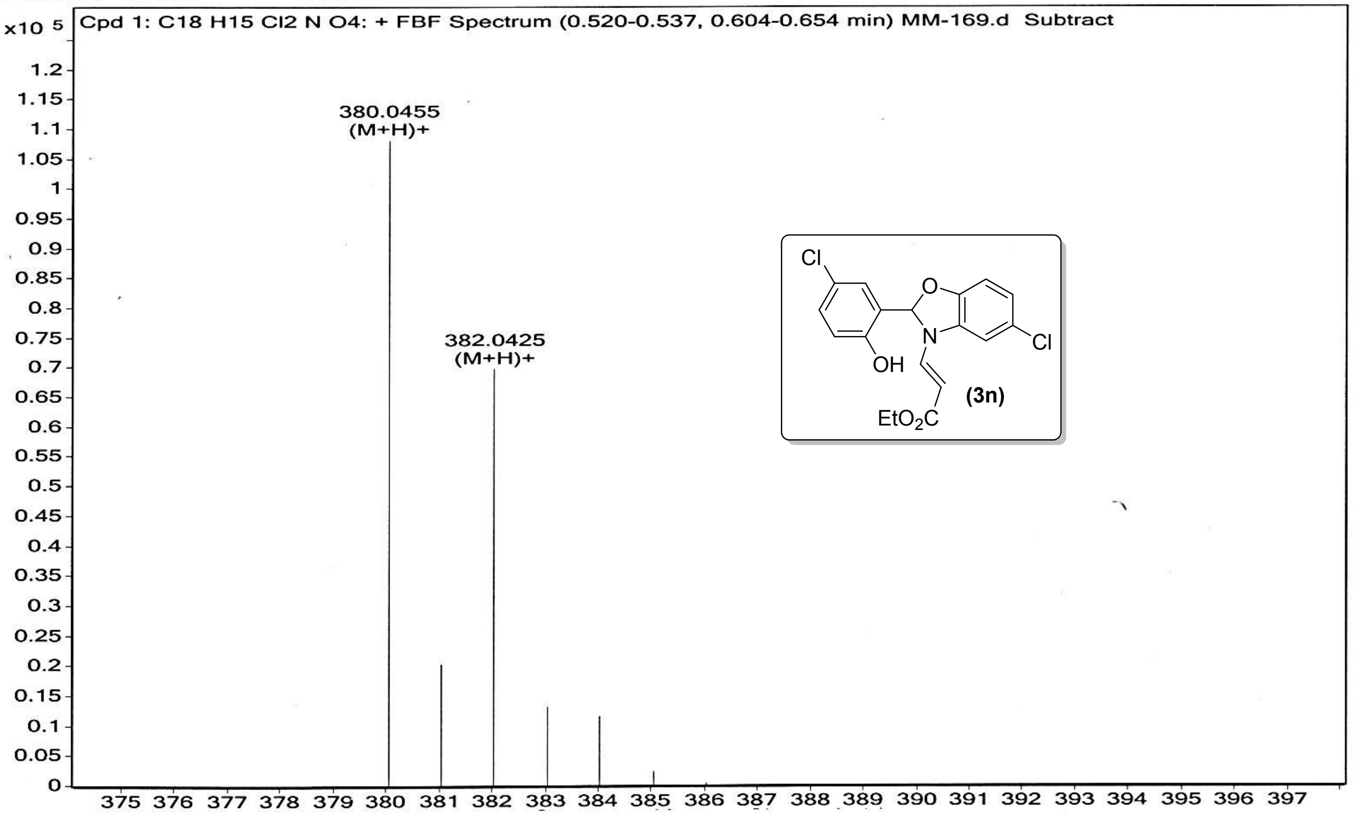

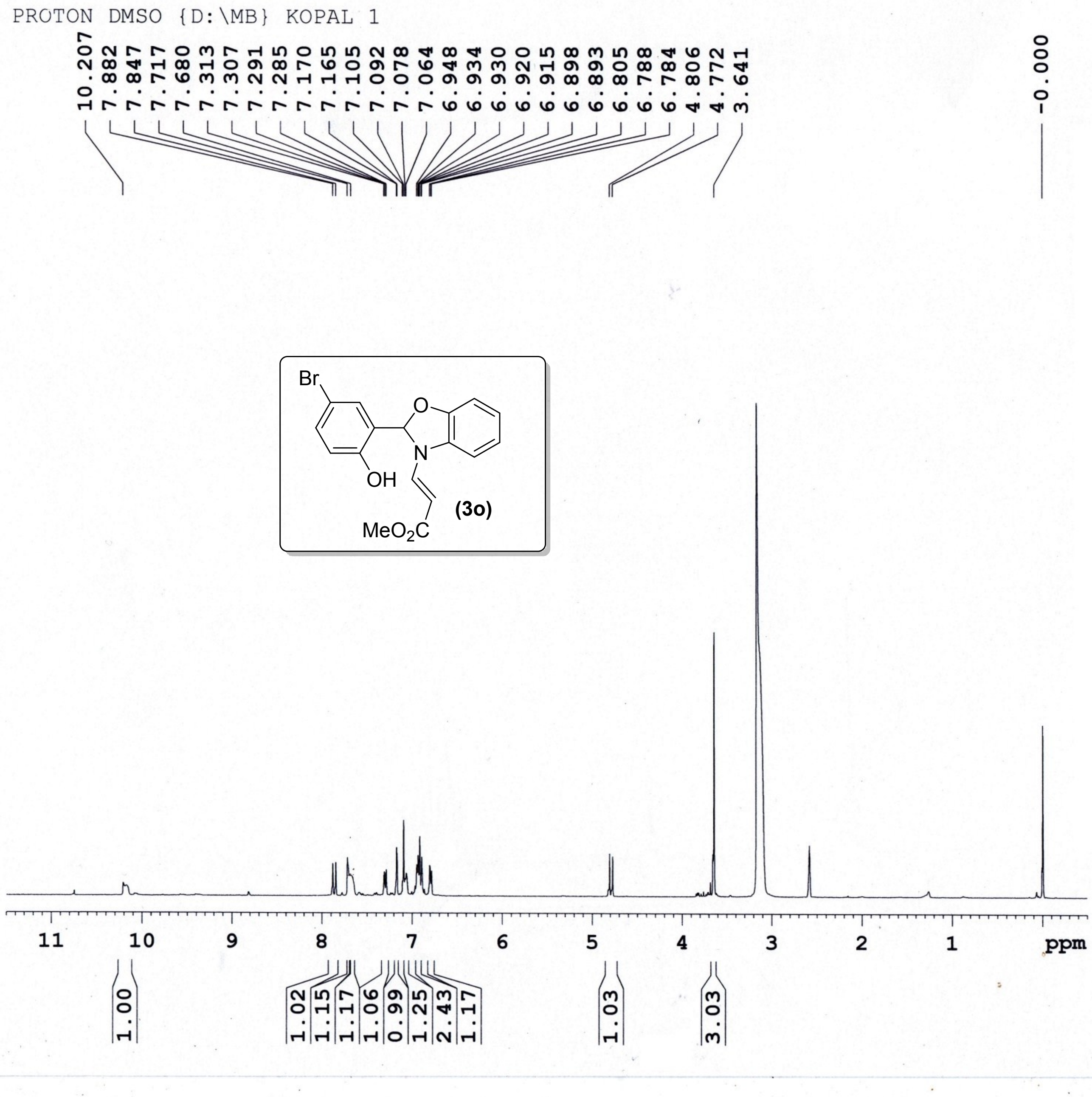
PROTON DMSO {D:\MB} KOPAL 1
10.207
7.882
7.847
7.717
7.680
7.313
7.307
7.291
7.285
7.170
7.165
7.105
7.092
7.078
7.064
6.948
6.934
6.930
6.920
6.915
6.898
6.893
6.805
6.788
6.784
4.806
4.772
3.641
-0.000
Br
O
N
OH
MeO2C
(3o)
11 10 9 8 7 6 5 4 3 2 1 ppm
1.00
1.02
1.15
1.17
1.06
0.99
1.25
2.43
1.17
1.03
3.03

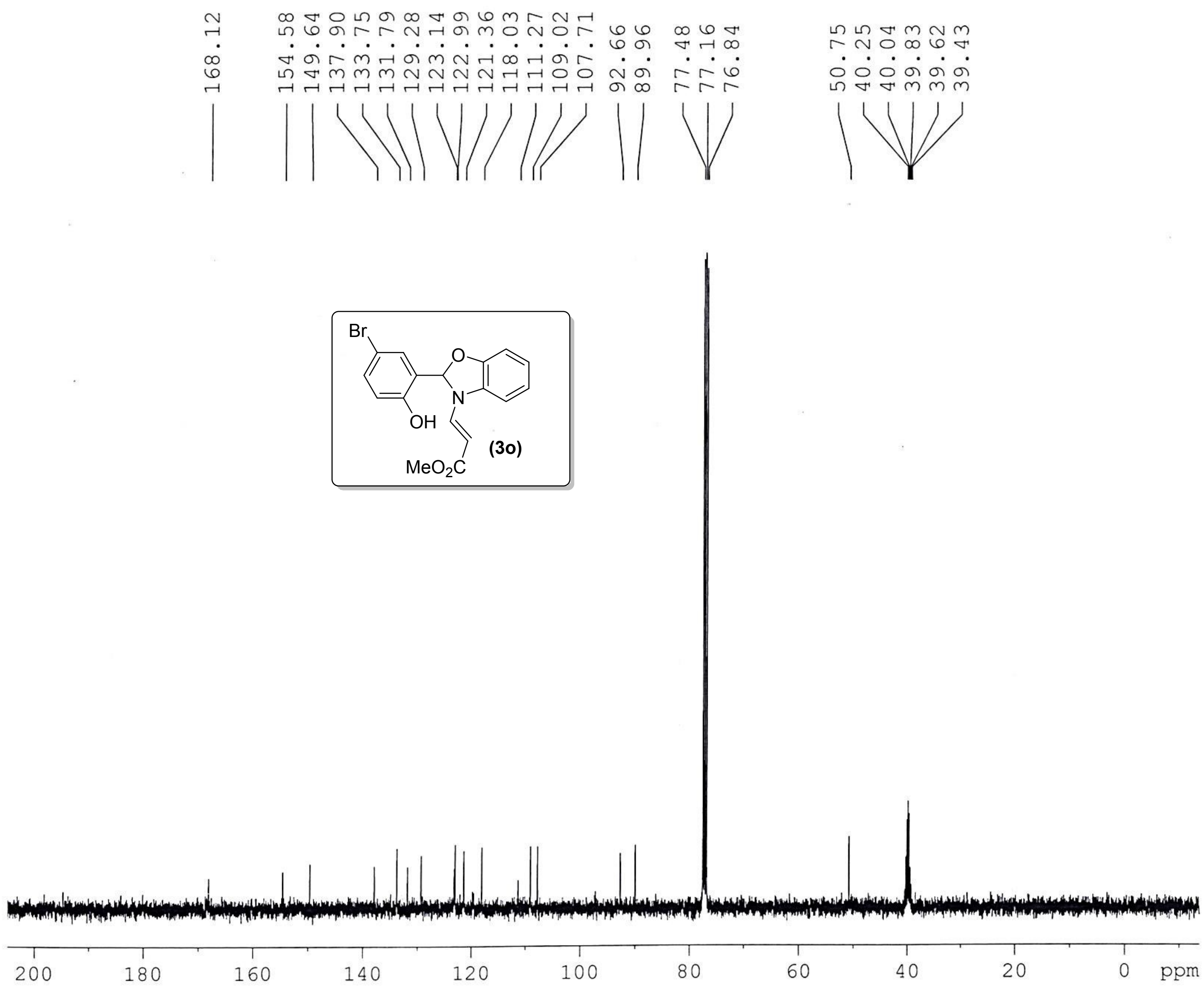

168.12
154.58
149.64
137.90
133.75
131.79
129.28
123.14
122.99
121.36
118.03
111.27
109.02
107.71
92.66
89.96
77.48
77.16
76.84
50.75
40.25
40.04
39.83
39.62
39.43
Br
O
N
OH
MeO2C
(3o)
200 180 160 140 120 100 80 60 40 20 0 ppm

**Sample Name**	MM-117	**Position**		**Instrument Name**	Q-TOF	**User Name**	QTOF-PU\admin
**Inj Vol**	-1	**InjPosition**		**SampleType**	Sample	**IRM Calibration Status**	Success
**Data Filename**	MM-117.d	**ACQ Method**	Pondicherry Universi				

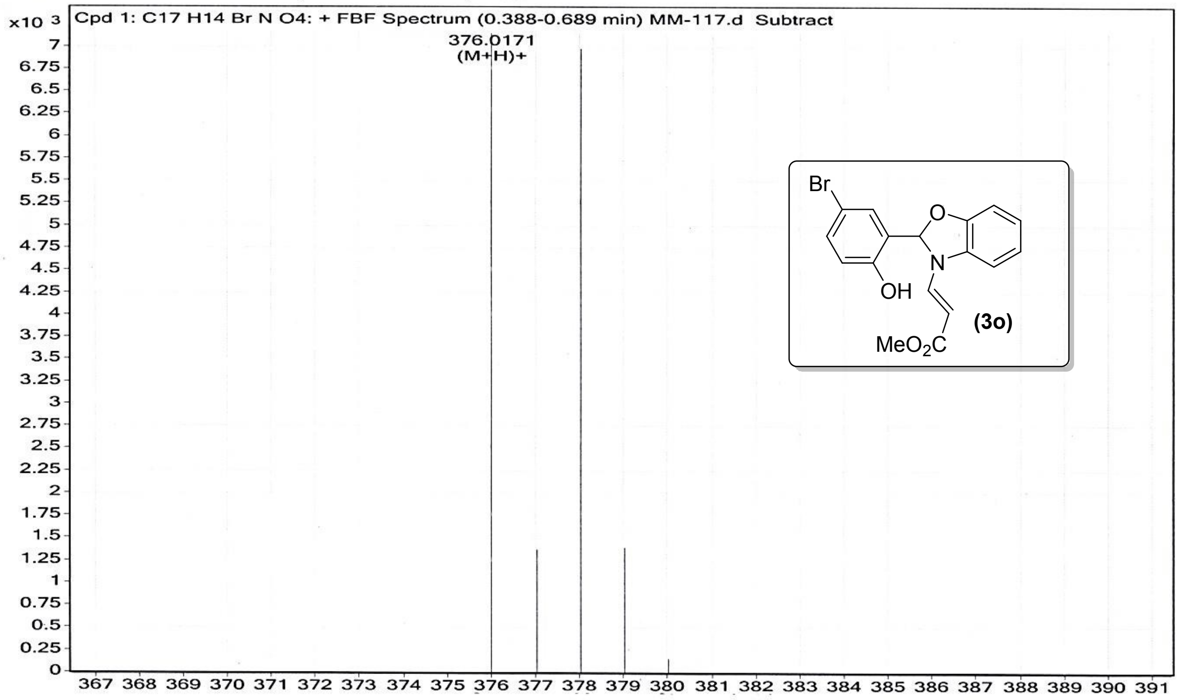

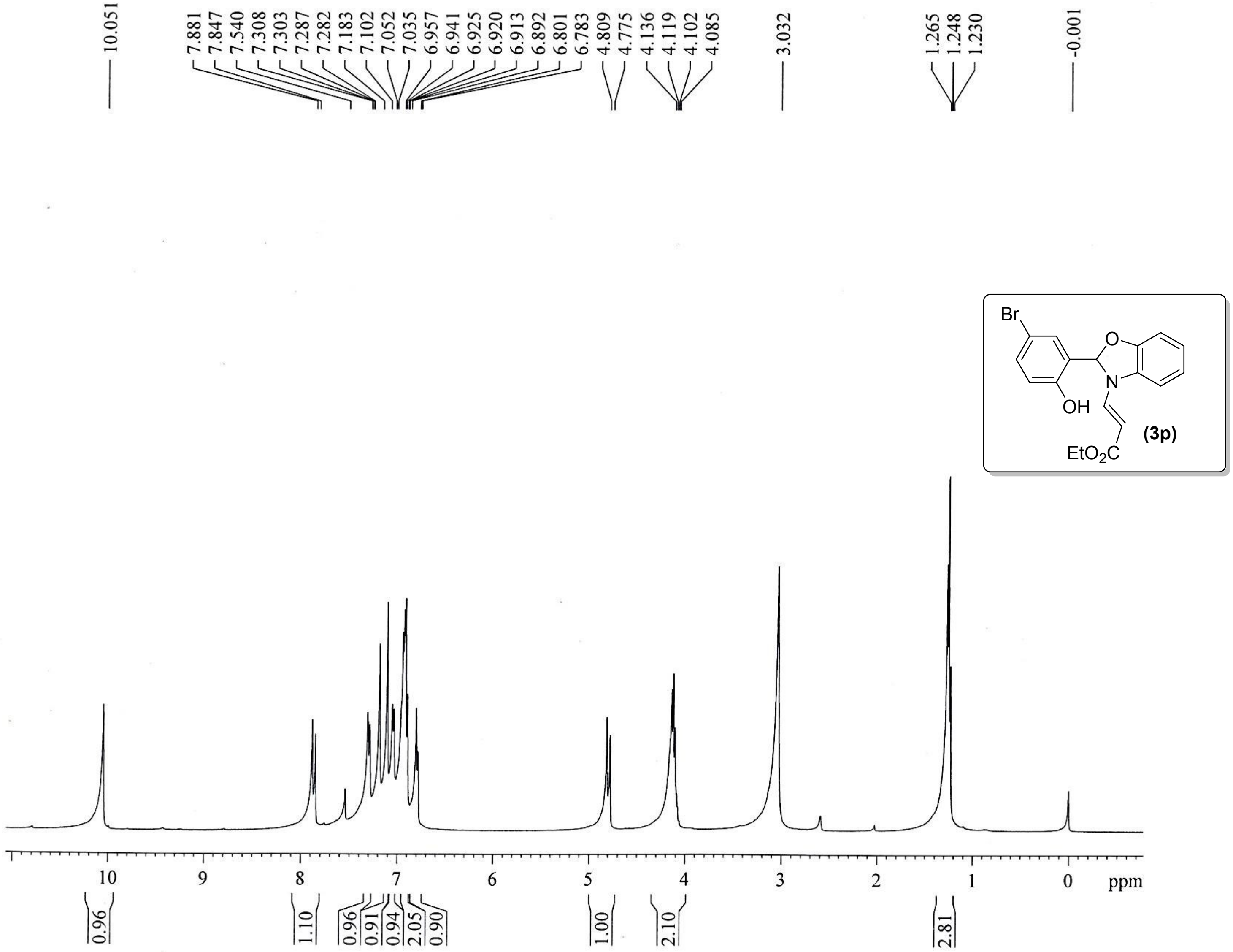
10.051
7.881 7.847 7.540 7.308 7.303 7.287 7.282 7.183 7.102 7.052 7.035 6.957 6.941 6.925 6.920 6.913 6.892 6.801 6.783
4.809 4.775
4.136 4.119 4.102 4.085
3.032
1.265 1.248 1.230
-0.001
Br
O
N
OH
EtO2C
(3p)
10 9 8 7 6 5 4 3 2 1 0 ppm
0.96 1.10 0.96 0.91 0.94 2.05 0.90 1.00 2.10 2.81

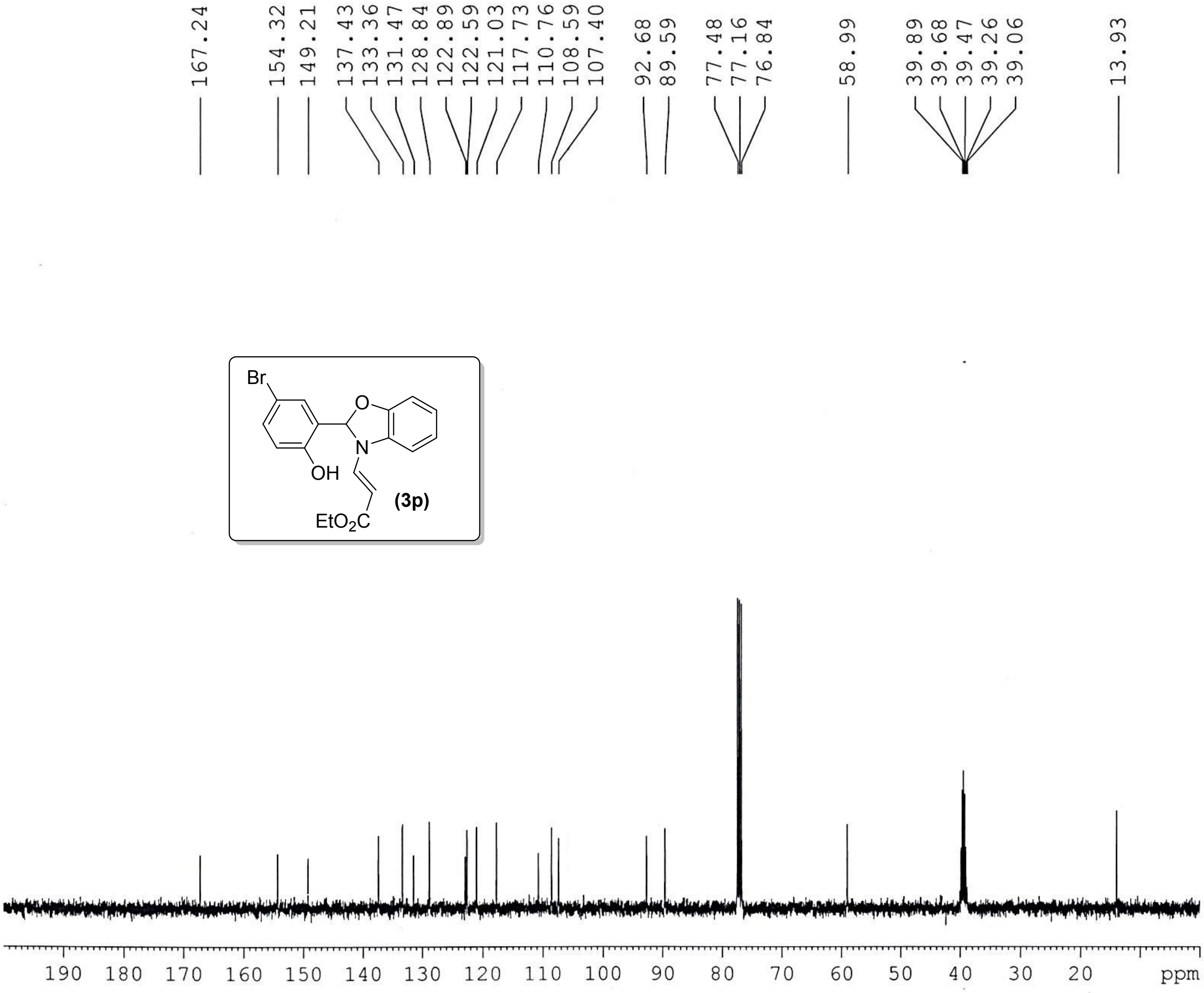
167.24
154.32
149.21
137.43
133.36
131.47
128.84
122.89
122.59
121.03
117.73
110.76
108.59
107.40
92.68
89.59
77.48
77.16
76.84
58.99
39.89
39.68
39.47
39.26
39.06
13.93
Br
O
N
OH
EtO_2C
(3p)
190 180 170 160 150 140 130 120 110 100 90 80 70 60 50 40 30 20 ppm

**Sample Name**	MM-104	**Position**		**Instrument Name**	Q-TOF	**User Name**	QTOF-PU\admin
**Inj Vol**	-1	**InjPosition**		**SampleType**	Sample	**IRM Calibration Status**	Success
**Data Filename**	MM-104.d	**ACQ Method**	Pondicherry Universi				

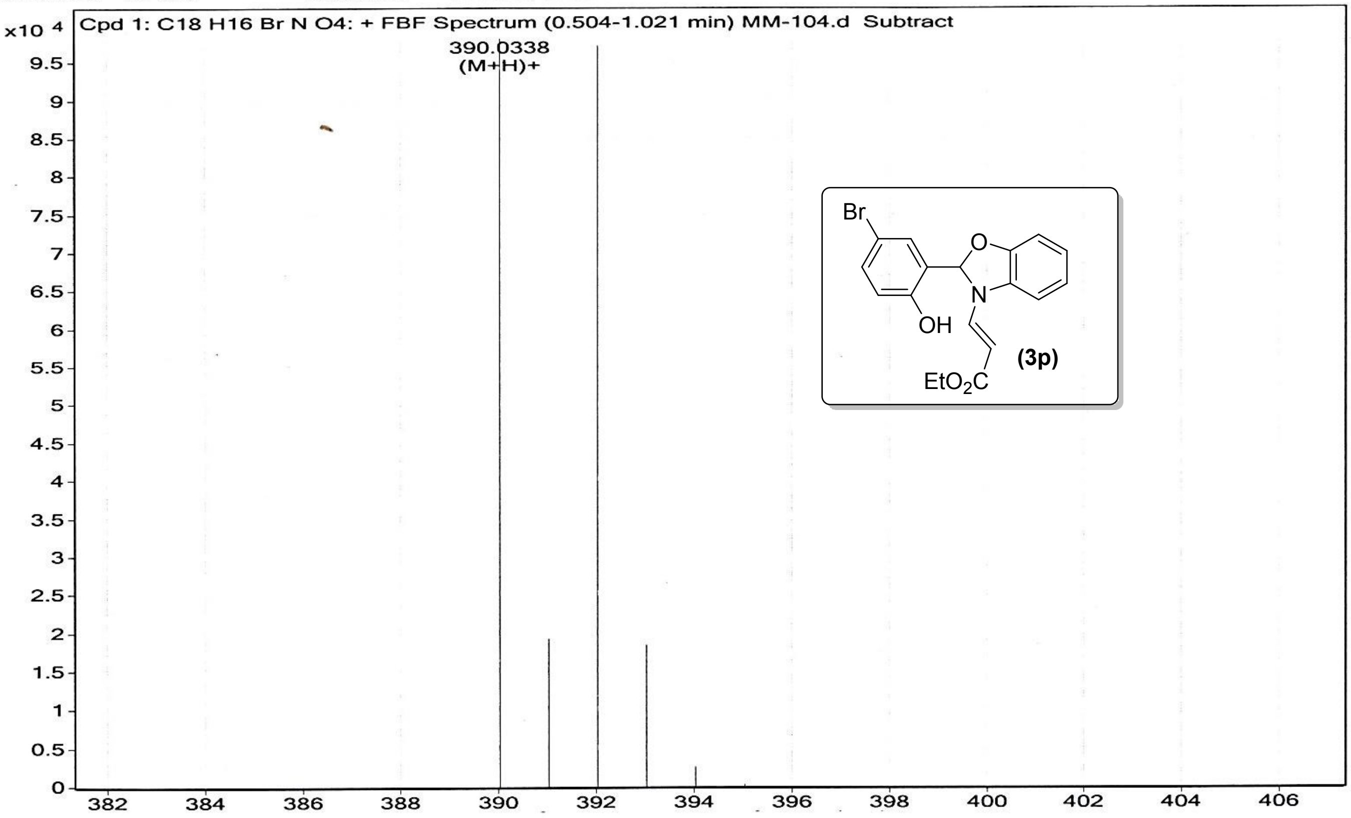

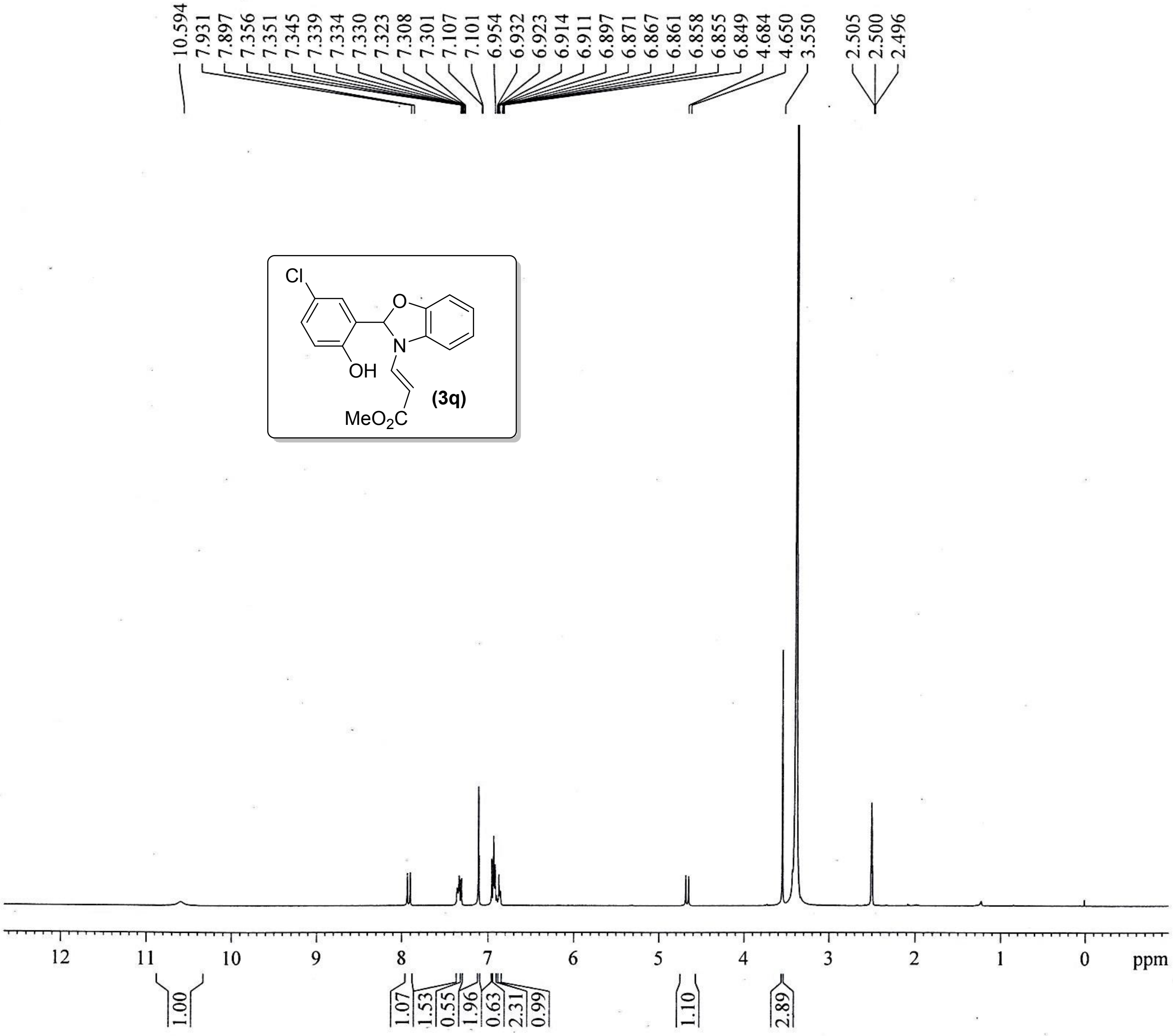
10.594
7.931
7.897
7.356
7.351
7.345
7.339
7.334
7.330
7.323
7.308
7.301
7.107
7.101
6.954
6.932
6.923
6.914
6.911
6.897
6.871
6.867
6.861
6.858
6.855
6.849
4.684
4.650
3.550
2.505
2.500
2.496
Cl
O
N
OH
MeO2C
(3q)
12
11
10
9
8
7
6
5
4
3
2
1
0
ppm
1.00
1.07
1.53
0.55
1.96
0.63
2.31
0.99
1.10
2.89

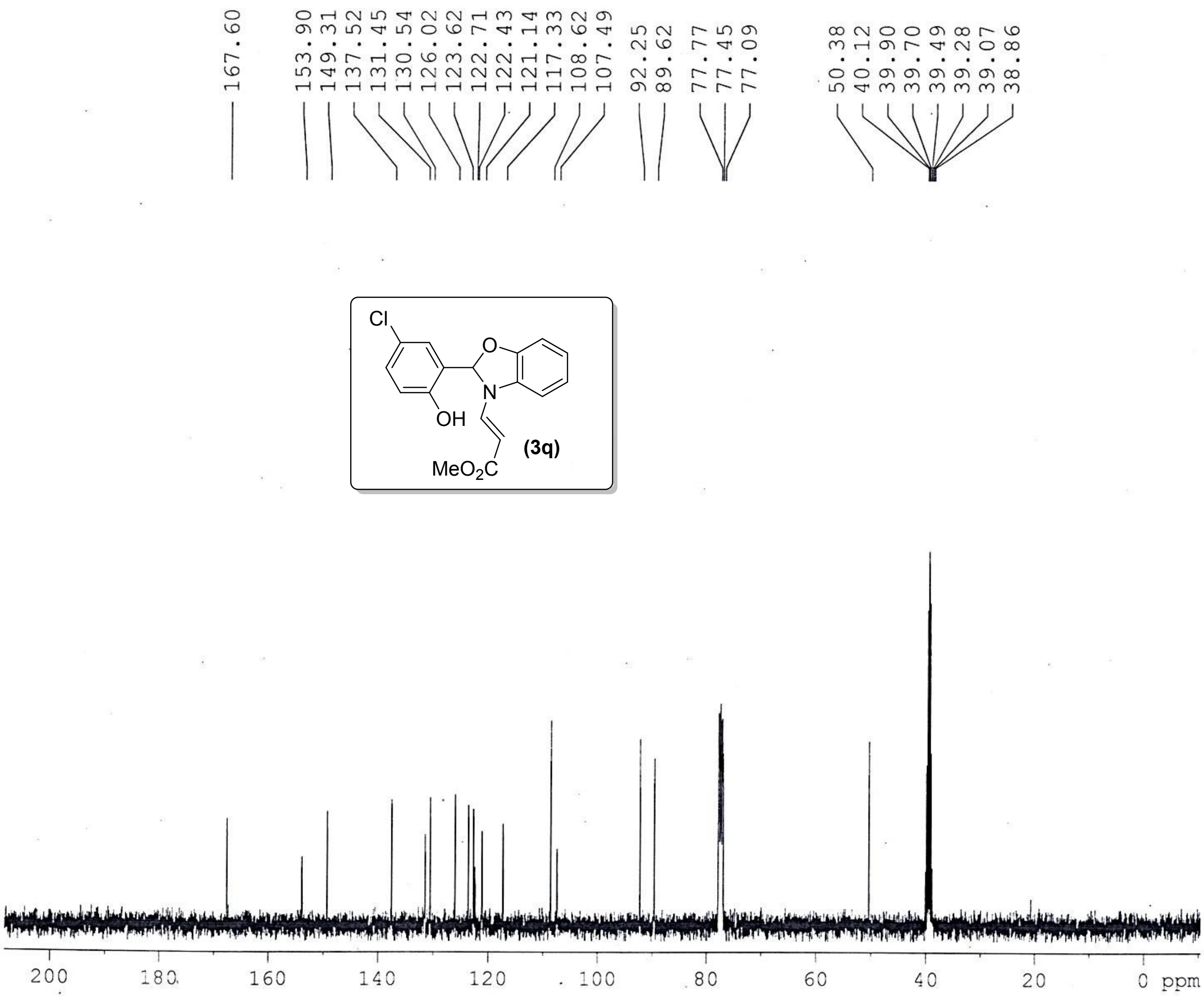
167.60
153.90
149.31
137.52
131.45
130.54
126.02
123.62
122.71
122.43
121.14
117.33
108.62
107.49
92.25
89.62
77.77
77.45
77.09
50.38
40.12
39.90
39.70
39.49
39.28
39.07
38.86
Cl
O
N
OH
MeO_2C
(3q)
200
180
160
140
120
100
80
60
40
20
0 ppm

**Sample Name**	MM-166	**Position**		**Instrument Name**	Q-TOF	**User Name**	QTOF-PU\admin
**Inj Vol**	-1	**InjPosition**		**SampleType**	Sample	**IRM Calibration Status**	Success
**Data Filename**	MM-166.d	**ACQ Method**	Pondicherry Universi				

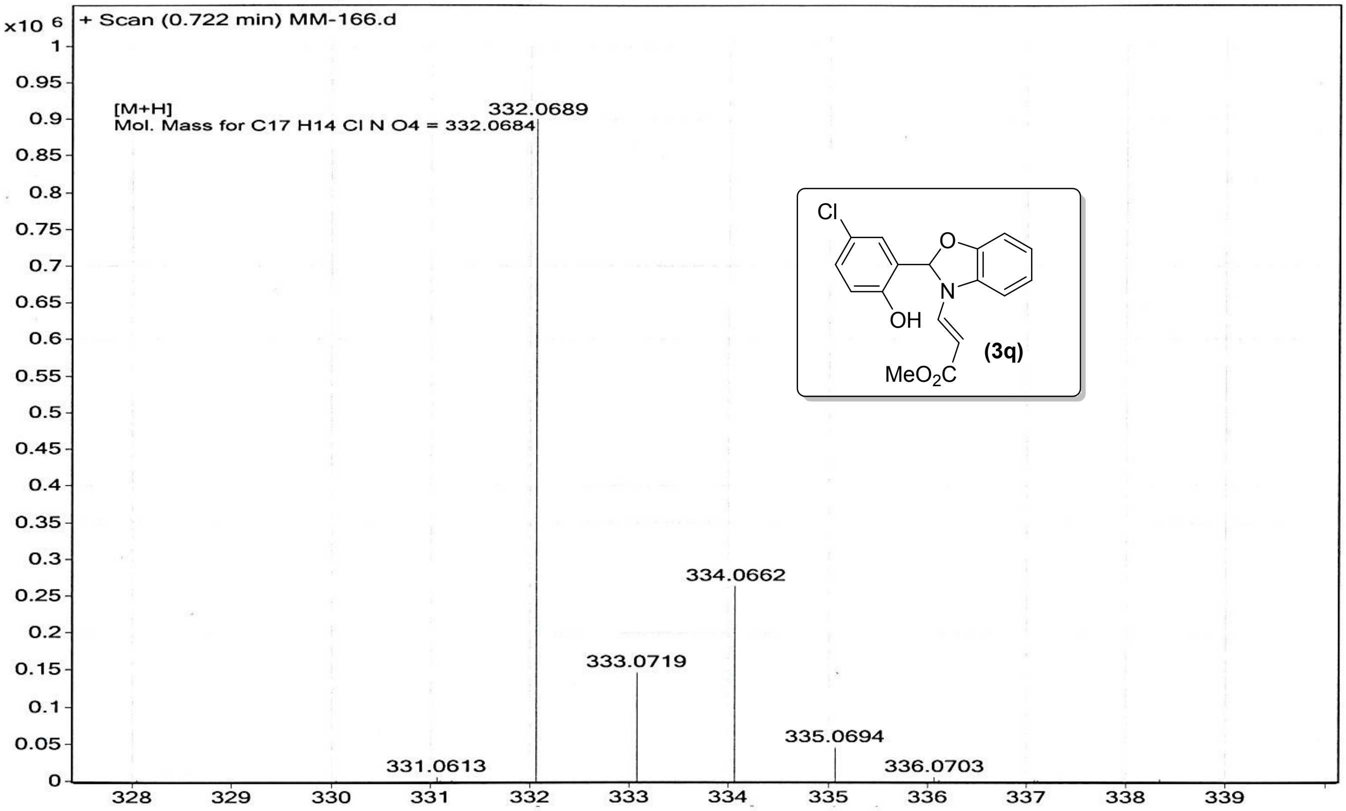

PROTON CDCl3 {D:\MB} KOPAL 1

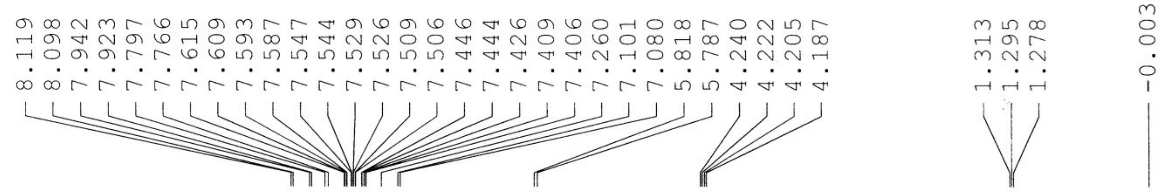

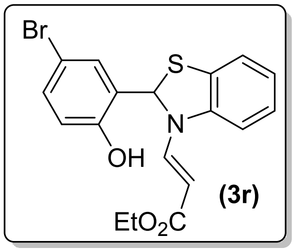

(3r)

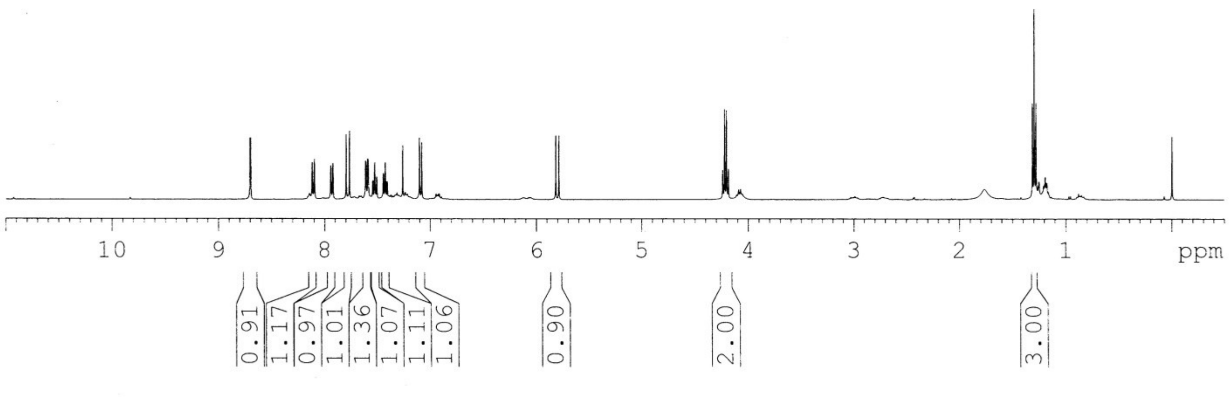

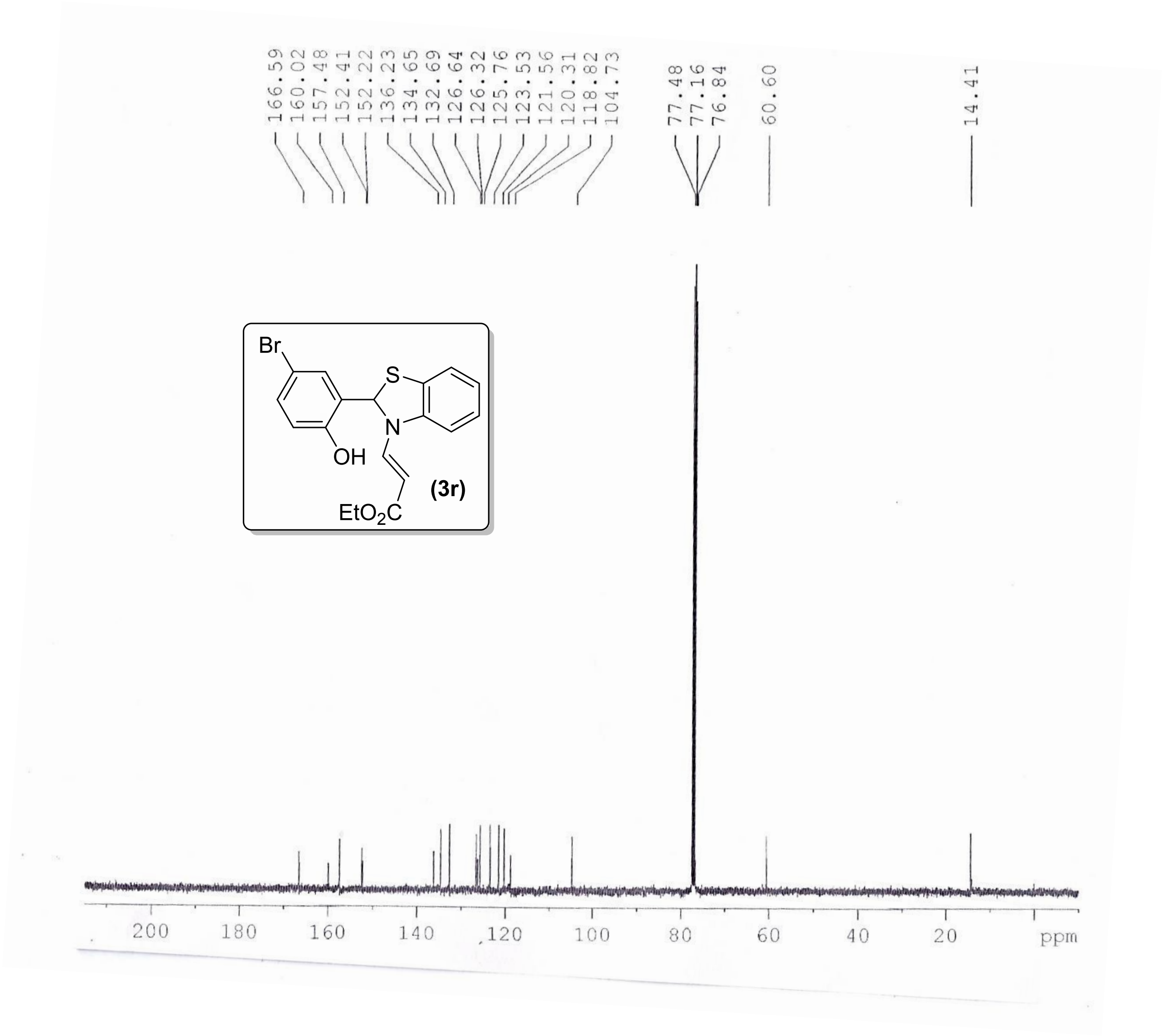

166.59
160.02
157.48
152.41
152.22
136.23
134.65
132.69
126.64
126.32
125.76
123.53
121.56
120.31
118.82
104.73
77.48
77.16
76.84
60.60
14.41
Br
S
N
OH
EtO2C
(3r)
200
180
160
140
120
100
80
60
40
20
ppm

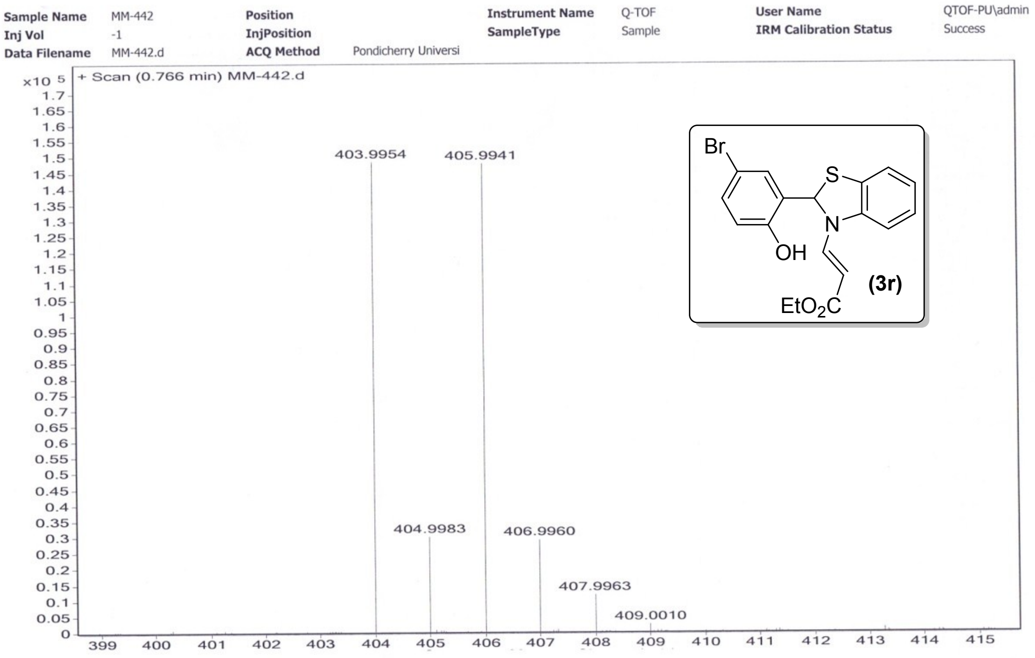
Sample Name MM-442
Inj Vol -1
Data Filename MM-442.d
Position
InjPosition
ACQ Method Pondicherry Universi
Instrument Name Q-TOF
SampleType Sample
User Name QTOF-PU\admin
IRM Calibration Status Success
x10 5
+ Scan (0.766 min) MM-442.d
403.9954
405.9941
404.9983
406.9960
407.9963
409.0010
Br
S
N
OH
EtO2C
(3r)

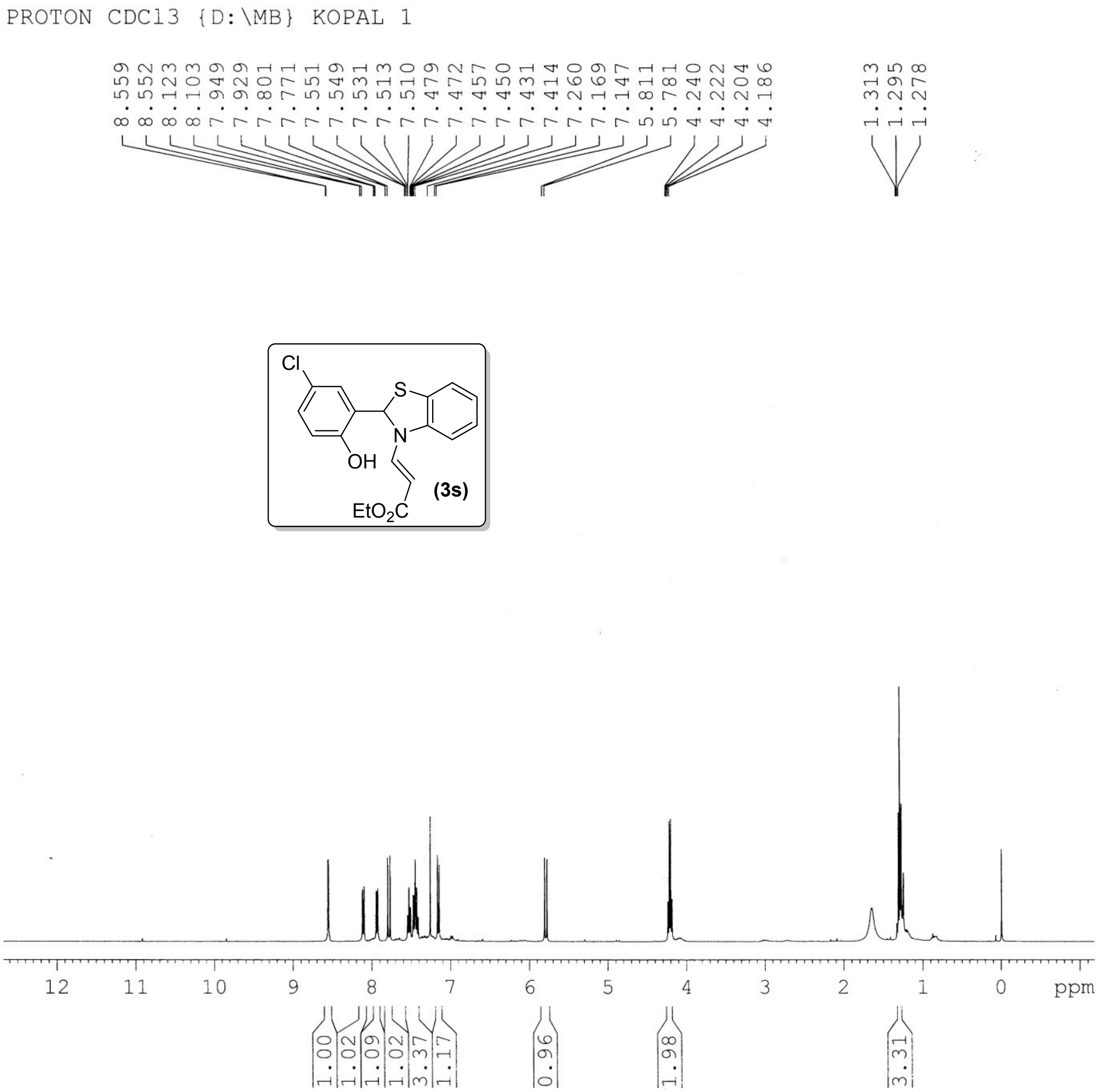
PROTON CDCl3 {D:\MB} KOPAL 1
8.559
8.552
8.123
8.103
7.949
7.929
7.801
7.771
7.551
7.549
7.531
7.513
7.510
7.479
7.472
7.457
7.450
7.431
7.414
7.260
7.169
7.147
5.811
5.781
4.240
4.222
4.204
4.186
1.313
1.295
1.278
Cl
S
N
OH
EtO2C
(3s)
12
11
10
9
8
7
6
5
4
3
2
1
0
ppm
1.00
1.02
1.09
1.02
3.37
1.17
0.96
1.98
3.31

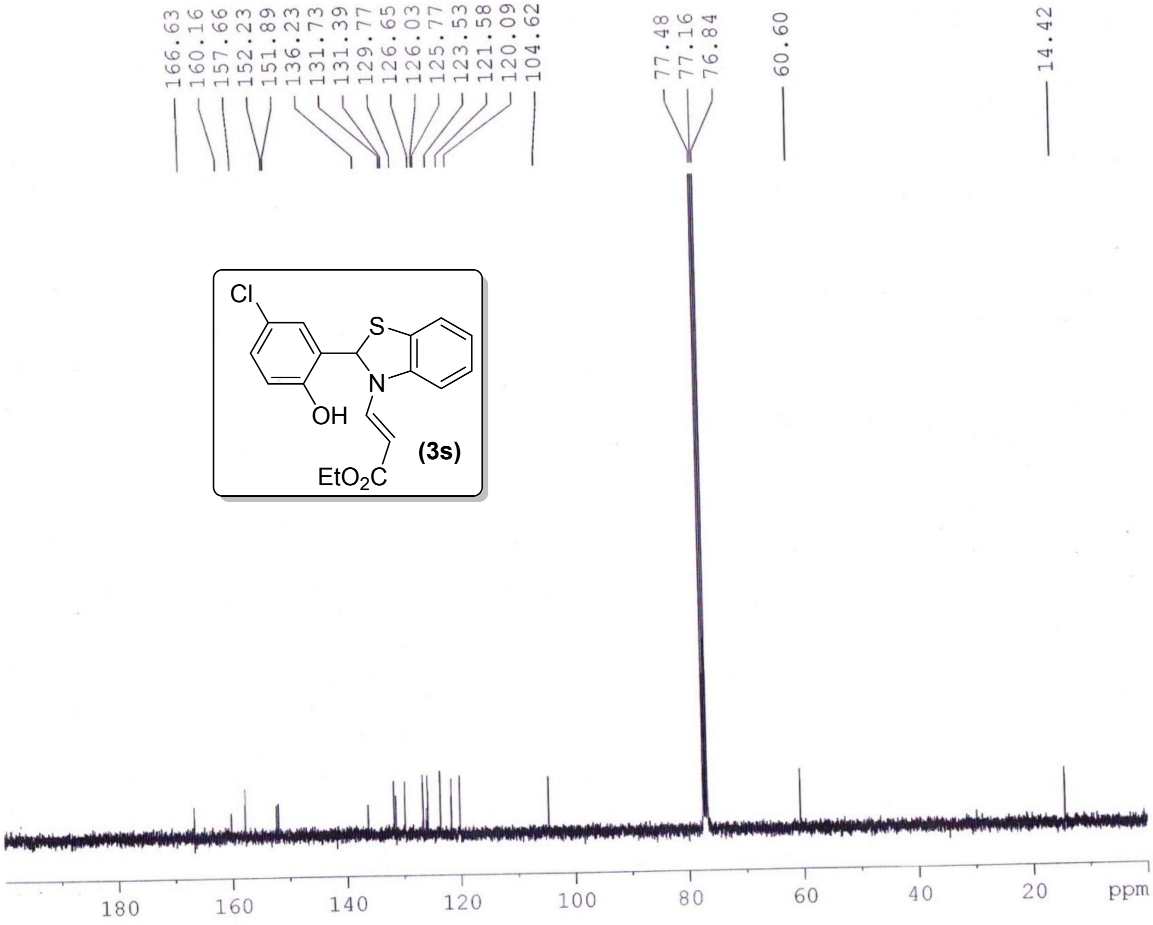
166.63
160.16
157.66
152.23
151.89
136.23
131.73
131.39
129.77
126.65
126.03
125.77
123.53
121.58
120.09
104.62
77.48
77.16
76.84
60.60
14.42
Cl
S
N
OH
EtO2C
(3s)
180
160
140
120
100
80
60
40
20
ppm

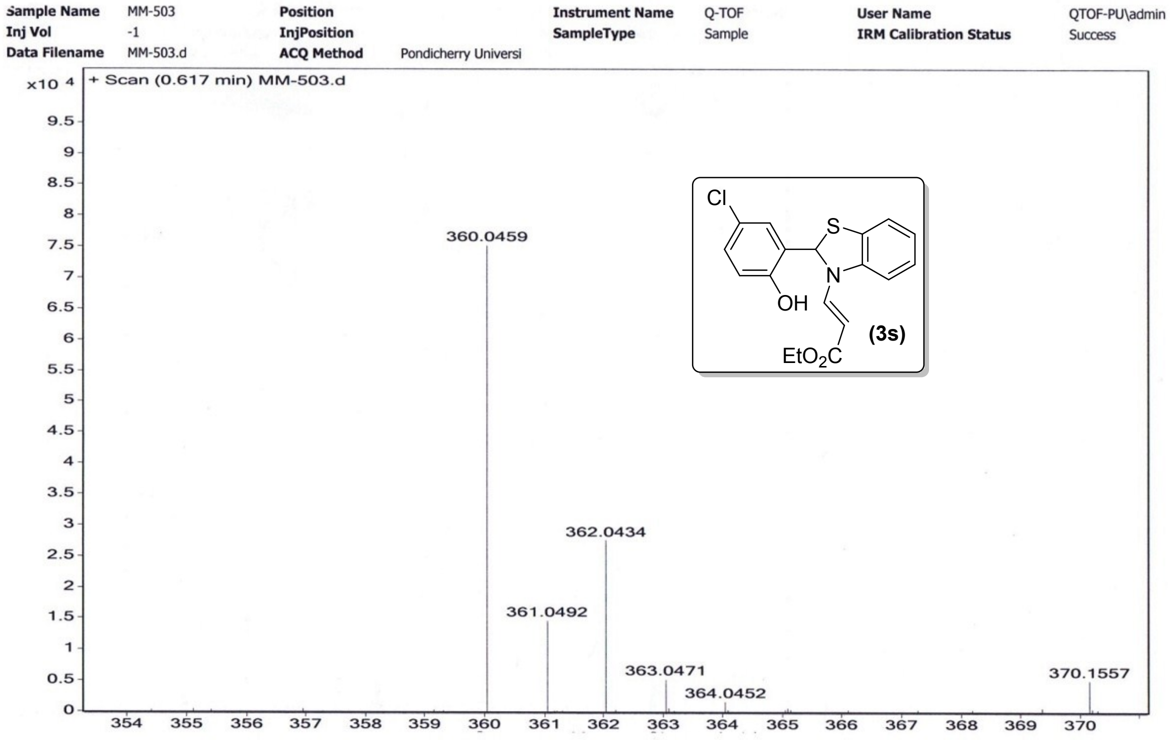
Sample Name MM-503
Inj Vol -1
Data Filename MM-503.d
Position
InjPosition
ACQ Method Pondicherry Universi
Instrument Name Q-TOF
SampleType Sample
User Name QTOF-PU\admin
IRM Calibration Status Success
x10 4 + Scan (0.617 min) MM-503.d
360.0459
361.0492
362.0434
363.0471
364.0452
370.1557
Cl
S
N
OH
EtO2C
(3s)

PROTON DMSO {D:\MB} KOPAL 1

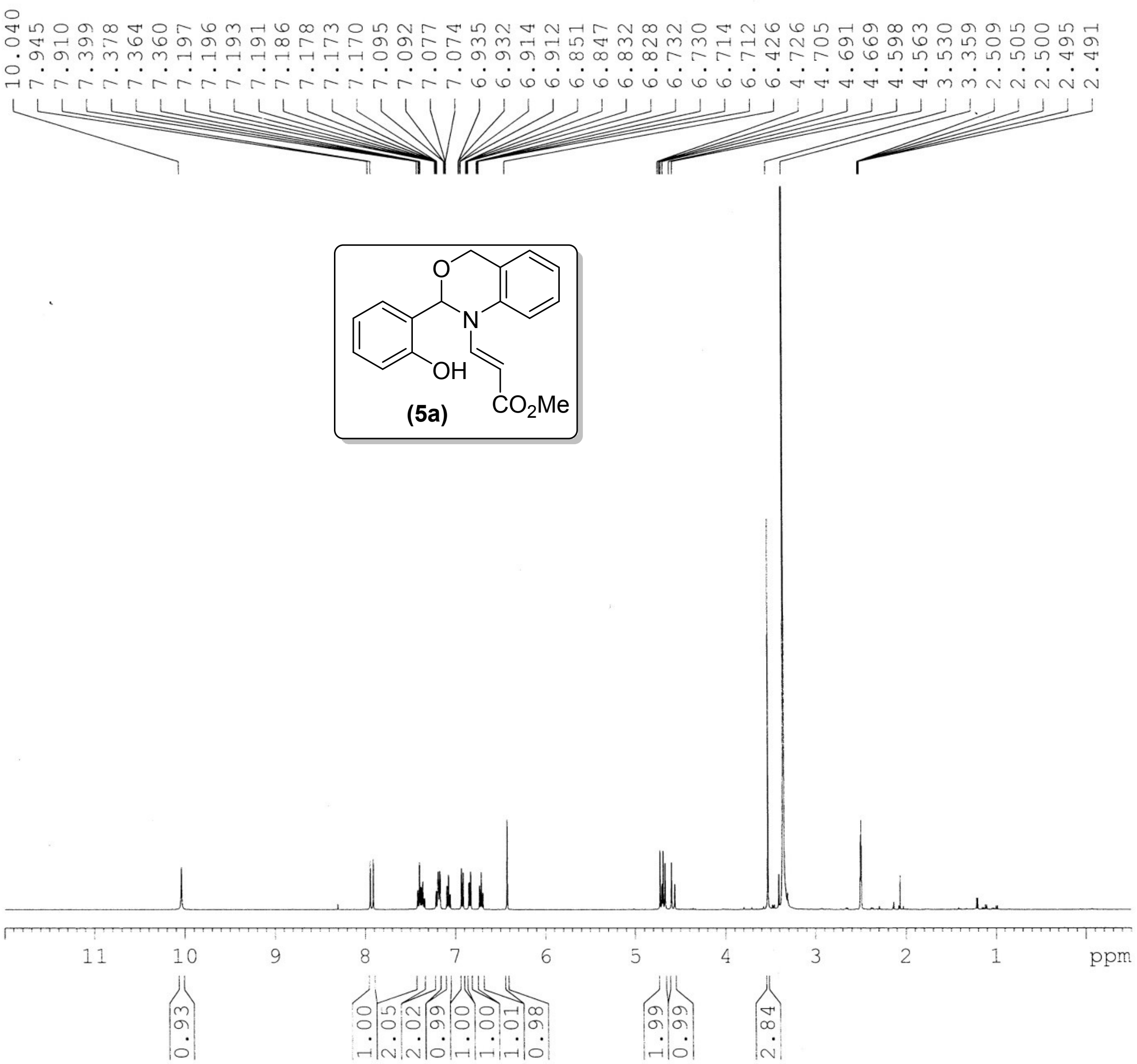

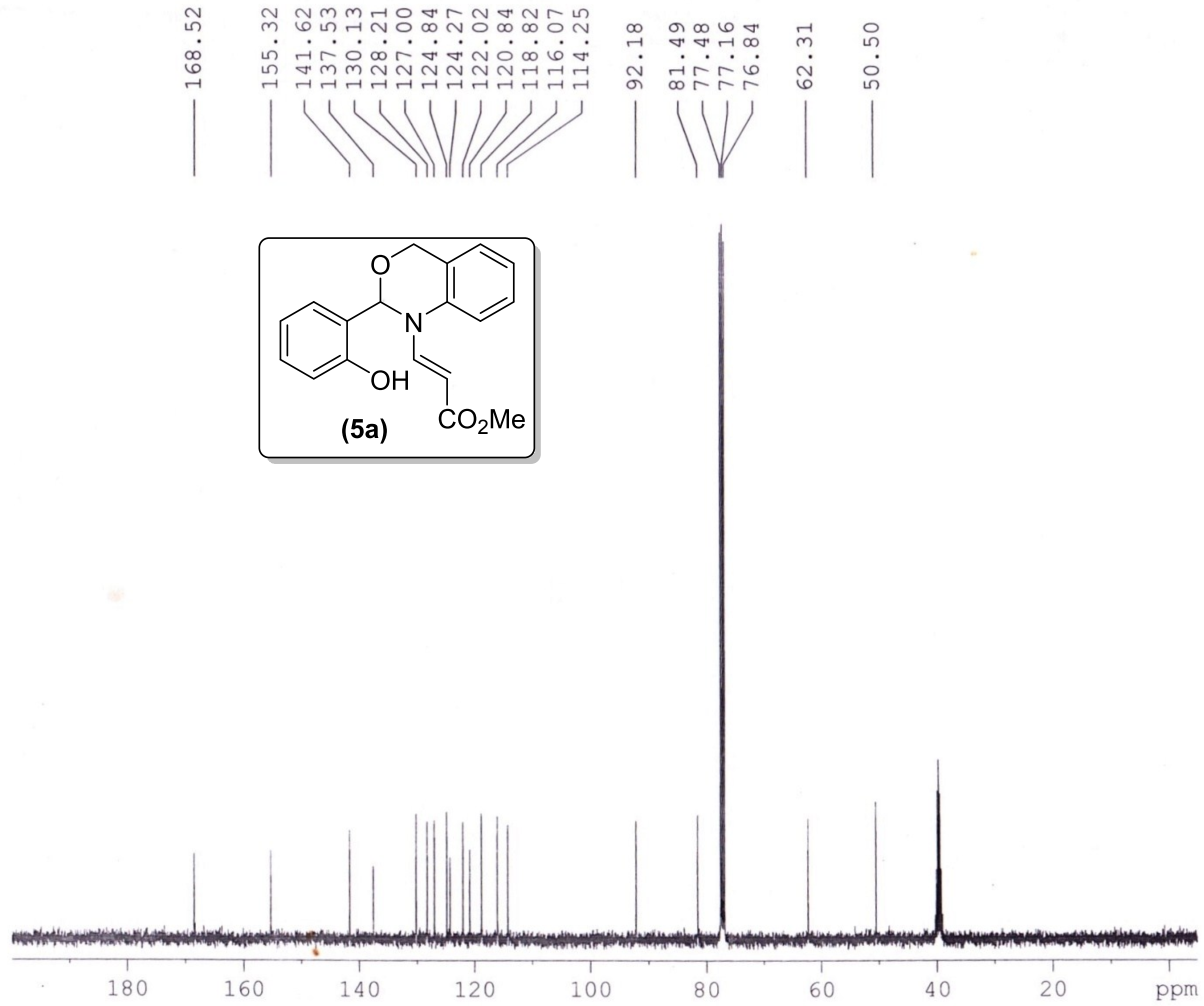
168.52
155.32
141.62
137.53
130.13
128.21
127.00
124.84
124.27
122.02
120.84
118.82
116.07
114.25
92.18
81.49
77.48
77.16
76.84
62.31
50.50
O
N
OH
CO2Me
(5a)
180
160
140
120
100
80
60
40
20
ppm

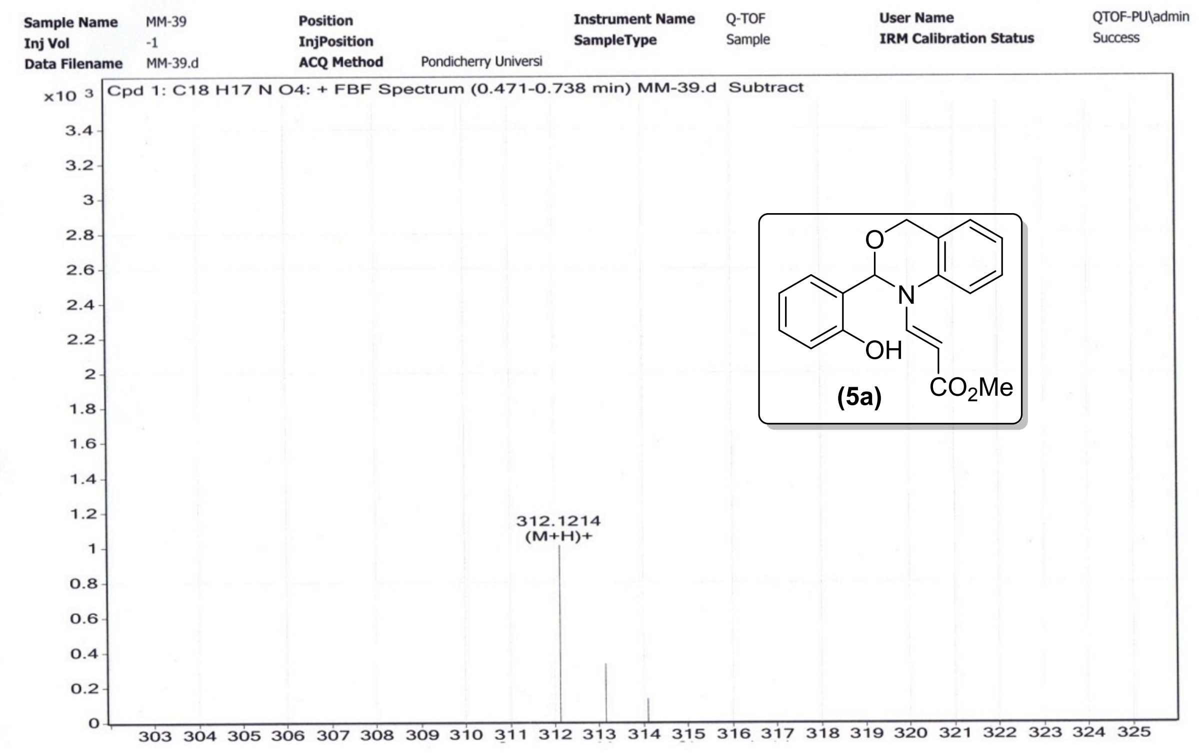
Sample Name MM-39
Inj Vol -1
Data Filename MM-39.d
Position
InjPosition
ACQ Method Pondicherry Universi
Instrument Name Q-TOF
SampleType Sample
User Name QTOF-PU\admin
IRM Calibration Status Success
x10 3
Cpd 1: C18 H17 N O4: + FBF Spectrum (0.471-0.738 min) MM-39.d Subtract
3.4
3.2
3
2.8
2.6
2.4
2.2
2
1.8
1.6
1.4
1.2
1
0.8
0.6
0.4
0.2
0
312.1214
(M+H)+
303 304 305 306 307 308 309 310 311 312 313 314 315 316 317 318 319 320 321 322 323 324 325
O
N
OH
CO2Me
(5a)

PROTON DMSO {D:\MB} KOPAL 1

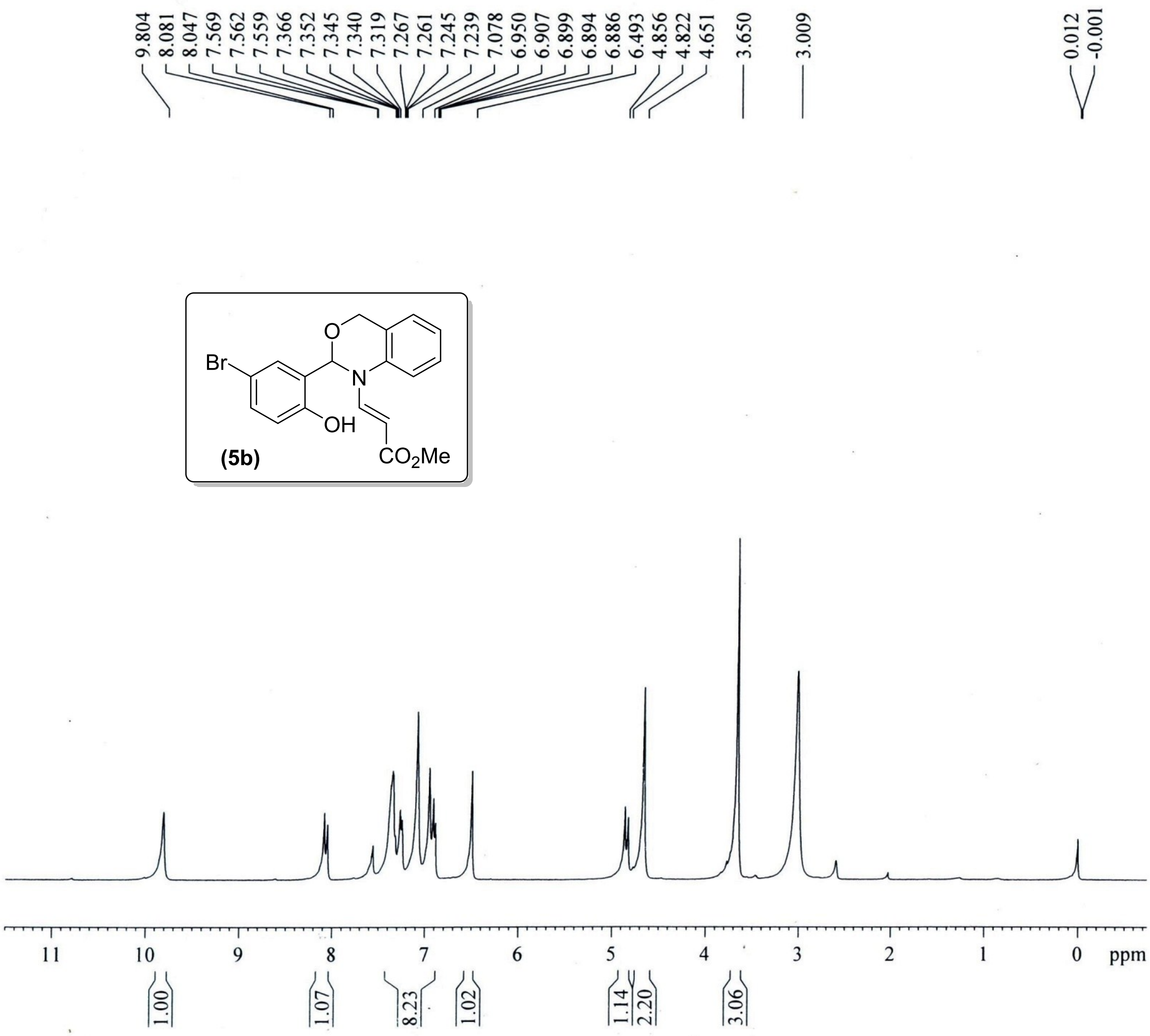

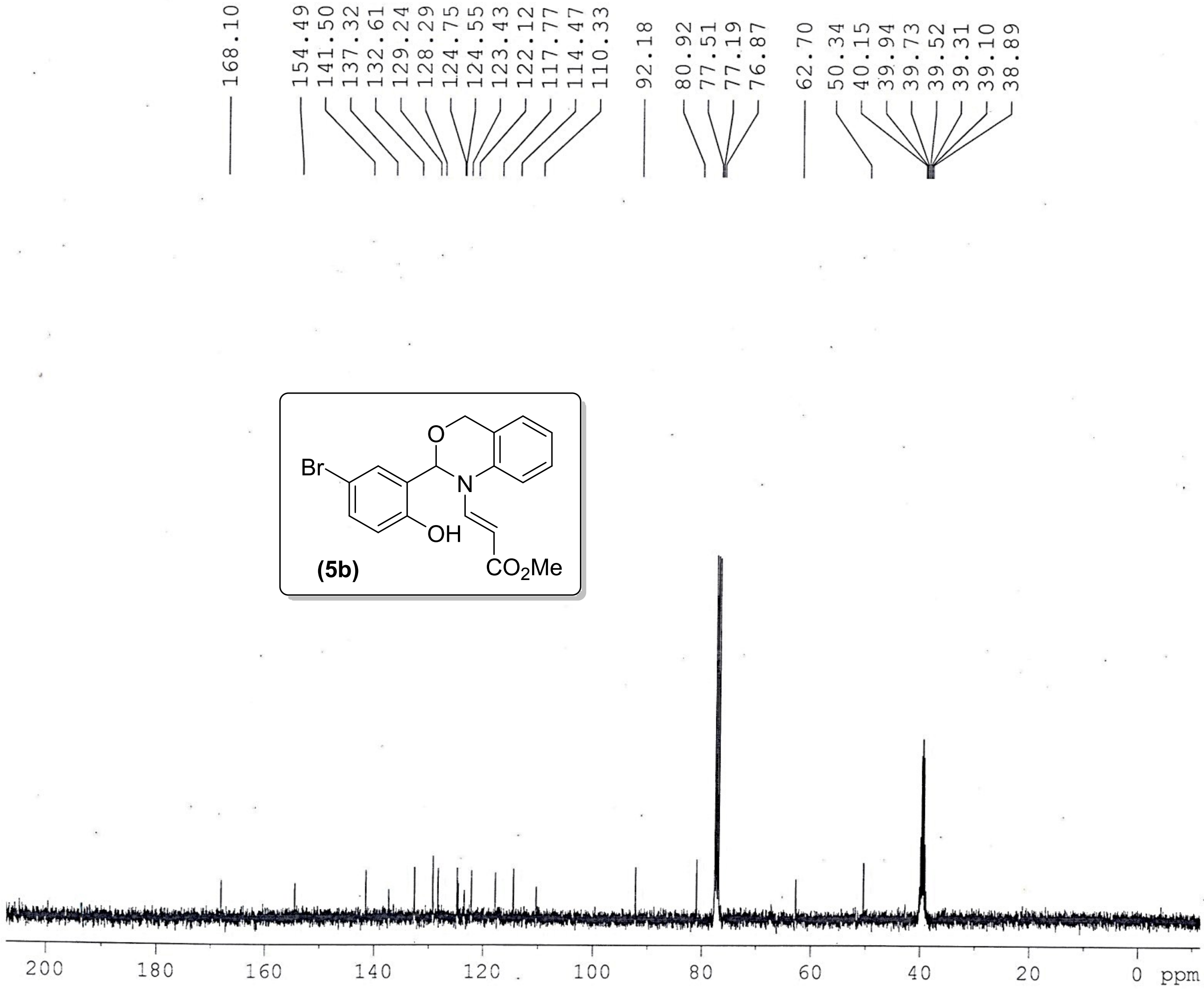
168.10
154.49
141.50
137.32
132.61
129.24
128.29
124.75
124.55
123.43
122.12
117.77
114.47
110.33
92.18
80.92
77.51
77.19
76.87
62.70
50.34
40.15
39.94
39.73
39.52
39.31
39.10
38.89
Br
O
N
OH
CO_2Me
(5b)
200
180
160
140
120
100
80
60
40
20
0 ppm

**Sample Name**	MM-118	**Position**		**Instrument Name**	Q-TOF	**User Name**	QTOF-PU\admin
**Inj Vol**	-1	**InjPosition**		**SampleType**	Sample	**IRM Calibration Status**	Success
**Data Filename**	MM-118.d	**ACQ Method**	Pondicherry Universi				

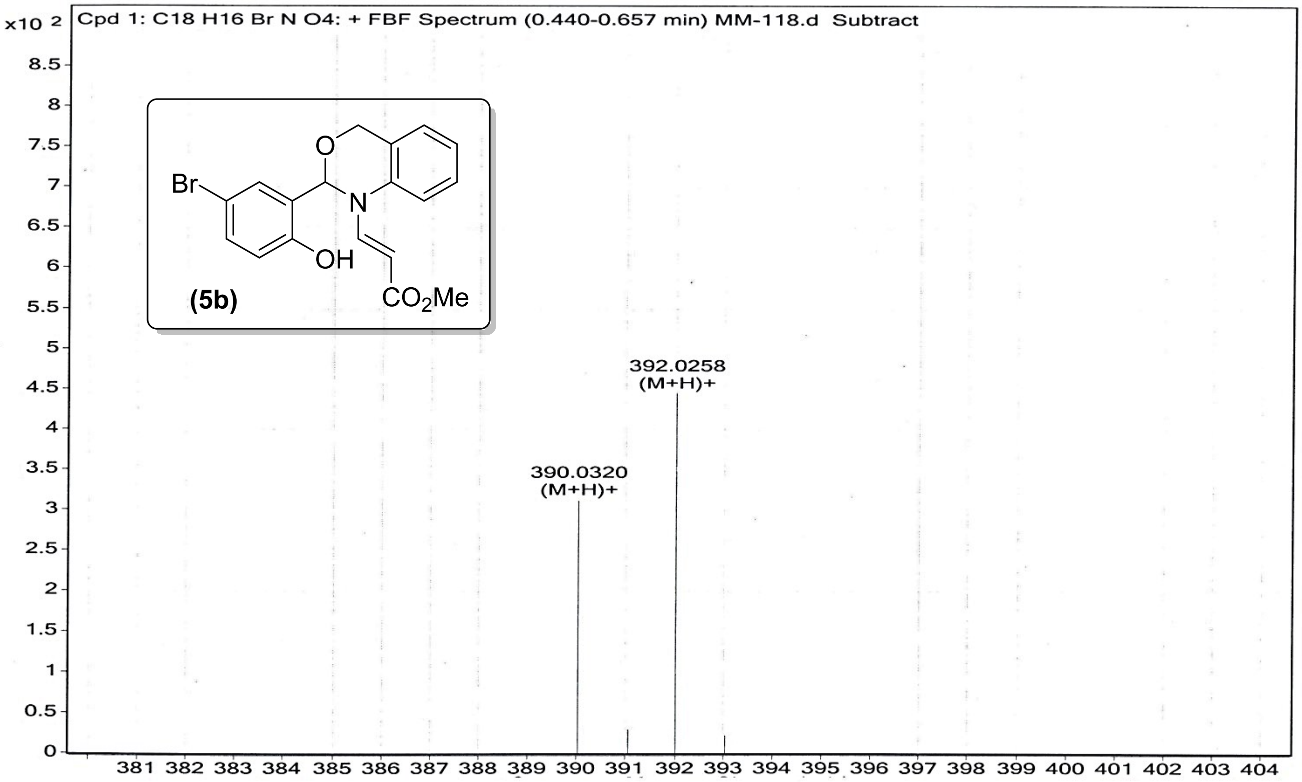

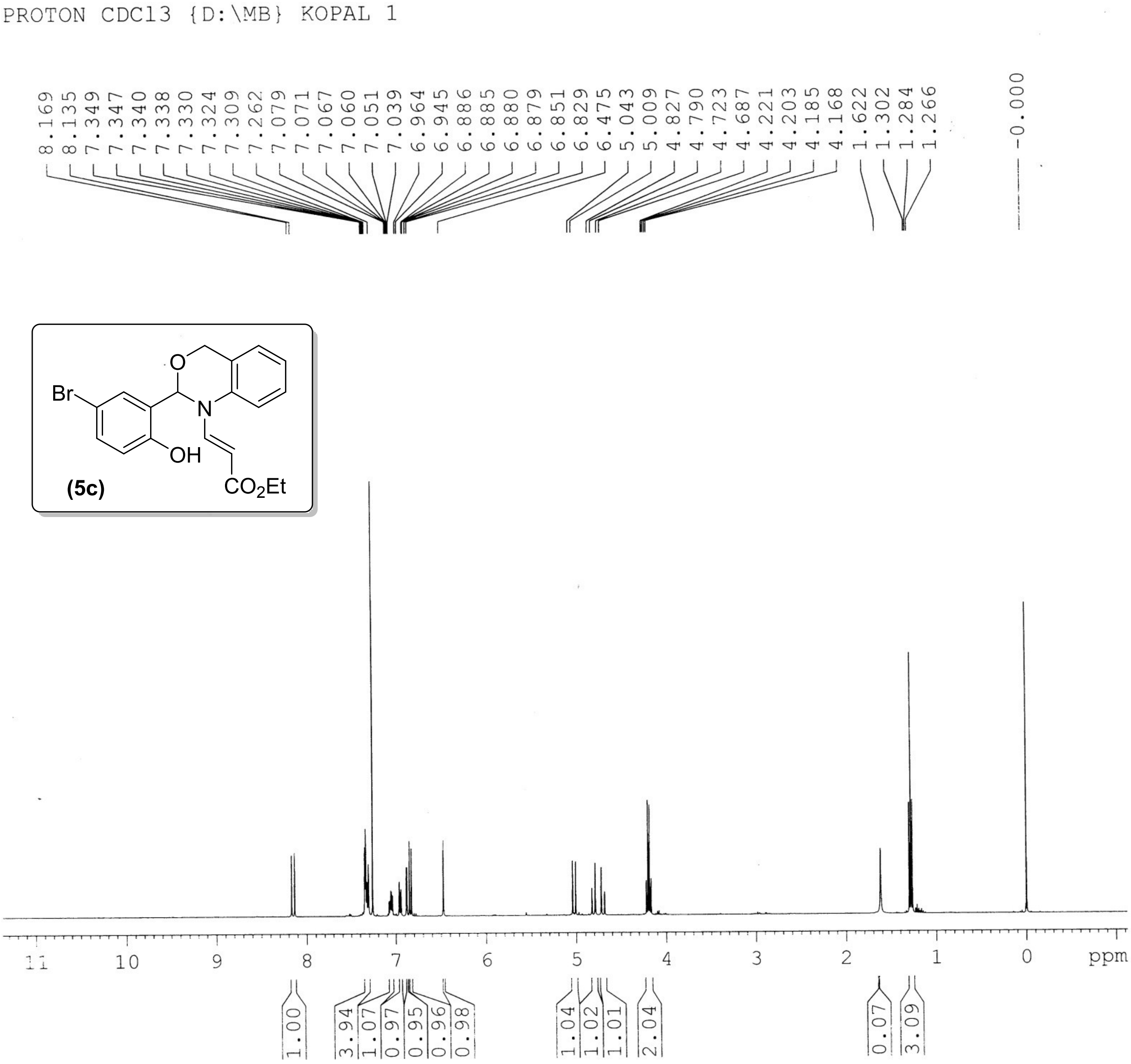
PROTON CDCl3 {D:\MB} KOPAL 1
8.169
8.135
7.349
7.347
7.340
7.338
7.330
7.324
7.309
7.262
7.079
7.071
7.067
7.060
7.051
7.039
6.964
6.945
6.886
6.885
6.880
6.879
6.851
6.829
6.475
5.043
5.009
4.827
4.790
4.723
4.687
4.221
4.203
4.185
4.168
1.622
1.302
1.284
1.266
-0.000
Br
O
N
OH
CO_2Et
(5c)
11
10
9
8
7
6
5
4
3
2
1
0
ppm
1.00
3.94
1.07
0.97
0.95
0.96
0.98
1.04
1.02
1.01
2.04
0.07
3.09

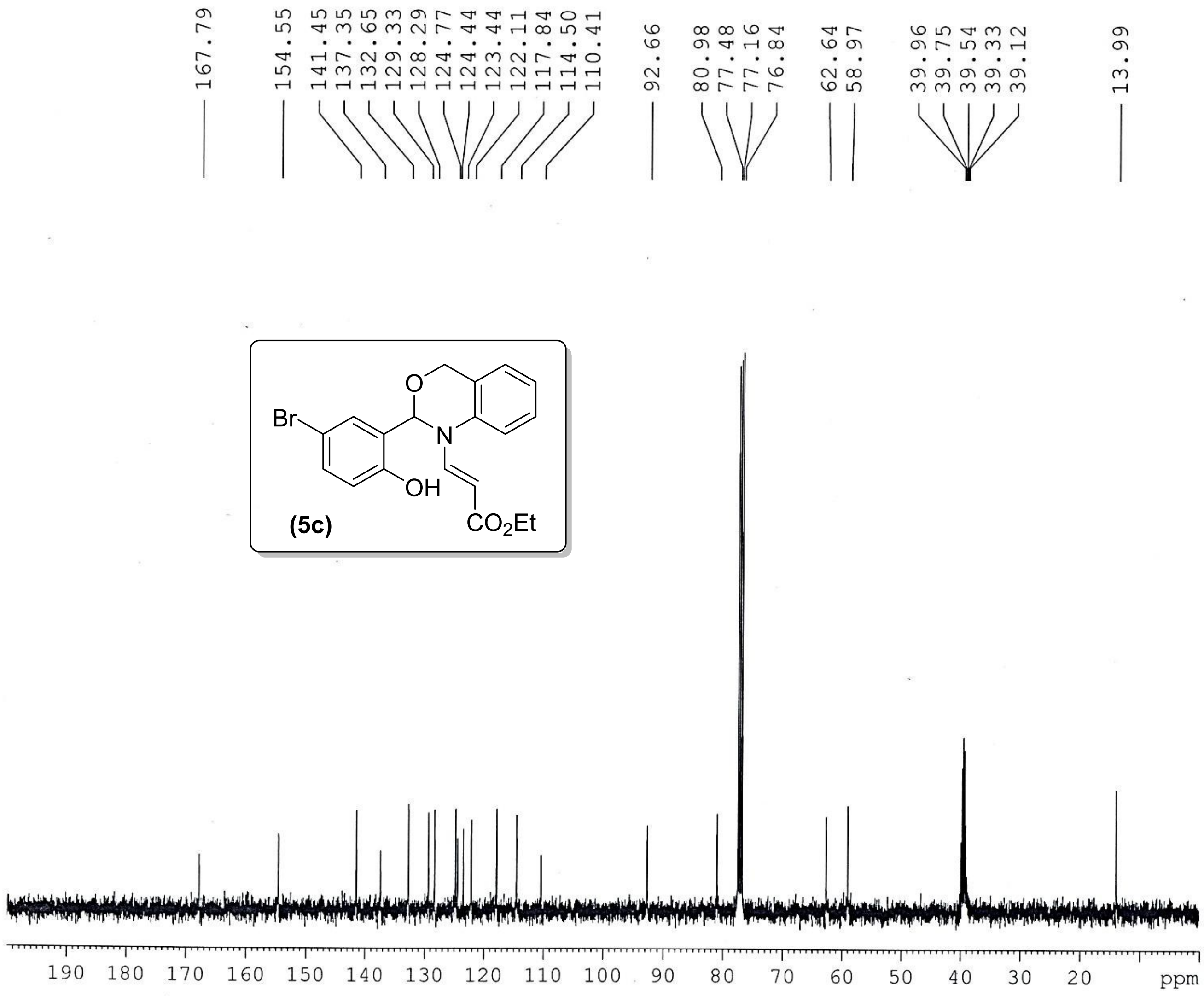
167.79
154.55
141.45
137.35
132.65
129.33
128.29
124.77
124.44
123.44
122.11
117.84
114.50
110.41
92.66
80.98
77.48
77.16
76.84
62.64
58.97
39.96
39.75
39.54
39.33
39.12
13.99
Br
O
N
OH
(5c)
CO_2Et
190 180 170 160 150 140 130 120 110 100 90 80 70 60 50 40 30 20
ppm

**Sample Name**	MM-105	**Position**		**Instrument Name**	Q-TOF	**User Name**	QTOF-PU\admin
**Inj Vol**	-1	**InjPosition**		**SampleType**	Sample	**IRM Calibration Status**	Success
**Data Filename**	MM-105.d	**ACQ Method**	Pondicherry Universi			**Acquired Time**	

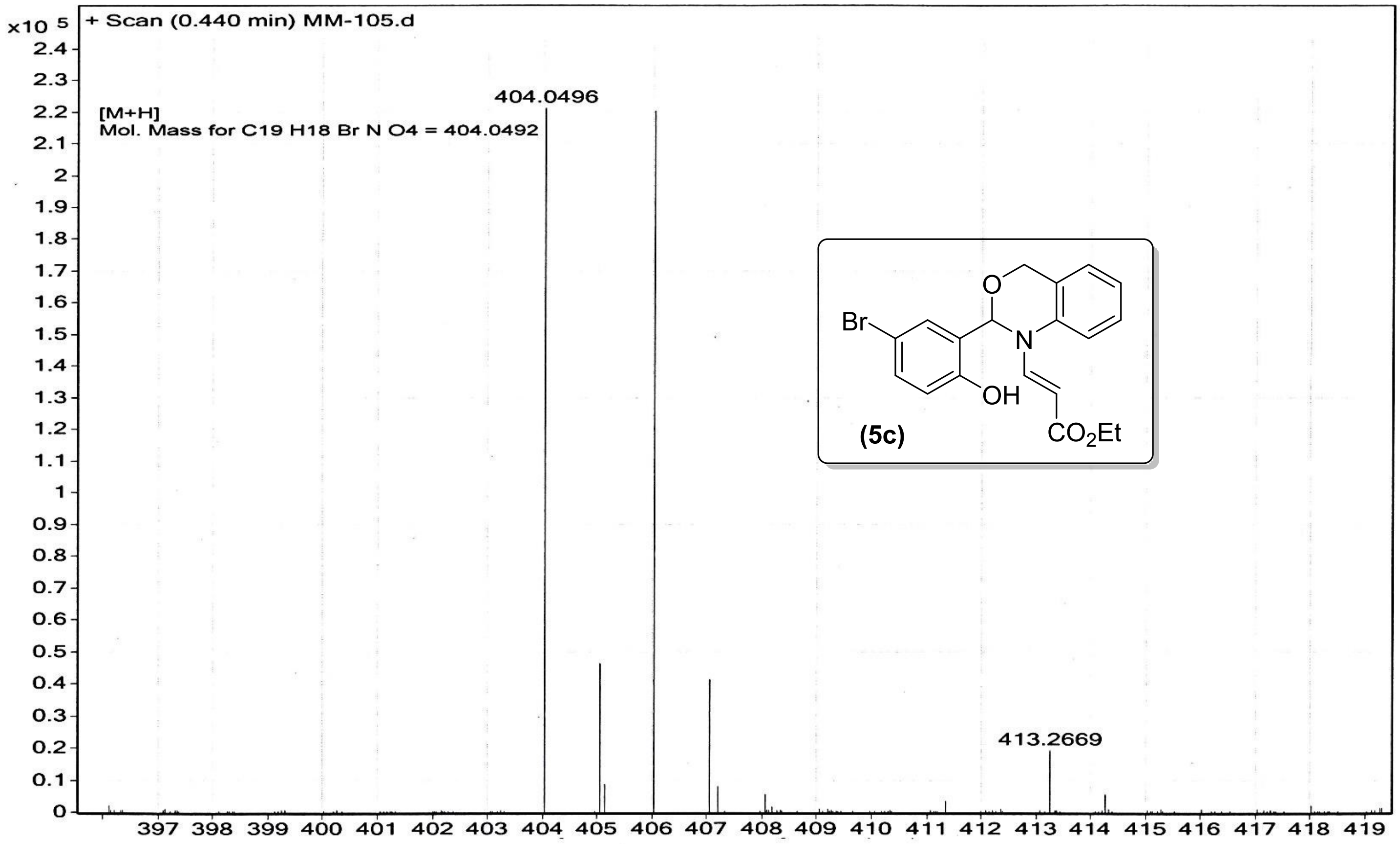

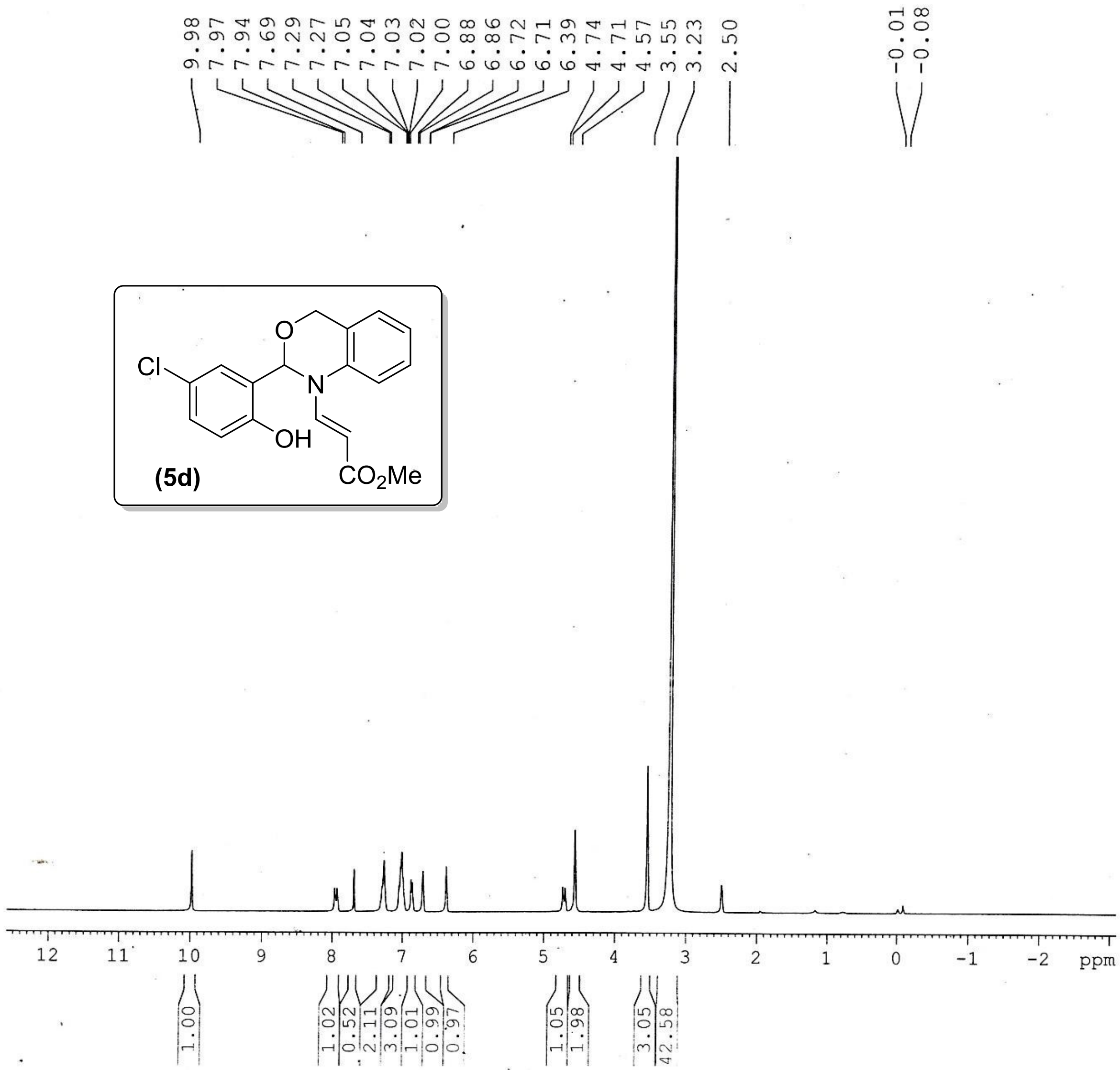
9.98
7.97
7.94
7.69
7.29
7.27
7.05
7.04
7.03
7.02
7.00
6.88
6.86
6.72
6.71
6.39
4.74
4.71
4.57
3.55
3.23
2.50
-0.01
-0.08
Cl
O
N
OH
CO_2Me
(5d)
12
11
10
9
8
7
6
5
4
3
2
1
0
-1
-2
ppm
1.00
1.02
0.52
2.11
3.09
1.01
0.99
0.97
1.05
1.98
3.05
42.58

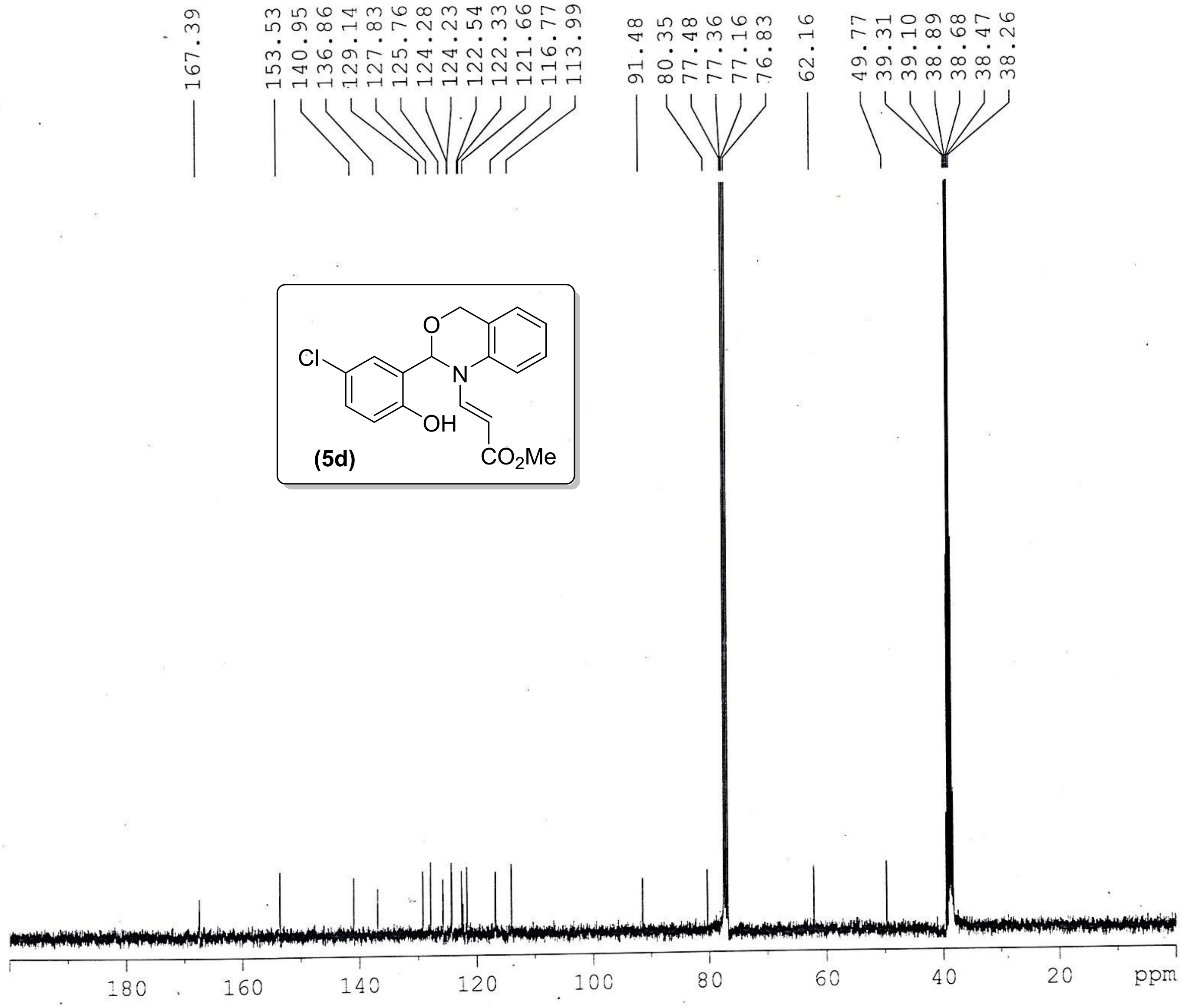
167.39
153.53
140.95
136.86
129.14
127.83
125.76
124.28
124.23
122.54
122.33
121.66
116.77
113.99
91.48
80.35
77.48
77.36
77.16
76.83
62.16
49.77
39.31
39.10
38.89
38.68
38.47
38.26
O
Cl
N
OH
(5d)
CO2Me
180
160
140
120
100
80
60
40
20
ppm

**Sample Name**	MM-174	**Position**		**Instrument Name**	Q-TOF	**User Name**	QTOF-PU\admin
**Inj Vol**	-1	**InjPosition**		**SampleType**	Sample	**IRM Calibration Status**	Success
**Data Filename**	MM-174N.d	**ACQ Method**	Pondicherry Universi				

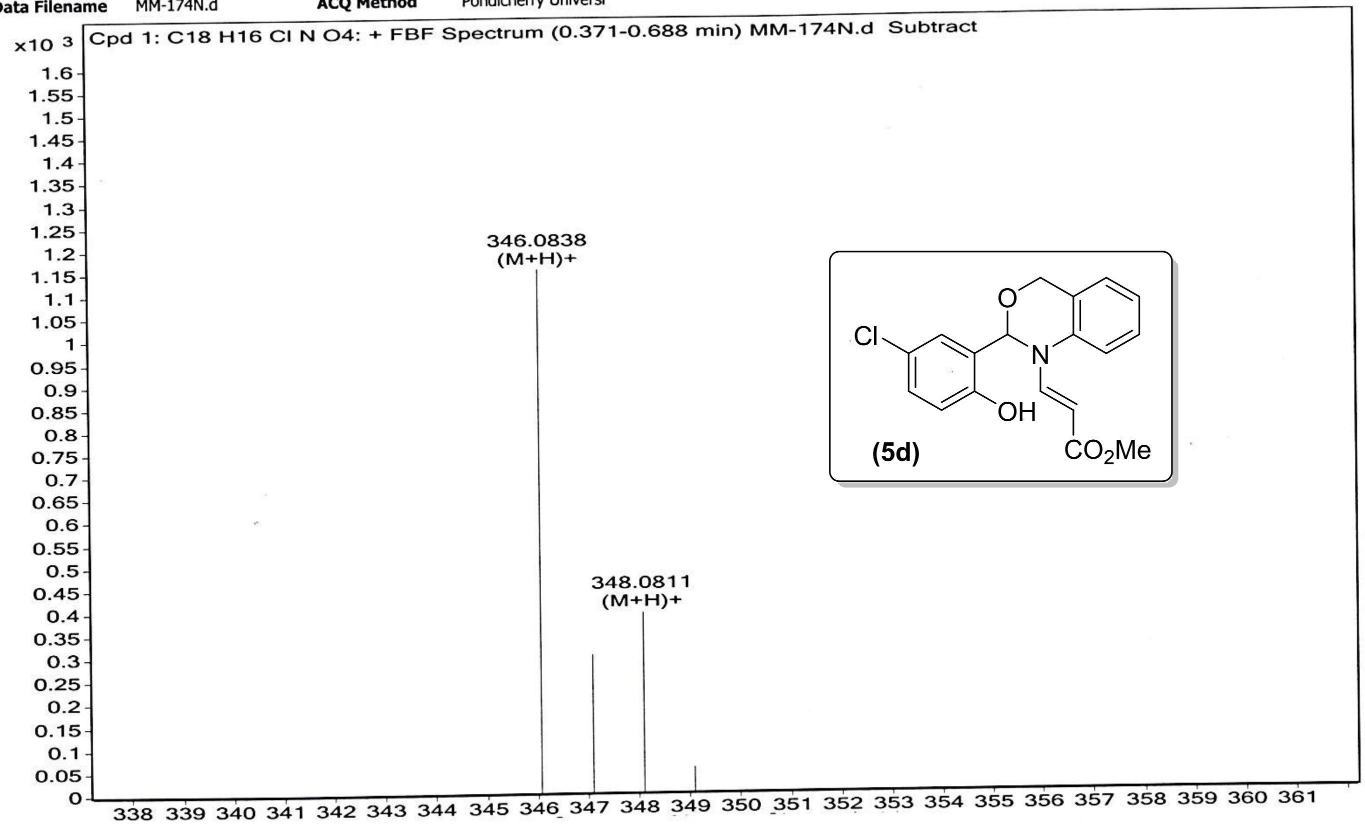

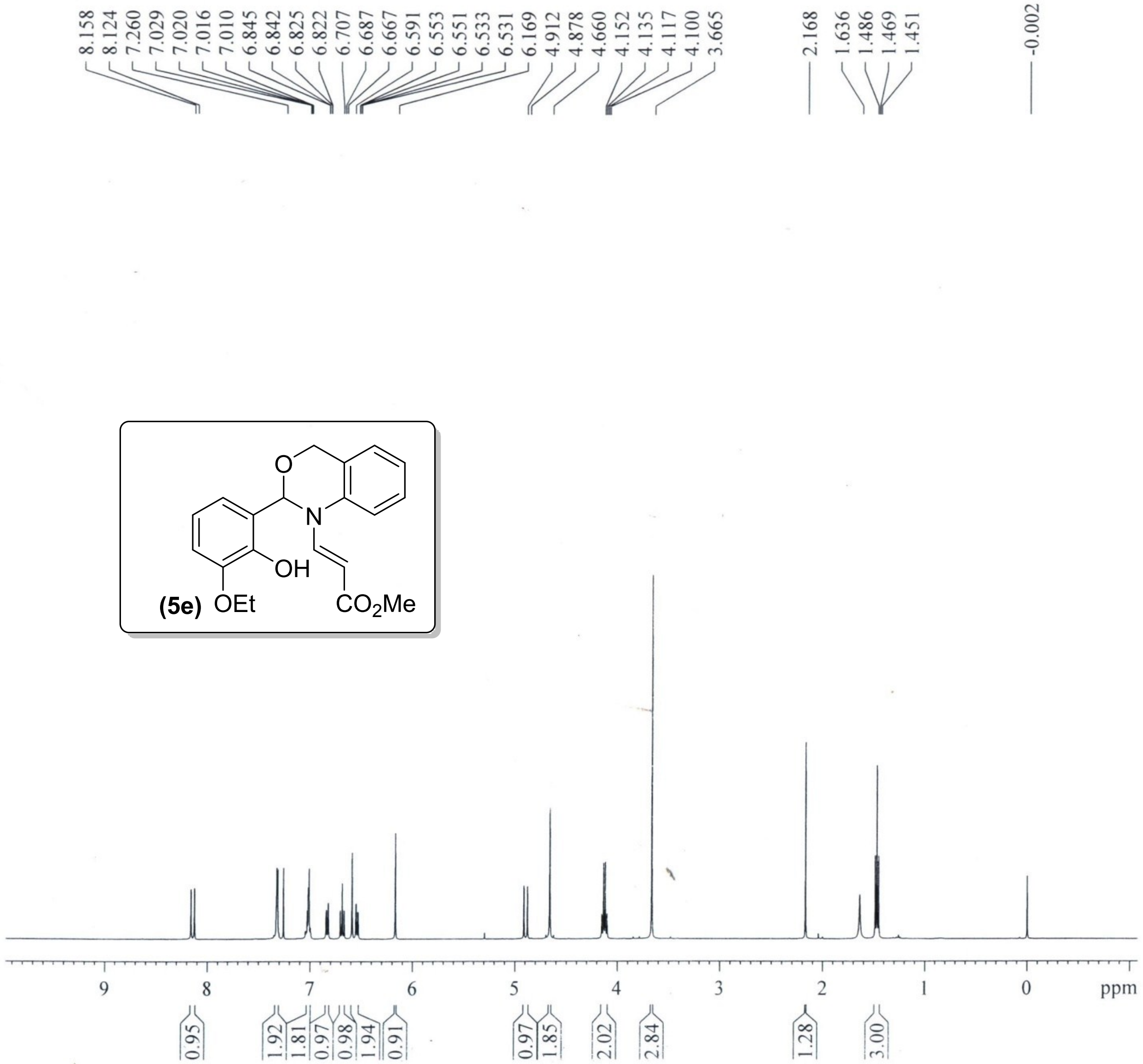
8.158
8.124
7.260
7.029
7.020
7.016
7.010
6.845
6.842
6.825
6.822
6.707
6.687
6.667
6.591
6.553
6.551
6.533
6.531
6.169
4.912
4.878
4.660
4.152
4.135
4.117
4.100
3.665
2.168
1.636
1.486
1.469
1.451
-0.002
O
N
OH
(5e)
OEt
CO2Me
9
8
7
6
5
4
3
2
1
0
ppm
0.95
1.92
1.81
0.97
0.98
1.94
0.91
0.97
1.85
2.02
2.84
1.28
3.00

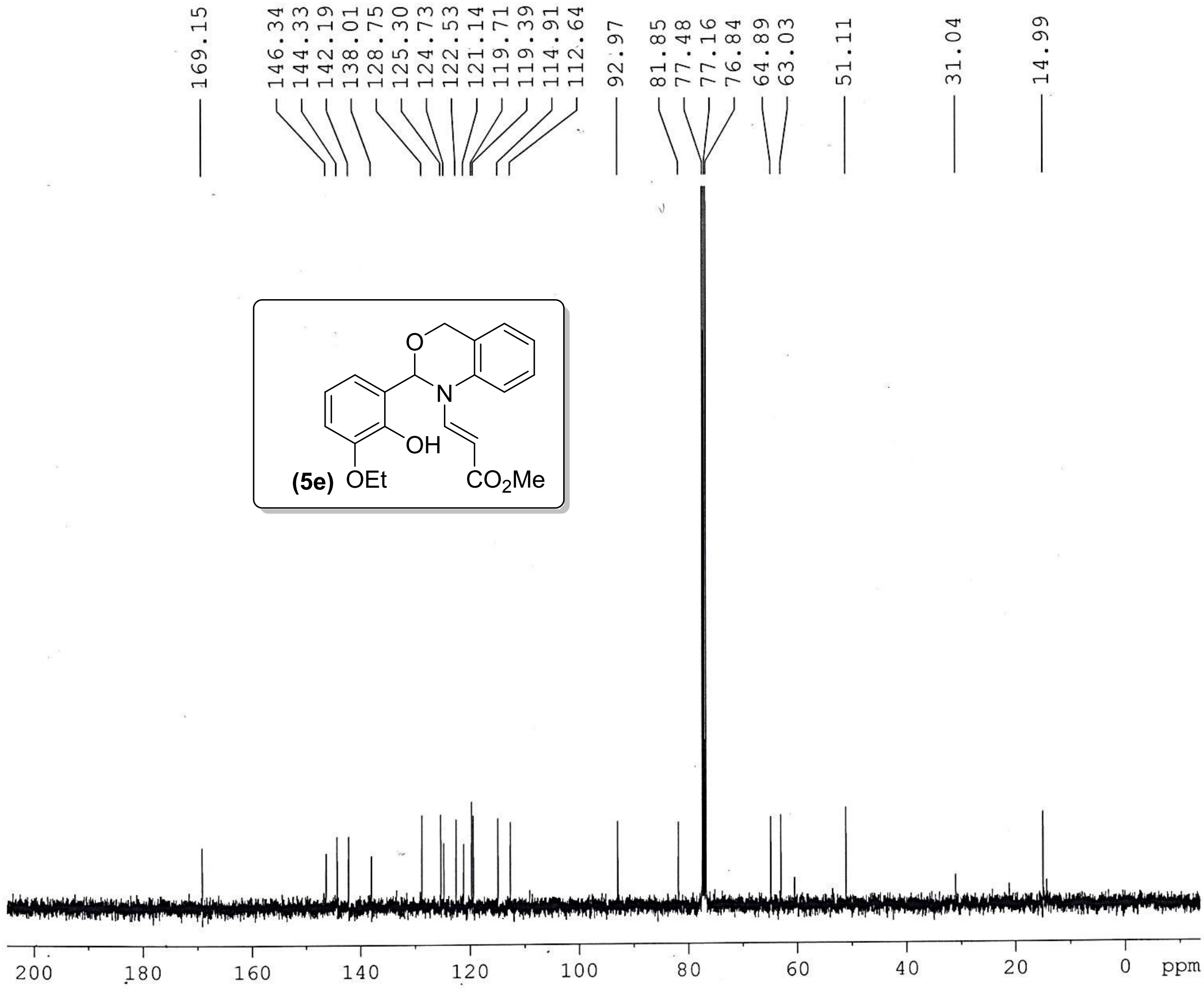
169.15
146.34
144.33
142.19
138.01
128.75
125.30
124.73
122.53
121.14
119.71
119.39
114.91
112.64
92.97
81.85
77.48
77.16
76.84
64.89
63.03
51.11
31.04
14.99
O
N
OH
(5e) OEt
CO2Me
200 180 160 140 120 100 80 60 40 20 0 ppm

**Sample Name**	MM-174	**Position**		**Instrument Name**	Q-TOF	**User Name**	QTOF-PU\admin
**Inj Vol**	-1	**InjPosition**		**SampleType**	Sample	**IRM Calibration Status**	Success
**Data Filename**	MM-174.d	**ACQ Method**	Pondicherry Universi				

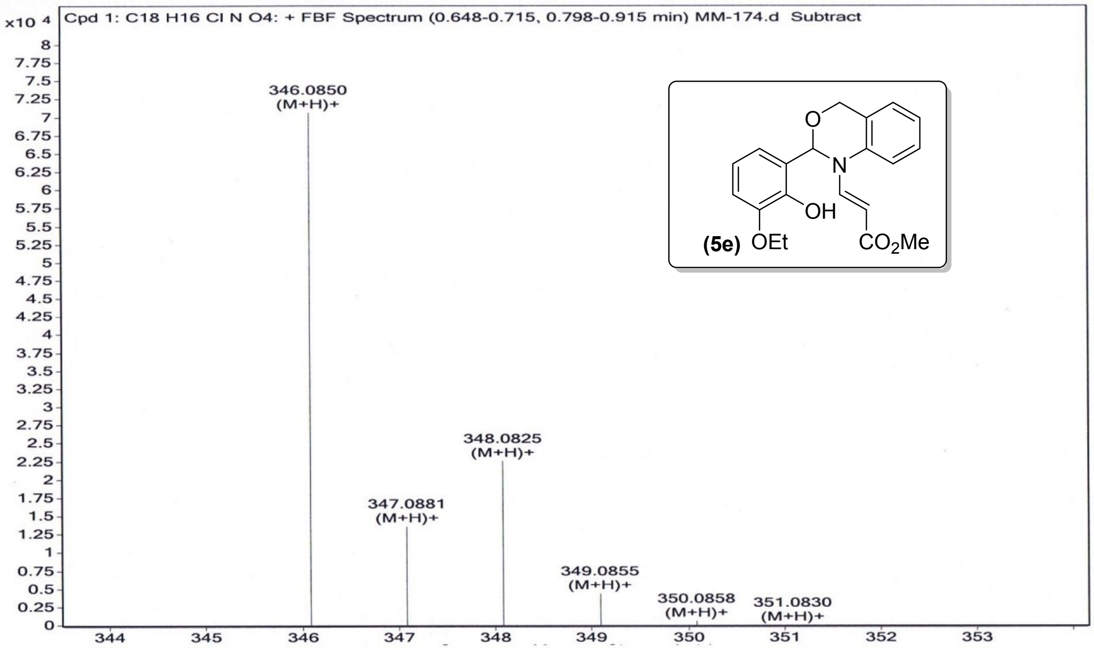

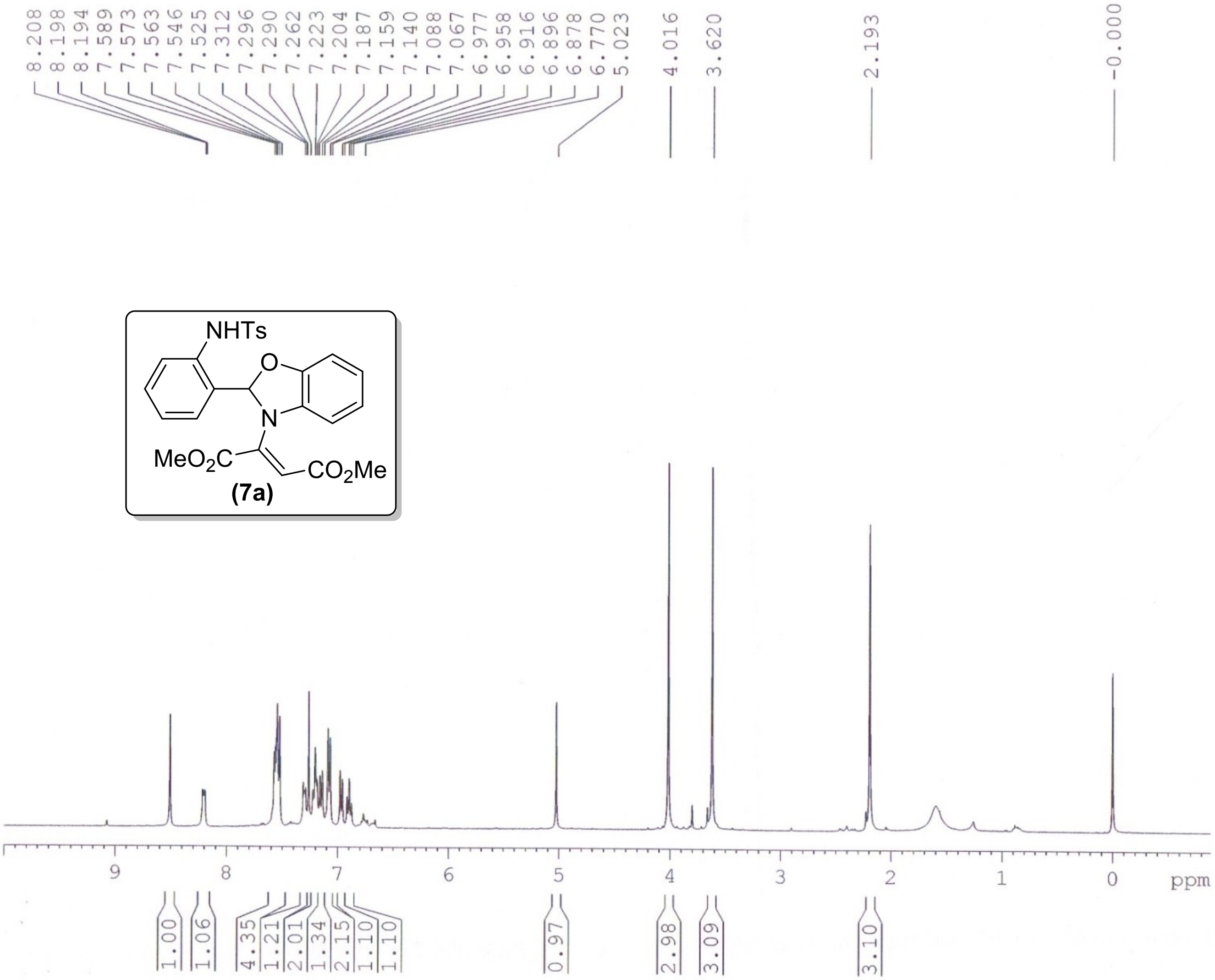
8.208
8.198
8.194
7.589
7.573
7.563
7.546
7.525
7.312
7.296
7.290
7.262
7.223
7.204
7.187
7.159
7.140
7.088
7.067
6.977
6.958
6.916
6.896
6.878
6.770
5.023
4.016
3.620
2.193
-0.000
NHTs
O
N
MeO2C
CO2Me
(7a)
9
8
7
6
5
4
3
2
1
0
ppm
1.00
1.06
4.35
1.21
2.01
1.34
2.15
1.10
1.10
0.97
2.98
3.09
3.10

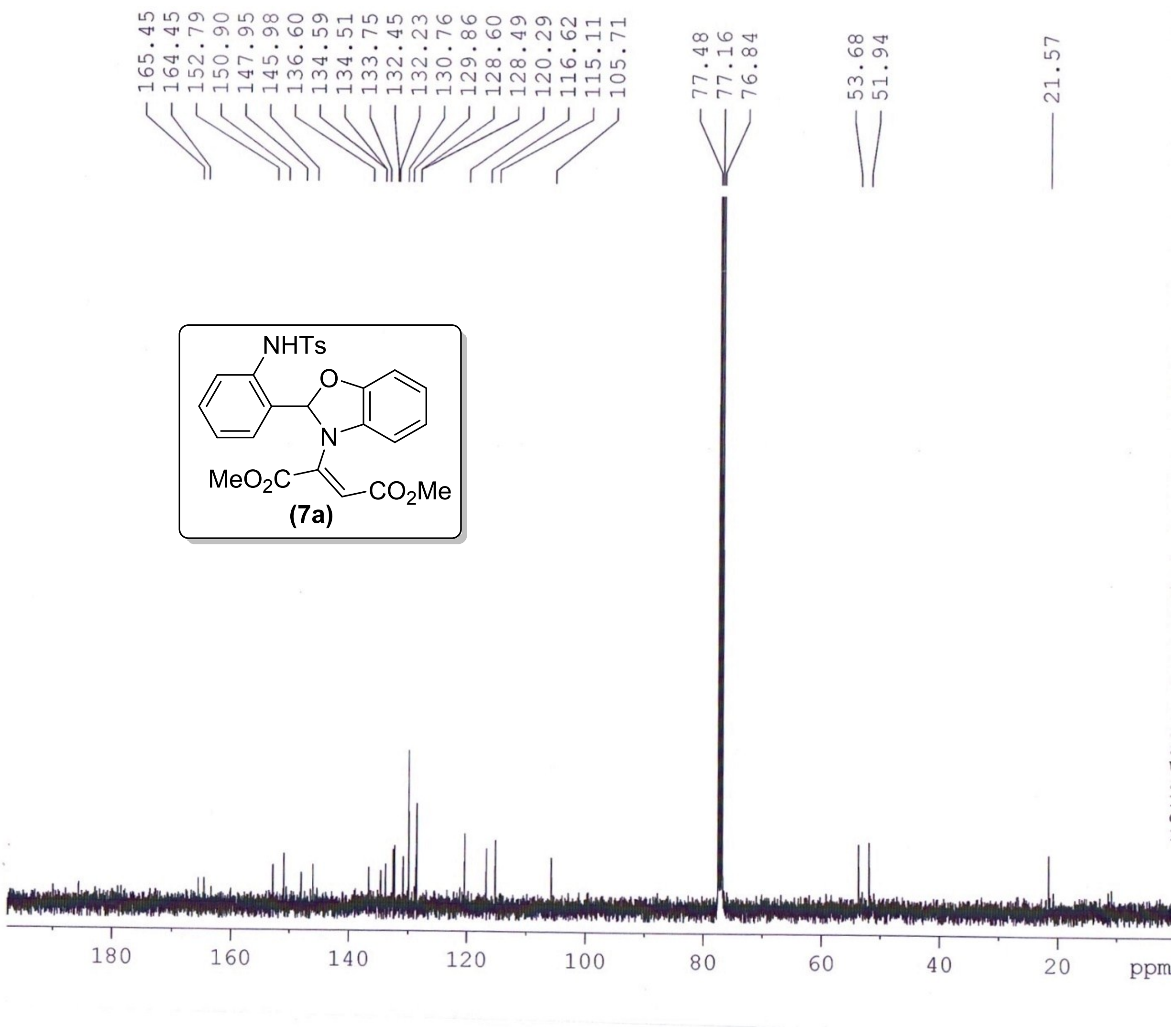
165.45
164.45
152.79
150.90
147.95
145.98
136.60
134.59
134.51
133.75
132.45
132.23
130.76
129.86
128.60
128.49
120.29
116.62
115.11
105.71
77.48
77.16
76.84
53.68
51.94
21.57
NHTs
O
N
MeO2C
CO2Me
(7a)
180
160
140
120
100
80
60
40
20
ppm

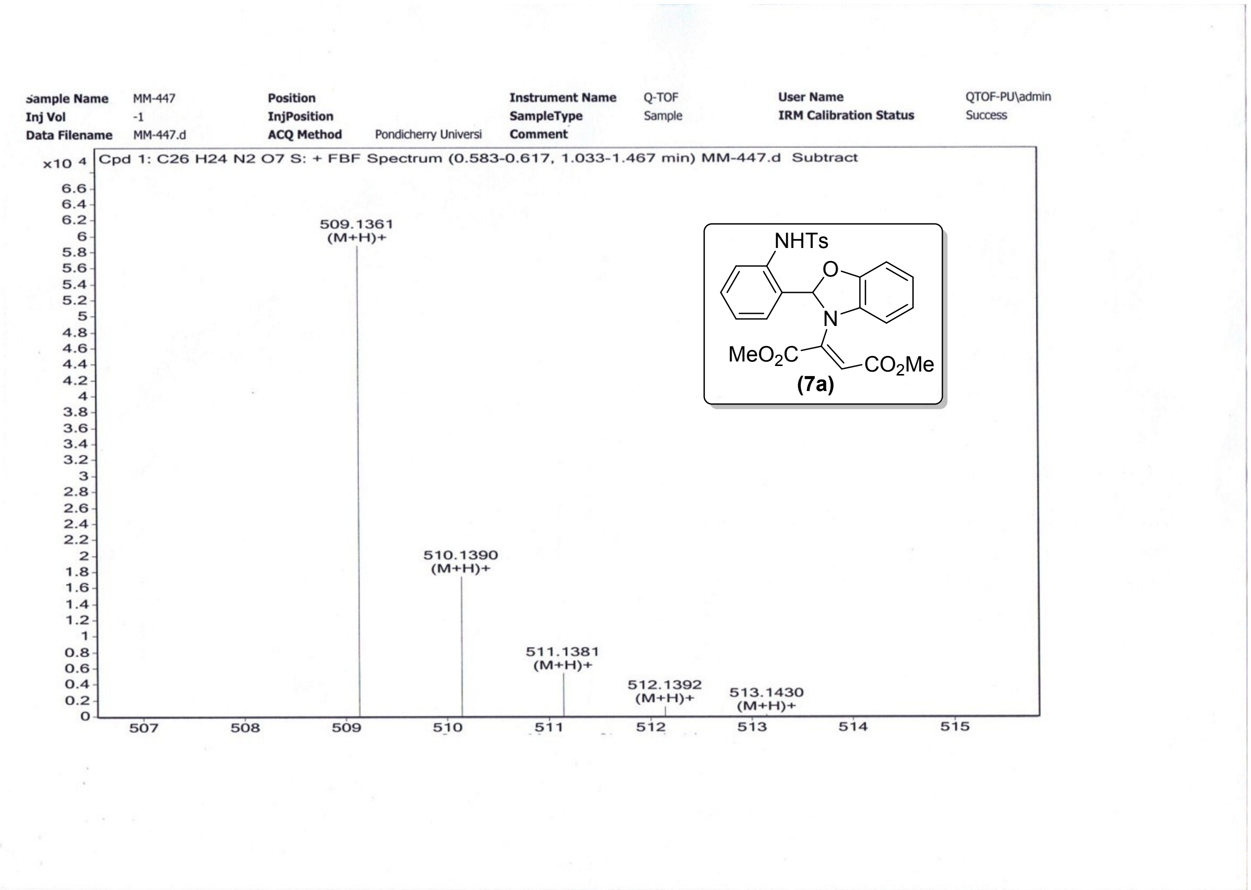

Sample Name MM-447
Position
Instrument Name Q-TOF
User Name QTOF-PU\admin
Inj Vol -1
InjPosition
SampleType Sample
IRM Calibration Status Success
Data Filename MM-447.d
ACQ Method Pondicherry Universi
Comment
Cpd 1: C26 H24 N2 O7 S: + FBF Spectrum (0.583-0.617, 1.033-1.467 min) MM-447.d Subtract
x10 4
509.1361 (M+H)+
510.1390 (M+H)+
511.1381 (M+H)+
512.1392 (M+H)+
513.1430 (M+H)+
NHTs
O
N
MeO2C
CO2Me
(7a)

PROTON CDCl3 {D:\MB} KOPAL 1

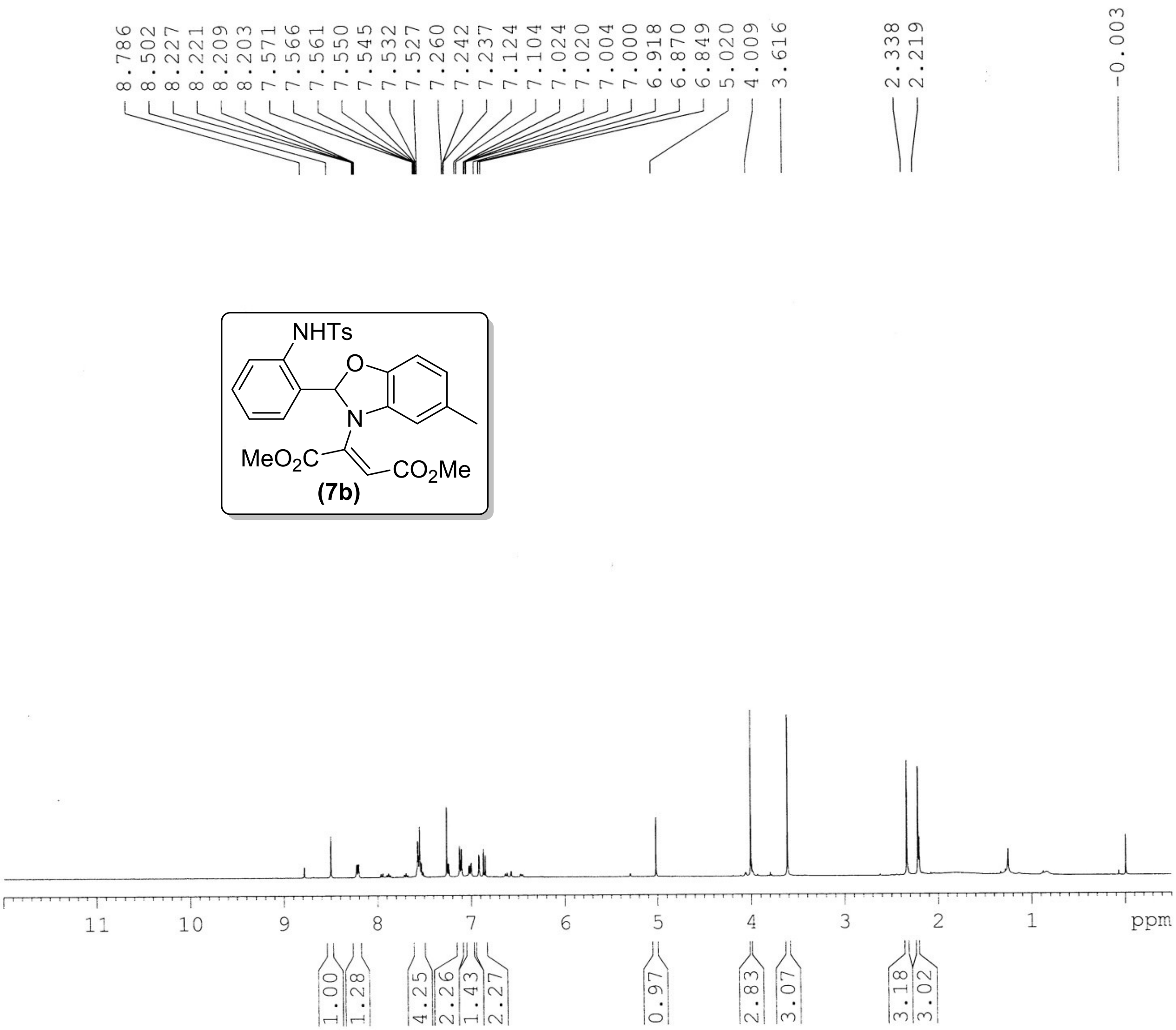

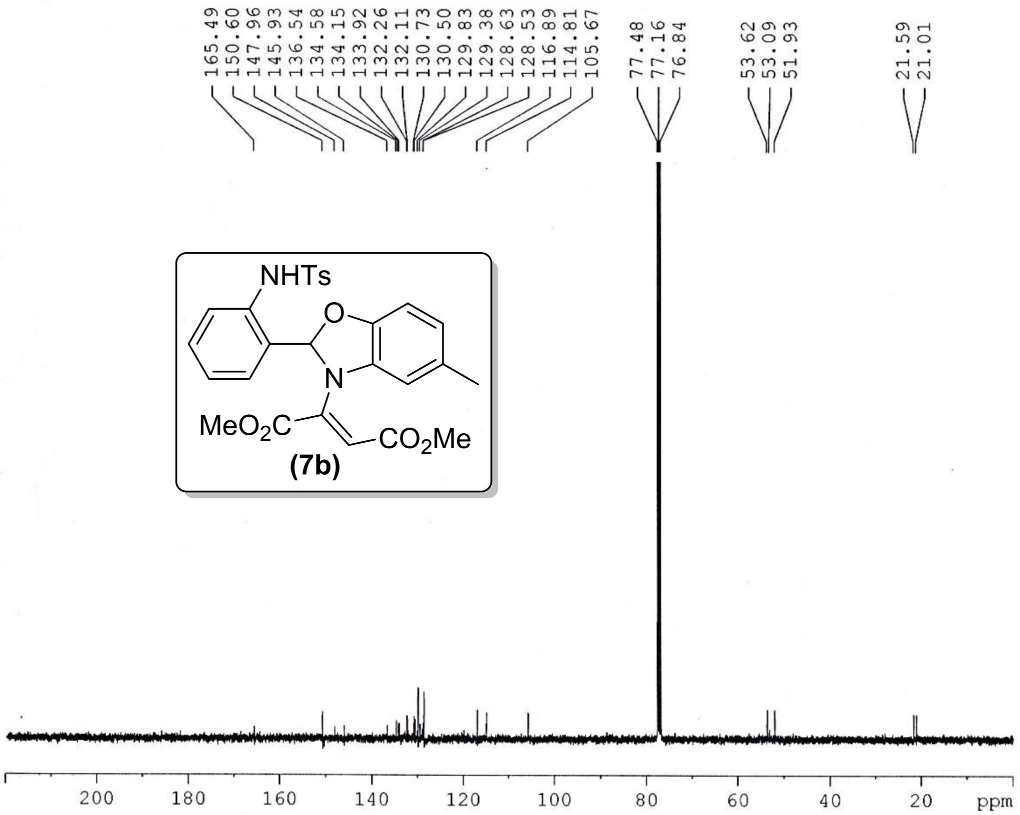

165.49
150.60
147.96
145.93
136.54
134.58
134.15
133.92
132.26
132.11
130.73
130.50
129.83
129.38
128.63
128.53
116.89
114.81
105.67
77.48
77.16
76.84
53.62
53.09
51.93
21.59
21.01
NHTs
O
N
MeO2C
CO2Me
(7b)
200
180
160
140
120
100
80
60
40
20
ppm

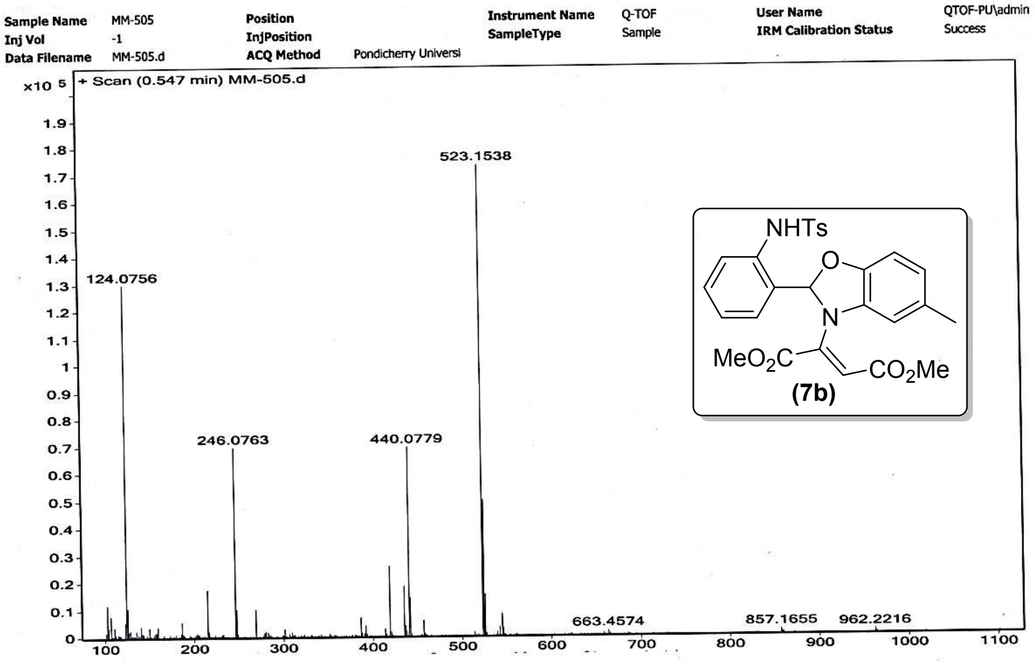
Sample Name MM-505
Inj Vol -1
Data Filename MM-505.d
Position
InjPosition
ACQ Method Pondicherry Universi
Instrument Name Q-TOF
SampleType Sample
User Name QTOF-PU\admin
IRM Calibration Status Success
x10 5
+ Scan (0.547 min) MM-505.d
523.1538
124.0756
246.0763
440.0779
663.4574
857.1655
962.2216
NHTs
O
N
MeO_2C
CO_2Me
(7b)
